# Comparative effectiveness of exercise interventions on arterial stiffness in individuals at risk for cardiovascular disease: a systematic review and network meta-analysis

**DOI:** 10.3389/fcvm.2025.1489382

**Published:** 2025-02-27

**Authors:** Ruo-Shan Wu, Yan Zhang, Xin-Wang Yuan, Xin Yan, Xiao-Lei Fu

**Affiliations:** ^1^ School of Physical Education, Hunan University of Science and Technology, Xiangtan, Hunan, China; ^2^ Department of Anesthesiology, Zhuzhou Central Hospital, Zhuzhou, Hunan, China; ^3^ College of Competitive Sports, Beijing Sport University, Beijing, China; ^4^ Department of Cardiovascular Medicine, Zhuzhou Central Hospital, Zhuzhou, Hunan, China

**Keywords:** arterial stiffness, blood pressure, high-risk population for cardiovascular disease, exercise intervention, network meta-analysis

## Abstract

**Background:**

Previous studies have shown that exercise can improve arterial stiffness (AS). However, it remains unclear which type of exercise is most effective for managing AS, particularly in individuals at high risk for cardiovascular diseases (CVD). This review aims to evaluate the effects of various exercises on AS and related variables in individuals at high risk for CVD.

**Methods:**

A comprehensive search strategy was employed to systematically explore MEDLINE (PubMed), Embase, Cochrane Library, EBSCOhost, and Web of Science to identify relevant studies. Inclusion criteria were: (1) randomized controlled trials; (2) participants with known CVD risk factors as per the American College of Sports Medicine guidelines; (3) interventions including interval training (INT), aerobic exercise (AE), resistance exercise, and combined exercise (CT); (4) control groups engaging in no intervention, routine care, or health education; (5) outcome measures of pulse wave velocity (PWV), systolic blood pressure (SBP), and diastolic blood pressure; and (6) studies published in English. Studies were assessed using the Cochrane risk of bias tool and analyzed with a random-effects network meta-analysis.

**Results:**

The review included 2,034 participants from 43 studies. Both CT [standardized mean difference (SMD) = −0.98, *p* < 0.001, *I*^2^ = 84%] and INT (SMD = −0.77, *p* < 0.001, *I*^2^ = 61%) significantly reduced PWV, but both showed considerable heterogeneity. INT (SMD = −0.382, *p* < 0.001, *I*^2^ = 45%) and AE (SMD = −0.369, *p* < 0.001, *I*^2^ = 43%) significantly reduced SBP. Surface under the cumulative ranking curve (SUCRA) showed that CT (SUCRA = 87.2) was the most effective for lowering PWV, while INT (SUCRA = 81.3) was the most effective for lowering SBP.

**Conclusion:**

In high-risk populations for CVD, CT was most effective in improving AS, while INT demonstrated the greatest reduction in SBP. AE showed greater benefits at moderate to low intensities. Due to significant heterogeneity in CT, its results should be interpreted with caution. Further research with larger sample sizes is needed to confirm these findings.

## Graphical Abstract

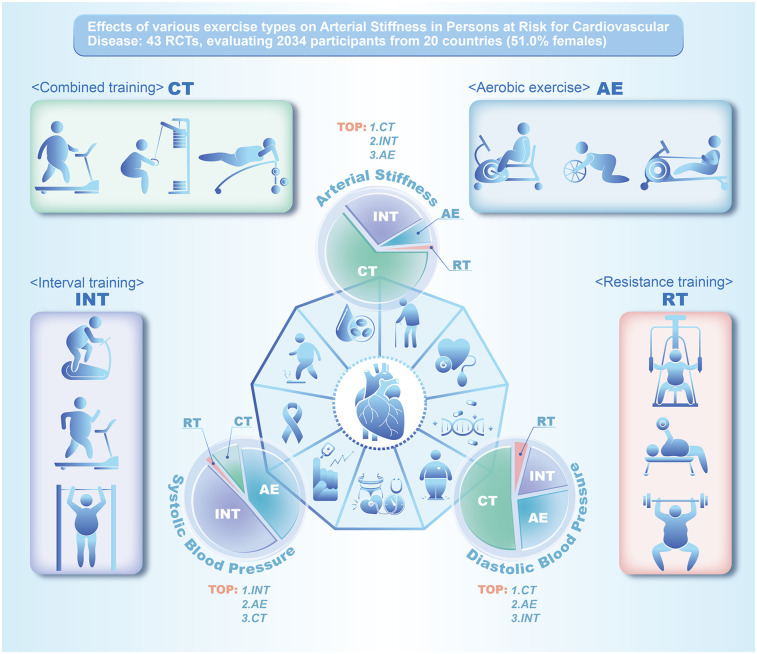

## 1 Introduction

Arterial stiffness (AS) is a critical predictor of cardiovascular disease (CVD) risk, independent of other factors. An increase of 1 m/s in pulse wave velocity (PWV), a primary measure of AS, corresponds to a 12%–14% rise in cardiovascular events and a 13%–15% increase in CVD-related mortality (1). While drug therapy is commonly used to manage AS, its early detection remains challenging. Consequently, preventive strategies, such as dietary changes and exercise, are increasingly important.

Research shows that exercise can improve AS by enhancing arterial remodeling and endothelial function, reducing sympathetic nervous system tone, and lowering inflammatory cytokines (2). However, there is debate about the most effective exercise interventions for different populations, particularly those at high risk for CVD.

Aerobic exercise (AE) is often recommended for improving vascular function due to its effects on vasodilators like calcium (Ca^2+^), potassium (K^+^), hydrogen ions (H^+^), and carbon dioxide (CO_2_) (3). Interval training (INT) may offer even greater benefits for AS and blood pressure than AE (4). Resistance training (RT) presents mixed results; high-intensity RT might increase blood pressure while reducing AS (5), but it could also induce arteriosclerosis in younger individuals without affecting older adults (3, 6). Combined training (CT), which includes both AE and RT, is considered highly effective for improving AS (7), with the order of exercises affecting results—CT with AE following RT shows better outcomes than the reverse (8).

Several systematic reviews have examined the impact of various exercise interventions on AS. Saz-Lara et al. (3) reviewed AE, RT, INT, CT, physical and mental exercises, and stretching in healthy adults. Montero et al. (9, 10) focused on AE in obese and hypertensive individuals, while Miyachi (6) and Evans et al. (11) reviewed RT in both healthy individuals and those at risk for CVD. Way et al. (12) assessed high-intensity INT and moderate-intensity AE, while Ashor et al. (13) and Zhang et al. (2) reviewed AE, RT, and CT. Ashor et al. focused on healthy and at-risk populations, while Zhang et al. focused on CVD patients. However, both studies lacked detailed analysis of long-term cardiac function and specific subpopulations. The current review addresses these gaps because many existing reviews either focus on narrow groups or lack comprehensive comparisons for high-risk CVD populations.

This study aims to address this gap by referring to the American College of Sports Medicine (ACSM) guidelines to identify high-risk populations, including older adults, those with diabetes, obesity, hypertension, and metabolic syndrome, and postmenopausal women. We conducted a systematic review and network meta-analysis (NMA) of randomized controlled trials (RCTs) to evaluate the effects of INT (14–25), AE (15, 26–35), RT (31, 36–43), and CT (44–55) on AS in these high-risk populations. In addition, we assessed systolic blood pressure (SBP) and diastolic blood pressure (DBP) as secondary outcomes. Our goal is to provide practical recommendations for managing AS in individuals at elevated risk for CVD.

## 2 Methods

### 2.1 Registration

The study protocol was registered with PROSPERO (CRD42023417622) (56). This systematic review and NMA were conducted in accordance with the guidelines provided by the Preferred Reporting Items for Systematic Reviews and Meta-Analyses statement (PRISMA-NMA).

### 2.2 Literature search strategy

The MEDLINE (PubMed), Embase, Cochrane Library, EBSCO, and Web of Science databases were systematically searched for relevant articles using a comprehensive electronic search strategy, as shown in Supplementary Appendix 2. The search strategy was based on key phrases related to the PICOS tool: (P) Population: “Hypertension” OR “obesity” OR “Type 2 diabetes” OR “T2D” OR “metabolic syndrome” OR “Older persons over 60 years”; (I) Intervention: “physical activity” OR “training” OR “aerobic exercise” OR “moderate intensity continuous training” OR “moderate interval training” OR “high interval training” OR “resistance training” OR “strength training” OR “combined training” or “sprint interval training” or “high intensity interval training”; (C) Comparator: “control group” or “no exercise” or “usual care”; (O) Outcomes: “arterial stiffness” or “pulse wave velocity” or “PWV”; and (S) Study type: “randomized controlled trial”, “randomized”, “placebo”, “RCT”. The search was limited to English-language articles published between the inception of the databases up to February 2024. We included RCTs comparing different types of exercise on AS in high-risk populations of CVD. In addition, we retrieved similar review articles and screened their references to maximize the completeness of the sources.

### 2.3 Study selection

Duplications were initially eliminated using EndNote X9 software (Clarivate Analytics, Philadelphia, PA, USA). Subsequently, two researchers (R-SW and X-LF) independently screened titles and abstracts to identify all potentially relevant studies. The same two reviewers independently identified and assessed studies that met the inclusion criteria. Disagreements were resolved through discussion or consultation with a third expert (XY), if necessary. The detailed inclusion criteria were as follows: (1) all studies had to be RCTs; (2) subjects had known risk factors associated with CVD according to the American College of Sports Medicine guidelines; (3) interventions included INT, AE, RT, and CT; (4) the comparator received no intervention, usual care, or health education; (5) outcomes included PWV, SBP, and DBP; and (6) the studies had to be written in English.

The exclusion criteria were as follows: (1) the subjects of the study were healthy adults; (2) the type of experiment was animal experiments or randomized crossover experiments; (3) the effect of exercise intervention was acute (<3 weeks); (4) incomplete data or no control group; (5) returned reviews, duplicate publications, letters to the editor, and meta-analysis articles.

### 2.4 Exercise categories

In the included randomized controlled trials, exercise interventions comprised INT, AE, RT, and CT. As depicted in Table 1, we operationally defined these four modalities of exercise intervention as follows.

**Table 1:** Definition of the types of exercise.

Type	Definition
HIIT	Frequency: 3–5 times per week
	Intensity: >90% HR max/85% V˙O2max/85% V˙O2max
	Duration per session: ≥60 min per session
	Interval time: <40 s
	Any traditional intermittent interval training mode that involves short bursts of intense exercise interspersed with brief periods of rest or low-intensity exercise between different exercise sets (57)
MIIT	Frequency: 3–4 times per week
	Intensity: 75%–90% HR max/65%–85% V˙O2max/65%–85% HRR
	Duration per session: 30–59 min per session
	Interval time: 40–90 s
	Any traditional intermittent interval training mode that involves short bursts of intense exercise interspersed with brief periods of rest or low-intensity exercise between different exercise sets (57)
AE	Frequency: 3–5 times per week
	Intensity: >45% VO_2_max or >50% HRR or >65% HRmax
	Duration per session: 30–60 min per session
	Refers to continuous, moderate-intensity activity performed at a steady pace for an extended duration, typically lasting over 20 min (e.g., walking, running, cycling, rowing, swimming, aerobics, elliptical training, and step exercises)
RT	Frequency: 2–3 times per week
	Intensity: ≥50% 1RM
	Duration per session: 30–60 min per session
	Involves exercises that use resistance to build strength and muscle, such as weightlifting, bodyweight exercises, and resistance band training (57)
CT	A combination of AE and RT/INT
CON	No exercise or usual care or slight strength

INT, interval training; MIIT, moderate interval training; HIIT, high interval training; AE, aerobic exercise; RT, resistance training; CT, combined training; CON, control; HRR, heart rate reserve; HRmax, maximum heart rate; RM, repetition maximum; VO_2_max, maximal oxygen uptake.

### 2.5 Outcomes

The primary outcome assessed in this study included PWV, with secondary outcomes being SBP and DBP. There are several types of PWV: carotid–femoral pulse wave velocity (cfPWV), brachial-ankle pulse wave velocity (baPWV), central pulse wave velocity (cPWV), central-brachial pulse wave velocity (cbPWV), etc. In general, cfPWV is considered the first gold-standard measure of AS. The cfPWV is defined as the ratio of the distance between the measurement sites to the time difference for the distal pulse wave to reach the measuring sites. The cutoff point for AS was defined as a PWV value exceeding 10 m/s (58).

In this study, PWV primarily refers to the measurement of cfPWV. Since we also included baPWV indicators, we implemented a data transformation method to reconcile the measurement discrepancies between these two metrics. Specifically, we utilized the regression equation cfPWV=0.65×baPWV+2.1 to achieve accurate data correction (59). This method effectively addresses the variations in measurements, thereby improving the reliability of our results. The term SBP refers to the maximum arterial pressure during ventricular contraction, while DBP represents the arterial pressure when the heart is in a relaxed state. Pulse pressure (PP) is defined as the difference between SBP and DBP (60). The criteria for Stage I hypertension were defined as SBP of 130–139 mmHg and/or DBP of 80–89 mmHg. These parameters were incorporated into the outcomes and served as the indicators for assessing AS.

### 2.6 Data extraction

The data were extracted independently by two investigators (R-SW and XY), with any disagreements resolved through consensus or the input of a third author (X-LF) if necessary. Information collected included the first author, publication year, country, subject characteristics (sample size, gender, age, PWV, SBP, DBP, and concomitant diseases), intervention details (exercise type, intensity, duration, frequency, and supervision status), and outcome measures reported in each eligible study, as shown in Supplementary Appendix 4. In cases where information was insufficiently provided by studies included in this review article, email communication was initiated to obtain missing values.

### 2.7 Risk of bias and GRADE assessment

The risk of bias (ROB) of the included studies was evaluated by two researchers based on the Cochrane Risk of Bias assessment tool (61), which includes the following seven domains, encompassing seven distinct domains: (a) allocation generation, (b) allocation concealment, (c) blinding of participants and personnel, (d) blinding of outcome assessment, (e) handling of incomplete outcome data, (f) freedom from selective reporting bias, and (g) other forms of bias. These key domains were considered key domains for assessing the risk of bias. However, due to the inherent difficulty in blinding participants to an exercise intervention, this component was not included in the overall risk of bias score. Subsequently, we categorized each study's overall risk of bias into three levels: low risk if none of the above domains were rated as high risk and ≤3 domains were rated as unclear; moderate risk if one study was rated as high risk or no study was rated as high risk but ≥4 studies were rated as unclear; and high risk for all other cases (62).

The Grading of Recommendations Assessment, Development and Evaluation (GRADE) framework was utilized to evaluate the certainty of the evidence contributing to the network estimates for both primary and secondary outcomes (63).

### 2.8 Data synthesis and statistical analyses

The experimental effect was estimated by combining the pre-to-post changes of both the experimental and CON. The standard deviation (SD) of the change value was calculated using the formula provided in the Cochrane Handbook (version 6.3) (64) [the formula is $SD_{\mathrm{change}}=$ $\sqrt{{\;\mathrm{SD}}_{\mathrm{baseline}}^{2}+{\mathrm{SD}}_{\mathrm{final}}^{2}D-(2\times \mathrm{Corr}\times SD_{\mathrm{baseline}}\times SD_{\mathrm{final}})}$].

Heterogeneity among studies was assessed using network estimates and pairwise meta-analytic techniques with Review Manager 5.3 (Nordic Cochrane, Denmark). A sensitivity analysis was also conducted to explore this heterogeneity (65). Pooled effect estimates were calculated using a random-effects model, and mean difference (MD) values were determined for PWV, SBP, and DBP. Heterogeneity was quantified using the *I*^2^ statistic and Cochran's *Q*-test, with significant heterogeneity defined as an *I*^2^ >50% or a *p*-value ≤0.10 (66). Publication bias was evaluated with a funnel plot and Begg's test.

Statistical analysis was performed using STATA 16.0 (STATA Corp, College Station, TX, USA) through a frequentist framework and random-effects multivariate NMA (67). Weighted mean differences were reported for continuous variables, with pooled effect estimates accompanied by 95% confidence intervals (CIs) and 95% prediction intervals.

Exercise interventions were compared using network geometry, with lines connecting nodes representing direct relationships. The size of each node and the thickness of connecting lines reflected the number of studies (68).

Inconsistencies were estimated using the loop-specific approach, node-splitting technique, and global inconsistency method to assess ring, local, and global inconsistencies (69). A *p*-value <0.05 indicated that the inconsistency requirement was met, permitting further NMA analysis (70). The transitivity assumption, which assumes valid indirect comparisons and uniform effect modifiers, was evaluated using a consistency model to ensure random allocation of interventions (70, 71). The network contribution diagram calculated the impact of each direct comparison on both individual and overall NMA results.

The cumulative ranking curve (SUCRA) was used to rank the effects of different exercise modalities (64). SUCRA values range from 0 to 100, with higher values indicating better outcomes (72). Publication bias in the network meta-analysis was assessed using a funnel plot and symmetry criterion.

## 3 Results

### 3.1 Literature selection

**Figure 1:**
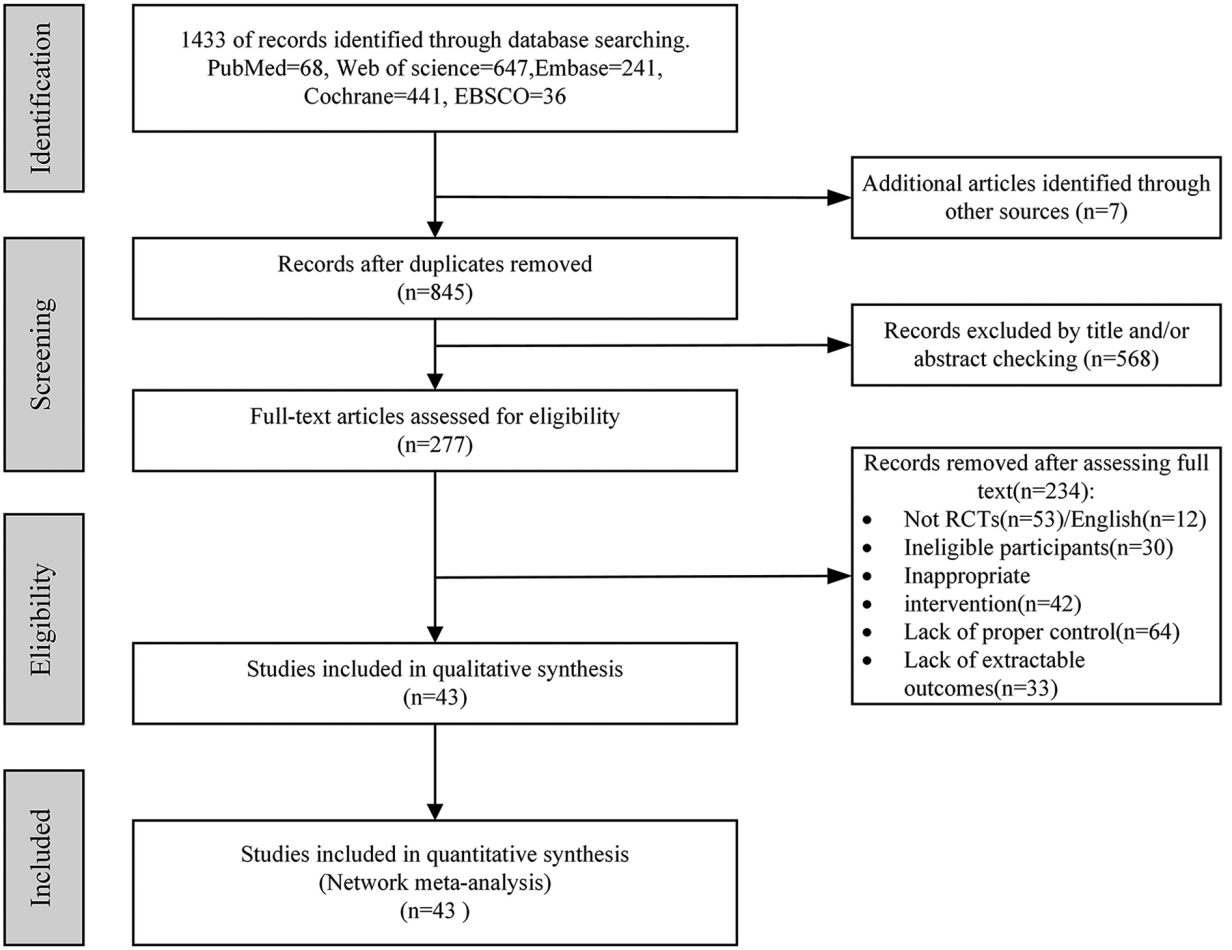
Preferred Reporting Items for Systematic Reviews and Meta-Analyses (PRISMA) flow diagram of each stage of the study selection. RCT, randomized controlled trial.

A total of 1,433 articles were retrieved from the database, while an additional 7 articles were obtained through alternative sources, resulting in a combined pool of 1,440 articles for screening. After eliminating duplicate entries, a total of 845 unique articles remained. Subsequently, after reviewing the titles and abstracts, 568 articles were excluded, leaving us with 277 potential candidates. Further evaluation of the full-text content led to the exclusion of an additional 234 articles, resulting in only 43 remaining articles that met our criteria. In conclusion, 43 eligible articles were ultimately identified as per Figure 1.

### 3.2 Characteristics of the included studies

Supplementary Appendix 4 provides details on the studies included, conducted between 2003 and 2023 across various regions: Asia (12), North America (13), South America (2), Europe (11), Oceania (1), Latin America (2), and Africa (2). The studies involved 2,034 high-risk CVD participants. The experimental group included 1,056 subjects, divided into the following groups: INT (365), AE (327), RT (149), and CT (215). The CON group included 978 subjects.

Participants were about 51% women, aged 10–75 years. Six studies focused solely on men, while 37 studies included both genders. All studies followed the ACSM guidelines for high-risk cardiovascular populations, considering obesity, diabetes, hypertension, and advanced age as risk factors.

Interventions included INT, AE, RT, and CT (AE + RT) at moderate to high intensity. Exercise duration ranged from 8 to 96 weeks, with activities such as running, cycling, rowing, swimming, stepping exercises, and resistance training. Sessions were held three to seven times per week, lasting 30–90 min each. Control groups received usual care or no exercise.

Of the studies, 33 used supervised exercise, 3 combined supervision with home-based interventions, and 7 had no supervision. Carotid–femoral PWV was measured using applanation tonometry (SphygmoCorCPV, ATCor Medical) or SphygmoCor software (version 9.0). Measurements were taken using a high-fidelity micromanometer (Millar Instruments), calculating PWV by dividing the distance between the carotid and femoral recording sites by the time delay between their pulse waves (71).

### 3.3 Results of ROB assessments

The ROB assessments for each study are presented in Supplementary Appendix 5. Two studies exhibited a high risk of bias in randomization serialization, while the concealment of allocation methods was reported in 15 studies. Blinding of outcome assessment had a low risk of bias in 30 studies, whereas 7 studies showed a high risk of bias regarding missing outcome values. Selective reporting demonstrated a low risk of bias in 10 studies. In addition, sample sizes smaller than *n* < 10 or significant errors in the measurement process were considered to have a high risk of other biases, with only 33 studies showing a low risk of bias. In summary, 24 articles were evaluated as having low ROB, while moderate and high ROB were observed in 14 and 5 articles, respectively.

### 3.4 Direct pairwise meta-analyses

#### 3.4.1 Primary outcome

**Figure 2:**
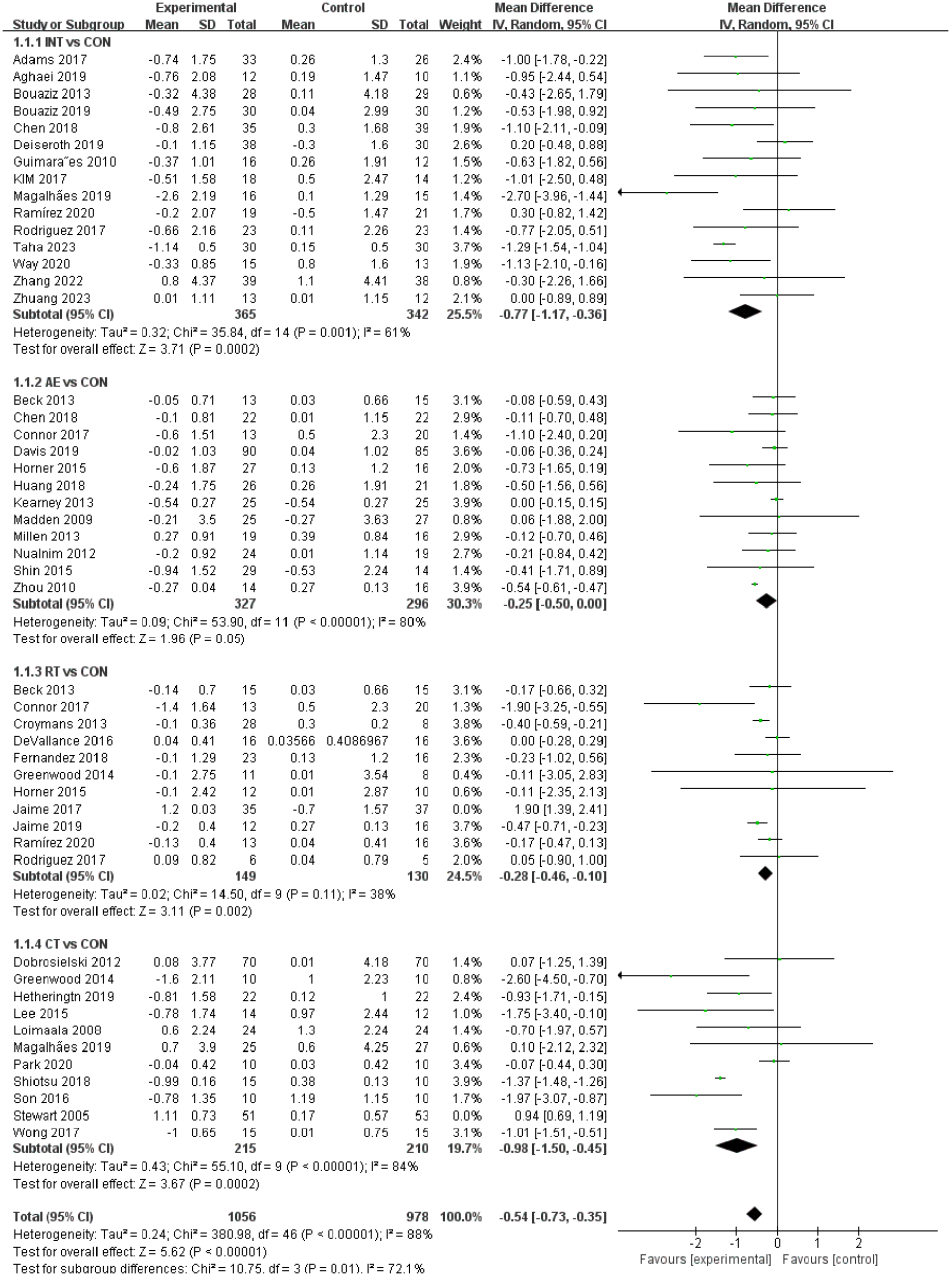
Forest plot of the effects of exercise versus control on pulse wave velocity.

As shown in Figure 2, the forest plots of different exercise intervention effects on PWV report the differences and heterogeneity of intervention effects of the four exercise interventions (INT, AE, RT, and CT) compared to the CON group.

Compared with the CON group, INT [standardized mean difference (SMD) = −0.77, *p* < 0.0001, 95% CI (−1.17 to −0.36), *I*^2^ = 61%], AE [SMD = −0.25, *p* = 0.000, 95% CI (−0.50 to 0.00), *I*^2^ = 80%], and CT [SMD = −0.98, *p* < 0.00001, 95% CI (−0.73 to −0.35), *I*^2^ = 84%] all significantly reduced PWV, with high heterogeneity. RT [SMD = −0.28, *p* = 0.11, 95% CI (−0.46 to −0.10), *I*^2^ = 38%] reduced PWV, with low heterogeneity. The Funnel plot and Begg's test in Supplementary Appendix 6 showed publication bias in the CT group (*p* < 0.001).

#### 3.4.2 Heterogeneity test

To further verify the sources of heterogeneity, we conducted sensitivity analyses, subgroup analyses, and meta-regression (65). First, in the sensitivity analysis, we sequentially identified the influence of each study on the overall heterogeneity and performed a meta-analysis on the remaining studies (*n* − 1). By observing changes in the combined results, we evaluated whether the original meta-analysis was significantly influenced by certain studies. The sensitivity analysis identified significant sources of heterogeneity in two intervention methods: INT and AE. Specifically, in AE, heterogeneity (*I*^2^ < 50%) was reduced after excluding either Deiseroth et al. (19) or Taha et al. (25), and in INT, heterogeneity was reduced after excluding Kearney et al. (27) (Supplementary Appendix 7.2). A detailed review of these three studies revealed that none of them used cfPWV as the AS measurement index, and all focused on elderly populations. This suggests that the measurement index of AS and the elderly population may be significant factors contributing to heterogeneity.

Second, since the sensitivity analysis did not identify any significant factors contributing to heterogeneity in CT, we conducted subgroup analyses and meta-regression based on PWV assessment site, intervention intensity, duration, content, and participant characteristics (e.g., gender, age) to explore potential sources of heterogeneity (Table 2). However, in these efforts, no significant factors were found. This suggests that a single low-quality study can increase heterogeneity by producing results that significantly differ from other similar studies. Therefore, we speculate that the study quality might have contributed to the heterogeneity (Supplementary Appendix 7.4). Therefore, the heterogeneity in CT is acknowledged as a limitation of this study and warrants further exploration in future research.

**Table 2:** Subgroup analyses assessing potential moderating factors for PWV in studies included in the meta-analysis.

Group	Studies		PWV (m/s)				
	Number	Reference	WMD (95% CI)	*I* ^2^	*p* overall change	*p* for sub dif	*p* for m
Intensity							
High	4	35, 36, 39, 40	0.32 (0.08 to 0.56)	96	0.000	0.000	0.695
Moderate	7	26, 31–34, 37, 38	−0.67 (−0.95 to −0.38)	61	0.018		
Duration (min)							
>2,000	9	31–34, 36–40	0.00 (−0.19 to 0.18)	89	0.000	0.000	0.037
<2,000	2	26, 35	−2.05 (−2.96 to −1.15)	97	0.000		
Content of intervention							
RT + AE	6	26, 34, 36–39	0.18 (−0.04 to 0.41)	92	0.000	0.000	0.691
AE + RT	3	32, 35, 40	−0.92 (−1.38 to −0.46)	95	0.000		
INT + RT	2	31, 33	−0.30 (−0.70 to 0.11)	67	0.081		
Sex							
Man (only)	4	34, 35, 37, 40	−0.61 (−1.01 to −0.20)	92	0.000	0.000	0.227
Woman (only)	2	32, 38	−1.49 (−2.12 to −0.86)	0	0.842		
Both sexes	5	31, 33, 36, 39	0.22 (0.00 to 0.43)	92	0.000		
Age (years)							
>60	5	34–38	0.38 (0.05 to 0.70)	96	0.000	0.000	0.691
<60	6	26, 31–33, 39, 40	−0.29 (−0.51 to −0.07)	72	0.004		
PWV assessment site							
cfPWV	5	26, 33, 35, 39, 40	−0.25 (−0.48 to −0.01)	90	0.000	0.000	0.377
baPWV	4	32, 34, 37, 38	−1.04 (−1.05 to −0.52)	66	0.055		
PWV	2	31, 36	0.72 (0.37 to 1.08)	97	0.000		

PWV, pulse wave velocity; WMD, weighted mean difference; *p* for sub dif, *p* for subgroup difference; *p* form, *p-*value for the meta-regression analyses between subgroups.

#### 3.4.3 Secondary outcomes

The forest plots illustrating the intervention effects of different exercise modalities on secondary indicators (SDP and SBP) are given in Supplementary Appendix 7. Among the four types of exercise interventions (INT, AE, RT, and CT), AE demonstrated a significant reduction in SBP, while CT showed a significant decrease in DBP. The remaining exercise interventions did not have a statistically significant effect on either SBP or DBP. Funnel plot analysis and Begg's test revealed publication bias for RT in both SBP and DBP (*p* < 0.001), whereas no publication bias was observed in other subgroups (Supplementary Appendix 5).

#### 3.5.4 Network meta-analysis

The primary focus of this study was on PWV (primary index) and blood pressure parameters, specifically SBP and DBP (secondary index), which were subjected to NMA. The supporting materials for PWV, SBP, and DBP primarily consisted of qualified network evidence plots, loop-special approaches, node-splitting techniques, global inconsistency assessments, network forest plots, network contribution plots, funnel plots, and cumulative ranking plots.

**Figure 3:**
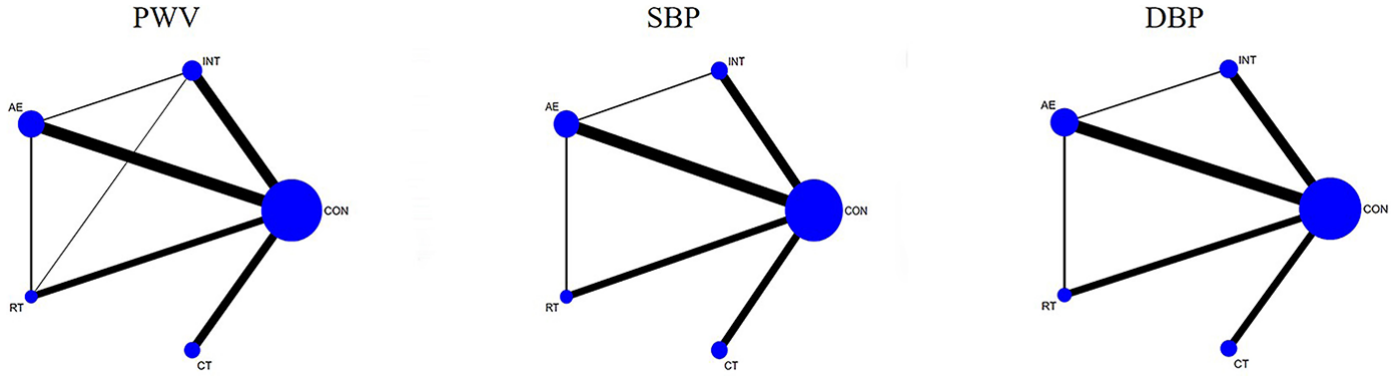
Network meta-analysis of eligible comparisons for pulse wave velocity, systolic blood pressure, and diastolic blood pressure.

The network evidence plot compares the differential impact of various exercise interventions on PWV and secondary outcomes. Figure 3 illustrates the NMA chart depicting PWV, SBP, and DBP. The connecting lines between nodes represent direct relationships between interventions, while the size of each node and the thickness of connecting lines are proportional to the number of studies conducted. As depicted in the figure, AE intervention studies are predominant, whereas RT intervention studies are relatively scarce.

The inconsistency test plots include the loop-special approach, node-splitting, and global inconsistency tests, which assess the consistency of PWV, SBP, and DBP at the loop level, local level, and global level, respectively (Supplementary Appendix 9). The results of the loop-special approach indicate that all closed loops involving PWV, SBP, and DBP exhibit good consistency except for INT-AE-RT, which showed inconsistency regarding PWV. Global inconsistency was assessed using an inconsistency model. The results of this model demonstrated that the *p*-values for PWV, SBP, and DBP were all greater than 0.05, indicating overall good consistency. Furthermore, the node-splitting analysis revealed no significant difference between indirect comparisons (between pairwise comparisons of each item) and direct comparisons (*p* > 0.05), suggesting reliable results (Supplementary Appendix 9).

The network forest plots illustrate the differences in intervention effects between various exercise types (including the CON) through pairwise comparisons. Supplementary Appendix 10 displays the network forest diagrams for PWV, SBP, and DBP with 95% confidence intervals and 95% prediction intervals.

The contribution of direct and indirect comparisons to NMA is illustrated in the network contribution graph (Supplementary Appendix 8), which also displays the number of studies for each direct comparison.

Funnel plots were used to assess publication bias in the NMA. PWV, SBP, and DBP all showed high symmetry, indicating an absence of publication bias.

SUCRA was employed to rank and compare the intervention effects of different types of exercise on PWV, SBP, and DBP. Supplementary Appendix 12 presents the SUCRA probability results for various exercise interventions.

#### 3.5.5 Pooled estimates of primary outcomes

The comparative effectiveness results of PWV are presented in Table 3. CT [SMD = −0.69, 95% CI (0.21 to 1.17), *p* < 0.0001] and INT [SMD = −0.50, 95% CI (0.07 to 0.93), *p* < 0.001] demonstrated significant improvements in PWV compared to the CON group; However, RT [SMD = −0.11, 95% CI (−0.36 to 0.59), *p* > 0.05] and AE [SMD = −0.33, 95% CI (−0.04 to 0.71), *p* > 0.05] did not show significant improvements in PWV compared to the CON group.

**Table 3:** Probability ranking plots of PWV and secondary outcomes.

PWV (m/s)				
**CT**	0.58 (−0.10 to 1.25)	0.35 (−0.26 to 0.97)	0.19 (−0.45 to 0.83)	**0.69 (0.21 to 1.17)**
−0.58 (−1.25 to 0.10)	**RT**	−0.22 (−0.79 to 0.34)	−0.39 (−1.00 to 0.23)	0.11 (−0.36 to 0.59)
−0.35 (−0.97 to 0.26)	0.22 (−0.34 to 0.79)	**AE**	−0.16 (−0.71 to 0.38)	0.33 (−0.04 to 0.71)
−0.19 (−0.83 to 0.45)	0.39 (−0.23 to 1.00)	0.16 (−0.38 to 0.71)	**INT**	**0.50 (0.07 to 0.93)**
**−0.69 (−1.17 to −0.21)**	−0.11 (−0.59 to 0.36)	−0.33 (−0.71 to 0.04)	**−0.50 (−0.93 to −0.07)**	**CON**
SBP (mm/Hg)				
**CT**	1.17 (−2.38 to 4.72)	−1.47 (−4.82 to 1.88)	−1.60 (−5.37 to 2.17)	2.22 (−0.23 to 4.67)
−1.17 (−4.72 to 2.38)	**RT**	−2.64 (−5.87 to 0.59)	−2.77 (−6.58 to 1.04)	1.05 (−1.48 to 3.59)
1.47 (−1.88 to 4.82)	2.64 (−0.59 to 5.87)	**AE**	−0.13 (−3.57 to 3.32)	**3.69 (1.36 to 6.02)**
1.60 (−2.17 to 5.37)	2.77 (−1.04 to 6.58)	0.13 (−3.32 to 3.57)	**INT**	**3.82 (0.94 to 6.70)**
−2.22 (−4.67 to 0.23)	−1.05 (−3.59 to 1.48)	**−3.69 (−6.02 to −1.36)**	**−3.82 (−6.70 to −0.94)**	**CON**
DBP (mm/Hg)				
**CT**	1.96 (−1.44 to 5.37)	0.52 (−2.57 to 3.62)	0.97 (−2.42 to 4.37)	2.03 (−0.38 to 4.43)
−1.96 (−5.37 to 1.44)	**RT**	−1.44 (−4.35 to 1.47)	−0.99 (−4.37 to 2.39)	0.06 (−2.34 to 2.46)
−0.52 (−3.62 to 2.57)	1.44 (−1.47 to 4.35)	**AE**	0.45 (−2.46 to 3.36)	1.50 (−0.46 to 3.47)
−0.97 (−4.37 to 2.42)	0.99 (−2.39 to 4.37)	−0.45 (−3.36 to 2.46)	**INT**	1.05 (−1.36 to 3.46)
−2.03 (−4.43 to 0.38)	−0.06 (−2.46 to 2.34)	−1.50 (−3.47 to 0.46)	−1.05 (−3.46 to 1.36)	**CON**

INT, interval training; AE, aerobic exercise; RT, resistance exercise; CT, combined training; CON, control group; PWV, pulse wave velocity; SBP, systolic blood pressure; DBP, diastolic blood pressure.

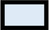
, efficacy (response rate); 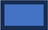, comparison; 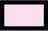, acceptability (dropout rate); 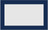, group.

Effects are expressed as the effect size (95% CI) between interventions. Bold indicates that the data are significant, light gray areas indicate the effect of the longitudinal versus the lateral intervention, white areas indicate the effect of the lateral versus the longitudinal intervention, dark gray areas represent the intervention category, and black areas represent the group. For example, “−0.69 (−1.17, −0.21)” (column 1, row 6) indicates that CT (longitudinal intervention) significantly reduces PWV compared with CON (transverse intervention).

The SUCRA probability ranking results displayed in Table 3 indicate that CT exhibited the highest efficacy in improving PWV with a SUCRA value of 87.2%, followed by INT with a SUCRA value of 73.1%, and AE with a SUCRA value of 54.2%. Conversely, RT had the lowest improvement effect with a SUCRA value of 9.3%.

#### 3.5.6 Pooled estimates of the secondary outcome

The secondary indicators of this study were mainly DBP and SBP. As shown in Table 3, AE [SMD = −3.69, 95% CI (1.36 to 6.02), *p* < 0.0001] and INT [SMD = −3.82, 95% CI (0.94 to 6.70), *p* < 0.001] significantly improved PWV compared with the CON group. However, RT [SMD = −1.05, 95% CI (−1.48 to 3.59), *p* > 0.05] and CT [SMD = −2.22, 95% CI (−0.23 to 4.67), *p* > 0.05] may not significantly improve SBP compared with the CON group. DBP was not significantly improved by INT, AE, RT, and CT (*p* > 0). The results of the SUCRA probability ranking in Table 4 show that INT (SUCRA = 81.3) has the best effect on improving SBP among the four exercise interventions: INT, AE, RT, and CT. RT had the least impact on improving SBP (SUCRA = 29.7), CT (SUCRA = 78.8) had the most impact on improving DBP, and RT (SUCRA = 27.4) had the least impact on improving DBP (SUCRA = 29.7).

**Table 4:** Ranking of exercise interventions in order of effectiveness.

Treatment	SUCRA	PR best (%)	Mean rank
Pulse wave velocity (49 studies, *N* = 2,034)			
CT	87.2	63.9	1.5
INT	73.1	27.5	2.1
AE	54.2	7.2	2.8
RT	26.3	1.4	3.9
CON	9.3	0	4.6
Systolic blood pressure (46 studies, *N* = 1,802)			
INT	81.3	48.7	1.7
AE	80.4	41.1	1.8
CT	52.5	8.8	2.9
RT	29.7	1.4	3.8
CON	6	0	4.8
Diastolic blood pressure (42 studies, *N* = 1,643)			
CT	78.8	51.3	1.8
AE	69.2	26.6	2.2
INT	54.9	17.8	2.8
RT	27.4	4.2	3.9
CON	19.6	0.1	4.2

INT, interval training; AE, aerobic exercise; RT, resistance exercise; CT, combined training; CON, control group.

#### 3.5.7 Subgroup NMA of the primary outcomes

Because the subjects in this study are persons at risk for cardiovascular disease, which is usually associated with multiple complications such as obesity, hypertension, diabetes, metabolic syndrome, etc., it was challenging to control confounding factors like age and gender within subgroups when analyzing the study results. However, subgroup analysis of exercise intensity was feasible, and we performed subgroup analyses of exercise intensity (Supplementary Appendix 15, 16).

Subgroup analysis of exercise intensity showed that CT [SMD = −0.67, 95% CI (0.07 to 1.28), *p* < 0.05] significantly improved PWV in the moderate-intensity subgroup (Supplementary Appendix 15.10) and RT [SMD = −0.40, 95% CI (0.12 to 0.68), *p* < 0.05] and INT [SMD = −0.40, 95% CI (0.16 to 0.64), *p* < 0.05] can significantly improve PWV in the high-intensity subgroup (Supplementary Appendix 16.10). The results of the SUCRA probability ranking showed that INT (SUCR = 83.3) was the most likely to be the best exercise intervention for PWV in the moderate-exercise-intensity subgroup, while RT (SUCRA = 29.1) was the worst (Supplementary Appendix 15.9). In the high-exercise intensity subgroup, CT (SUCRA = 85.7) may be the most effective exercise intervention for PWV, while AE (SUCRA = 29.1) may be the least effective exercise intervention (Supplementary Appendix 16.9). In conclusion, exercise intensity may be an important factor affecting the effect of exercise intervention in PWV.

#### 3.5.8 GRADE assessment

Table 4 presents the GRADE evaluation results for PWV, showing a high level of performance with most comparisons achieving medium to high confidence. Supplementary measures, SBP and DBP, were also evaluated using the GRADE framework (Supplementary Appendix 14). This showed lower to moderate confidence for most SBP comparisons and moderate to high confidence for most DBP comparisons. Overall, PWV had the highest confidence in the GRADE assessment, while SBP had relatively low confidence.

## 4 Discussion

### 4.1 Primary outcome

**Figure 4:**
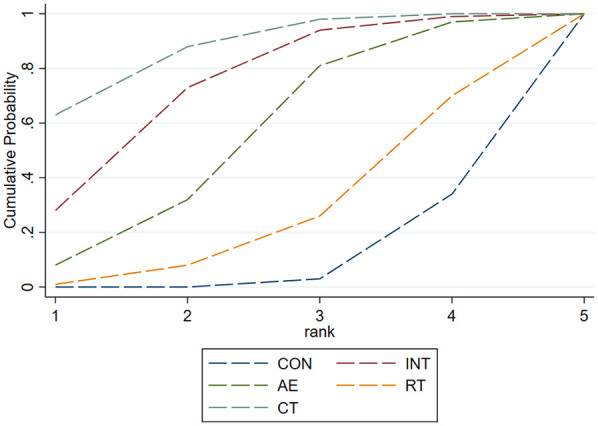
Cumulative ranking probability plot of each intervention on PWV.

This study examined the impact of four exercise interventions (INT, AE, RT, and CT) on PWV, SBP, and DBP in individuals at high risk of CVD. The network meta-analysis, including 43 RCTs with 2,034 participants, found that CT and INT significantly reduced PWV, whereas RT and AE did not. As shown in Figure 4, SUCRA analysis ranked CT as the most effective, followed by INT. AE and RT were less effective. Subgroup analysis showed that moderate-intensity INT was most effective for AS, while high-intensity CT outperformed AE in improving AS.

In studies involving individuals at high risk of CVD, Montero et al. (10) examined the effects of AE on arterial AS in subjects with prehypertension and hypertension. Their results showed that AE, particularly when combined with a reduction in SBP below the median or extended duration, positively affected AS in patients with prehypertension or hypertension.

Montero et al. (9) also studied the impact of AE on AS in obese individuals, finding no significant improvement in AS among middle-aged and older obese adults undergoing AE. However, subgroup and meta-regression analyses suggested that low-intensity AE combined with a reduction in DBP could reduce AS in obese individuals.

Marzolini et al. (7) compared the effects of CT (AE + RT) to AE alone in patients with coronary heart disease. They found that AE + RT led to superior improvements in AS and was more effective than AE alone in enhancing body composition, strength, and certain cardiovascular health indicators.

Evans et al. (11) investigated the effects of RT on AS in high-risk CVD populations. Their study showed that RT did not worsen AS or the blood system, despite increasing sympathetic norepinephrine levels, which may lead to vasoconstriction and higher blood pressure. In fact, RT could be more effective than AE in improving cardiovascular health-related conditions.

Consistent with previous research, this study found that both a CT program (combining AE and RT) (SMD = −0.69, *p* < 0.001) and an INT program (SMD = −0.50, *p* < 0.001) effectively reduced PWV in individuals at high risk for CVD. Given that an effect size of 0.5 or greater suggests practical relevance (62), both CT and INT are recommended as effective interventions for improving AS in high-risk CVD populations.

In contrast to previous findings, AE (SMD = −0.33, *p* > 0.01) did not significantly reduce PWV in high-risk populations despite its known benefits for AS in healthy individuals. AE's positive effects in healthy people are attributed to mechanisms like vascular remodeling, enhanced endothelial function, and reduced oxidative stress (9). However, AE did not significantly lower AS in individuals with hypertension, obesity, or elderly individuals who are at high risk for CVD. Effective AE for high-risk populations typically involves lowering systolic blood pressure below the median, extending the intervention duration, or adjusting exercise intensity. Therefore, AE is not recommended as the primary intervention for improving AS in high-risk CVD populations.

The impact of RT on AS remains debated. Some studies suggest that high-intensity RT may increase blood pressure and reduce AS, while others find no effect (6, 10). Our study also showed that RT (SMD = −0.11, *p* > 0.01) did not significantly reduce PWV. However, the subgroup analysis revealed that high-intensity RT effectively decreased PWV (SMD = −0.40, *p* < 0.001), suggesting its potential benefit for improving AS in high-risk individuals. Safety concerns related to high-intensity RT, including potential increases in blood pressure and acute cardiac strain, limit its clinical recommendation for improving AS.

The biological mechanisms behind RT's effects on AS vary by health status. In young, healthy individuals, high-intensity RT can stimulate the sympathetic nervous increased AS (73). In contrast, individuals at high risk for CVD, such as those with metabolic syndrome or diabetes, may experience decreased hormone secretion that mitigates these adverse effects. In addition, RT's impact on arterial structure and load-bearing characteristics can positively influence AS.

Finally, high-intensity CT (SMD = −0.71) was more effective than moderate-intensity CT (SMD = −0.67) in improving AS in high-risk CVD populations. Previous studies have suggested CT is the best intervention for improving AS in general populations. However, the sequence of AE and RT in CT is important; CT with AE following RT but not before RT can reduce AS (8). This study included CT with both AE and RT, but the sequence was not consistently specified. Further research is needed to determine the optimal order of exercises in CT programs.

### 4.2 Secondary outcomes

The secondary findings of this study evaluated the impact of four exercise interventions (INT, AE, RT, and CT) on blood pressure, including SBP and DBP. Analysis of SBP showed that INT (SMD = −0.382, *p* < 0.001) and AE (SMD = −0.369, *p* < 0.001) significantly reduced SBP in high-risk CVD populations, while CT (SMD = −2.22, *p* > 0.01) and RT (SMD = −1.05, *p* > 0.01) did not. SUCRA ranking indicated that INT had the highest effect (SUCRA = 81.3), followed by AE (SUCRA = 80.4) and CT (SUCRA = 52.5), with RT scoring the lowest (SUCRA = 29.7).

For DBP, none of the interventions showed significant reductions. SUCRA analysis revealed that CT had the best effect (SUCRA = 78.8), followed by AE (SUCRA = 69.2) and INT (SUCRA = 54.9), while RT had the lowest effect (SUCRA = 27.4).

These results align with previous research confirming AE and INT's benefits on SBP. Some studies suggest that high-intensity INT might be more effective than AE for individuals with cardiopulmonary issues or advanced age due to shorter exercise and recovery times. The impact of RT on blood pressure remains debated, with mixed results in prior studies (74). This study found that neither RT nor CT significantly improved blood pressure.

The lack of significant effects on DBP across all interventions may be due to individual variability in high-risk CVD populations. Normal blood pressure values are 130/80 mmHg, and DBP typically should not exceed 90 mmHg (75). Although AE, RT, and CT have been shown to improve various cardiovascular metrics, age-related changes in blood vessels, such as elastin fiber degeneration and increased collagen, may obscure the immediate benefits on DBP for high-risk groups like the elderly, obese, or those with heart disease.

### 4.3 Subgroup NMA of the primary outcome

This study examined how exercise intensity affects PWV. The results showed that in the moderate-intensity group, CT significantly improved PWV [SMD = −0.67, 95% CI (0.07 to 1.28), *p* < 0.05; see Supplementary Appendix 15.10]. In the high-intensity group, both RT [SMD = 0.40, 95% CI (0.12 to 0.68), *p* < 0.05] and HIIT [SMD = 0.40, 95% CI (0.16 to 0.64), *p* < 0.05] were effective in improving PWV (see Supplementary Appendix 16.10).

SUCRA rankings showed that for moderate-intensity exercise, INT (SUCRA = 83.3) was the most effective, while RT (SUCRA = 21.6) was the least effective (see Supplementary Appendix 15.9). For high-intensity exercise, CT (SUCRA = 85.7) was the most effective, and AE (SUCRA = 29.5) was the least effective (see Supplementary Appendix 16.9).

These findings underscore the importance of exercise intensity in determining the effectiveness of interventions on PWV. Previous research shows varied effects of INT, AR, RT, and CT based on intensity. For instance, Way et al. (12) found that high-intensity aerobic exercise was more effective for improving AS in healthy adults than moderate-intensity exercise. On the other hand, Montero et al. (9) reported that low-intensity aerobic exercise improved AS in obese individuals, while moderate to high-intensity aerobic exercise did not.

In summary, higher-intensity CT and RT may be more effective for improving PWV, offering valuable guidance for choosing suitable exercise intensities for individuals at high cardiovascular risk.

### 4.4 Strengths and limitations

This study utilized a network meta-analysis, allowing us to compare multiple intervention modalities simultaneously. This approach provides a broader research perspective and significantly enhances the study's value. We focused on high-risk CVD populations, an area that has been underexplored in previous research. Since AS is a common complication of CVD, examining this group is crucial for validating the preventive and therapeutic benefits of exercise on AS.

Our findings are notable and diverge from previous research. We recommend CT and INT as preferred exercise methods for improving arterial stiffness in high-risk CVD populations. In addition, moderate-intensity AE and supervised high-intensity RT are also recommended for these individuals.

However, this study has certain limitations. First, we only included INT, AE, RT, and CT as common interventions, excluding other forms like mental and physical exercises, stretching, and pharmacological treatments, which have limited data. Second, while we focused on PWV, SBP, and DBP as key indicators of AS, other measures such as augmentation index, blood lipids, and blood sugar were not included due to validity concerns. Third, insufficient sample size is also a significant limitation when conducting subgroup analyses on exercise intensity. In addition, despite various analyses, the heterogeneity in CT remains unresolved and is acknowledged as a limitation in this study. Future research with larger sample sizes should address these limitations and explore additional interventions. Potential limitations in future studies may include variability in study populations, differences in study designs, and the influence of unmeasured confounders.

## 5 Conclusion

This systematic review and network meta-analysis provides strong evidence that both CT and INT significantly reduce PWV in high-risk CVD populations. In addition, CT and AE effectively lower SBP. SUCRA rankings indicate that CT is the most effective for reducing PWV, while INT is best for lowering SBP. RT is the least effective for reducing PWV, SBP, and DBP. Subgroup analysis by exercise intensity shows that moderate-intensity INT has the greatest impact on AS. Conversely, high-intensity CT contributes to greater improvements in AS compared to AE at a similar intensity. However, these results should be interpreted with caution due to substantial heterogeneity in the CT studies. The variation in CT findings may influence the overall conclusions, and further studies with larger sample sizes and more consistent methodologies are needed to confirm these findings.

Based on the available evidence, individuals at high risk of CVD should consider supervised moderate-intensity AE and high-intensity RT, with INT being the preferred method for effectively improving arterial stiffness.

## Data Availability

The original contributions presented in the study are included in the article/Supplementary Material, further inquiries can be directed to the corresponding authors.
