# Supplementary material for: Comparative effectiveness of exercise interventions on arterial stiffness in individuals at risk for cardiovascular disease: a systematic review and network meta-analysis
Source: Front Cardiovasc Med. 2025 Feb 27;12:1489382. doi: 10.3389/fcvm.2025.1489382 (PMC11905979; doi:10.3389/fcvm.2025.1489382)
Supplement: Supplementary file 1 [file Datasheet1.docx]

| **Supplemental materials** | **Page** |
| --- | --- |
| **Appendix 1.** Completed PRISMA-NMA checklist. | 2-8 |
| **Appendix 2.** Search strategy of PubMed, Embase, Cochrane, Web of Science, and EBSCO. | 9-12 |
| **Appendix 3.** Transformation formulas and the classifications of exercise interventions. | 13-16 |
| **Appendix 4.** Characteristics of the included studies. | 17-22 |
| **Appendix 5.** Risk of bias assessment. | 23-26 |
| **Appendix 6.** Funnel plot of Pulse Wave Velocity, Systolic Blood Pressure, and Diastolic Blood Pressure in pairwise meta-analysis. | 27-29 |
| **Appendix 7.** Forest plots for Pulse Wave Velocity, Systolic Blood Pressure, and Diastolic Blood Pressure. | 30-35 |
| **Appendix 8.** Contributions of direct and indirect comparisons to NMA and the number of studies of each direct comparison of Pulse Wave Velocity, Systolic Blood Pressure, and Diastolic Blood Pressure. | 36-38 |
| **Appendix 9.** Inconsistency of Pulse Wave Velocity, Systolic Blood Pressure, and Diastolic Blood Pressure tested by loop-specific heterogeneity estimates, inconsistency model and node splitting analysis. | 39-43 |
| **Appendix 10.** Forest plots of eligible comparisons of Pulse Wave Velocity, Systolic Blood Pressure, and Diastolic Blood Pressure. | 44-46 |
| **Appendix 11.** The funnel plot graphics of Pulse Wave Velocity, Systolic Blood Pressure, and Diastolic Blood Pressure in NMA. | 47-49 |
| **Appendix 12.** Area under the curve for cumulative ranking probability of each intervention on Pulse Wave Velocity, Systolic Blood Pressure, and Diastolic Blood Pressure. | 50-53 |
| **Appendix 13.**  Network of eligible comparisons for Pulse Wave Velocity, Systolic Blood Pressure, and Diastolic Blood Pressure. | 54-57 |
| **Appendix 14.** GRADE for Pulse Wave Velocity, Systolic Blood Pressure, and Diastolic Blood Pressure.  **Appendix 15.** Subgroup analysis for pulse wave velocity in moderate intensity group.  **Appendix 16.** Subgroup analysis for pulse wave velocity in high intensity group.  **Appendix 17.** List of included studies. | 58-66 |
|  |  |
|  | 67-76 |
|  | 77-85 |
|  | 86-92 |

## Appendix 1. PRISMA NMA checklist of items to include when reporting a systematic review involving a network meta-analysis.

| **Section/Topic** | **Item #** | | **Checklist Item** | | | **Reported on Page #** |  |
| --- | --- | --- | --- | --- | --- | --- | --- |
| **TITLE** |  | |  | | |  |  |
| Title | 1 | | Identify the report as a systematic review incorporating a network meta-analysis (or related form of meta-analysis). | | | 1 |  |
| **ABSTRACT** |  | |  | | |  |  |
| Structured summary | 2 | | Provide a structured summary including, as applicable:  **Background:** main objectives  **Methods:** data sources; study eligibility criteria, participants, and interventions; study appraisal; and *synthesis methods, such as network meta-analysis.*  **Results:** number of studies and participants identified; summary estimates with corresponding confidence/credible intervals; treatment rankings may also be discussed. Authors may choose to summarize pairwise comparisons against a chosen treatment included in their analyses for brevity.  **Discussion/Conclusions:** limitations; conclusions and implications of findings.  **Other:** primary source of funding; systematic review registration number with registry name. | | | 2 |  |
| **INTRODUCTION** | | | |  |  |  |  |
| Rationale | 3 | | Describe the rationale for the review in the context of what is already known*, including mention of why a network meta-analysis has been conducted.* | | | 4-6 |  |
| Objectives | 4 | | Provide an explicit statement of questions being addressed, with reference to participants, interventions, comparisons, outcomes, and study design (PICOS). | | | 4-6 |  |
| **METHODS** |  | |  | | |  |  |
| Protocol and registration | 5 | | Indicate whether a review protocol exists and if and where it can be accessed (e.g., Web address); and, if available, provide registration information, including registration number. | | | 7 |  |
| Eligibility criteria | 6 | | Specify study characteristics (e.g., PICOS, length of follow-up) and report characteristics (e.g., years considered, language, publication status) used as criteria for eligibility, giving rationale. Clearly describe eligible treatments included in the treatment network and note whether any have been clustered or merged into the same node (with justification) | | | 7-8 |  |
| Information sources | 7 | | Describe all information sources (e.g., databases with dates of coverage, contact with study authors to identify additional studies) in the search and date last searched. | | | 7-8 |  |
| Search | 8 | | Present full electronic search strategy for at least one database, including any limits used, such that it could be repeated. | | | Appendix 2 |  |
| Study selection | 9 | | State the process for selecting studies (i.e., screening, eligibility, included in systematic review, and, if applicable, included in the meta-analysis). | | | 7-8 |  |
| Data collection process | 10 | | Describe method of data extraction from reports (e.g., piloted forms, independently, in duplicate) and any processes for obtaining and confirming data from investigators. | | | 8-9 |  |
| Data items | 11 | | List and define all variables for which data were sought (e.g., PICOS, funding sources) and any assumptions and simplifications made. | | | 8 |  |
| **Geometry of**  **the network** | **S1** | | Describe methods used to explore the geometry of the treatment network under study and potential biases related to it. This should include how the evidence base has been graphically summarized for presentation, and what characteristics were compiled and used to describe the evidence base to readers. | | | 9 |  |
| Risk of bias within individual studies | 12 | | Describe methods used for assessing risk of bias of individual studies (including specification of whether this was done at the study or outcome level), and how this information is to be used in any data synthesis. | | | 9 |  |
| Summary measures | 13 | | State the principal summary measures (e.g., risk ratio, difference in means). Also describe the use of additional summary measures assessed, such as treatment rankings and surface under the cumulative ranking curve (SUCRA) values, as well as modified approaches used to present summary findings from meta-analyses. | | | 9-11 |  |
| Planned methods of analysis | 14 | | Describe the methods of handling data and combining results of studies for each network meta-analysis. This should include, but not be limited to:   - Handling of multi-arm trials; - Selection of variance structure; - Selection of prior distributions in Bayesian analyses; and - Assessment of model fit. | | | 9-11 |  |
| **Assessment of Inconsistency** | **S2** | | Describe the statistical methods used to evaluate the agreement of direct and indirect evidence in the treatment network(s) studied. Describe efforts taken to address its presence when found. | | | 10-11 |  |
| Risk of bias across studies | 15 | | Specify any assessment of risk of bias that may affect the cumulative evidence (e.g., publication bias, selective reporting within studies). | | | 9 |  |
| Additional analyses | 16 | | Describe methods of additional analyses if done, indicating which were pre-specified. This may include, but not be limited to, the following:   - Sensitivity or subgroup analyses; - Meta-regression analyses; - Alternative formulations of the treatment network; and - Use of alternative prior distributions for Bayesian analyses (if applicable). | | | 9-11 |  |
| **RESULTS†** |  | |  | | |  |  |
| Study selection | 17 | | Give numbers of studies screened, assessed for eligibility, and included in the review, with reasons for exclusions at each stage, ideally with a flow diagram. | | | 12 |  |
| **Presentation**  **of network structure** | **S3** | | Provide a network graph of the included studies to enable visualization of the geometry of the treatment network. | | | Figure 1 |  |
| **Summary of network geometry** | **S4** | | Provide a brief overview of characteristics of the treatment network. This may include commentary on the abundance of trials and randomized patients for the different interventions and pairwise comparisons in the network, gaps of evidence in the treatment network, and potential biases reflected by the network structure. | | | 12-13 |  |
| Study characteristics | 18 | | For each study, present characteristics for which data were extracted (e.g., study size, PICOS, follow-up period) and provide the citations. | | | Appendix 17 | |
| Risk of bias within studies | 19 | | Present data on risk of bias of each study and, if available, any outcome level assessment. | | | Appendix 5 |  |
| Results of individual studies | 20 | | For all outcomes considered (benefits or harms), present, for each study: 1) simple summary data for each intervention group, and 2) effect estimates and confidence intervals. Modified approaches may be needed to deal with information from larger networks. | | | 13-14 |  |
| Synthesis of results | 21 | | Present results of each meta-analysis done, including confidence/credible intervals. In larger networks, authors may focus on comparisons versus a particular comparator (e.g., placebo or standard care), with full findings presented in an appendix. League tables and forest plots may be considered to summarize pairwise comparisons. If additional summary measures were explored (such as treatment rankings), these should also be presented. | | | 14-17,  Table 3 |  |
| **Exploration for inconsistency** | **S5** | | Describe results from investigations of inconsistency. This may include such information as measures of model fit to compare consistency and inconsistency models, *P* values from statistical tests, or summary of inconsistency estimates from different parts of the treatment network. | | | 16,  Appendix 9 |  |
| Risk of bias across studies | 22 | | Present results of any assessment of risk of bias across studies for the evidence base being studied. | | | 17 |  |
| Results of additional analyses | 23 | | Give results of additional analyses, if done (e.g., sensitivity or subgroup analyses, meta-regression analyses, alternative network geometries studied, alternative of prior distributions for Bayesian analyses, and so forth). | | | 16-17  Appendix 15 | |
| **DISCUSSION** | |  |  | | |  |  |
| Summary of evidence | 24 | | Summarize the main findings, including the strength of evidence for each main outcome; consider their relevance to key groups (e.g., healthcare providers, users, and policymakers). | | | 18-24 |  |
| Limitations | 25 | | Discuss limitations at study and outcome level (e.g., risk of bias), and at review level (e.g., incomplete retrieval of identified research, reporting bias). Comment on the validity of the assumptions, such as transitivity and consistency. Comment on any concerns regarding network geometry (e.g., avoidance of certain comparisons). | | | 24 |  |
| Conclusions | 26 | | Provide a general interpretation of the results in the context of other evidence, and implications for future research. | | | 24-25 |  |
| **FUNDING** |  | |  | | |  |  |
| Funding | 27 | | Describe sources of funding for the systematic review and other support (e.g., supply of data); role of funders for the systematic review. This should also include information regarding whether funding has been received from manufacturers of treatments in the network and/or whether some of the authors are content experts with professional conflicts of interest that could affect use of treatments in the network. | | | Not Applicable |  |

PICOS = population, intervention, comparators, outcomes, study design.

* Text in italics indicate wording specific to reporting of network meta-analyses that has been added to guidance from the PRISMA statement.

† Authors may wish to plan for use of appendices to present all relevant information in full detail for items in this section.

## Appendix 2. Search strategy of PubMed, Embase, Cochrane, Web of Science, and EBSCO.

**Appendix 2.1** Search strategy in PubMed(*N*=68)

| Step | Search strategy | Results |
| --- | --- | --- |
| #1 | ((((((((((physical activity[Title/Abstract]) OR (training[Title/Abstract])) OR (aerobic exercise[Title/Abstract])) OR (aerobic training[Title/Abstract])) OR (moderate intensity continuous training[Title/Abstract])) OR (resistance training[Title/Abstract])) OR (strength training[Title/Abstract])) OR (combined training[Title/Abstract])) OR (sprint interval training[Title/Abstract])) OR (high intensity interval training[Title/Abstract])) OR ("Exercise"[Mesh])))))))))) | 820682 |
| #2 | (((arterial stiffness [Title/Abstract])) OR (PWV[Title/Abstract])) OR (pulse wave velocity [Title/Abstract]))) | 17685 |
| #3 | (((randomized controlled trial [Title/Abstract]) OR (randomized [Title/Abstract])) OR (placebo [Title/Abstract])) OR (RCT[Title/Abstract]))) | 795131 |
| #4 | ((control group [Title/Abstract]) OR (control [Title/Abstract])) OR (active control [Title/Abstract])) | 3081609 |
| #5 | (((((Hypertension [Title/Abstract]) OR (Hypercholesterolemia [Title/Abstract])) OR (obesity [Title/Abstract])) OR (Type 2 diabetes [Title/Abstract])) OR (metabolic syndrome [Title/Abstract])) OR (Older persons over 60 years [Title/Abstract])))) | 928650 |
| #6 | #1 AND #2 AND #3 AND #4 AND #5 | 68 |

**Appendix 2.2** Cochrane search results (*N*=441)

| Step | | Search strategy | Results |
| --- | --- | --- | --- |
| #1 | | MeSH descriptor: [Exercise] explode al trees | 32970 |
| #2 | | (training): ti, ab, KW or (aerobic exercise): ti, ab, KW or (aerobic training): ti, ab, KW or (resistance training): ti, ab, KW or (strength training): ti, ab, KW or (moderate intensity continuous Limits training): ti, ab, KW or (combined training): ti, ab, KW or (sprint interval training): ti, ab, KW or (high intensity interval training): ti, ab, KW or (physical activity): ti, ab, KW | 160382 |
| #3 | | #1 OR #2 | 171772 |
| #4 | | (arterial stiffness) ti, ab, kw or (PWV):t, ab, kw or (pulse wave velocity) t, ab, kw | 5988 |
| #5 | | (randomized) ti, ab, kw or (randomized controlled trial) ti, ab, kw or (placebo): ti, ab, kw or (RCT): ti, ab, kw | 1179451 |
| #6 | (control group): ti, ab, kw or (control): ti, ab, kw or (active control): ti, ab, kw | 581557 |  |
| #7 | | #3 and #4 and#5 and #6 | 441 |

**Appendix 2.3** Embase search results (*N*=241)

| Step | | Search strategy | Results |
| --- | --- | --- | --- |
| #1 | | 'exercise'/exp | 429601 |
| #2 | | "training': ti, ab, kw OR 'aerobic ‘exercise': ti, ab, kw OR 'aerobic training': ti, ab, kw OR 'resistance training':ti, ab, kw OR 'strength training':ti, ab, kw OR 'moderate intensity continuous training':ti, ab, kw OR 'combined training':ti, ab, kw OR 'sprint interval training':ti, ab, kw OR 'high intensity interval training'. kw OR 'strength training’: ti, ab, kw OR 'moderate intensity continuous training’: ti, ab, kw OR 'combined training':ti, ab, kw OR 'sprint interval training':ti, ab, kw OR 'high intensity interval training'.:ti, ab, kw OR 'physical activity'.:ti, ab, kw | 901921 |
| #3 | | #1 OR #2 | 1187115 |
| #4 | | 'Arterial stiffness’: ti, ab, kw OR 'PWV’: ti, ab, kw OR 'pulse wave velocity’: ti, ab, kw | 31655 |
| #5 | | 'randomized': ti, ab, kw OR 'randomized controlled trial': ti, ab, kw OR 'placebo': ti, ab, kw OR 'rct': ti, ab, kw | 1129810 |
| #6 | 'Control group': ti, ab, kw OR 'control': ti, ab, kw OR 'active control': ti, ab, kw | 4002500 |  |
| #7 | | #3 and #4 and#5 and #6 | 241 |

**Appendix 2.4** EBSCO search results (*N*=36)

| Step | Search strategy | Results |
| --- | --- | --- |
| #1 | AB physical activity OR AB training OR AB aerobic exercise OR AB aerobic training OR AB moderate intensity continuous training OR AB resistance training OR AB strength training OR AB combined training OR AB sprint interval | 1593159 |
| #2 | D AB arterial stiffness OR AB pulse wave velocity OR AB PWV | 14444 |
| #3 | AB randomized controlled trial OR AB randomized OR AB placebo OR AB rct | 1023801 |
| #4 | AB hypertension OR AB old people OR AB obesity OR AB type 2 diabetes OR AB metabolic syndrome OR AB postmenopausal women | 5382327 |
| #5 | a (AB hypertension OR AB chronic kidney disease OR AB obesity OR AB type 2 diabetes OR AB metabolic syndrome OR AB postmenopausal women) AND (S1 AND S2 AND S3 AND S4 AND5) | 1034164 |
| #6 | (AB hypertension OR AB chronic kidney disease OR AB obesity OR AB type 2 diabetes OR AB metabolic syndrome OR AB postmenopausal women) AND (S1 AND S2 AND S3 AND S4 AND S5) | 36 |

##

## Appendix 3. Transformation formulas and the classifications of exercise interventions.

**Appendix 3.1** Transformation formulas for estimating the mean and standard deviation.

(1) When calculating SD from M (confidence interval) for intervention or control group

a. the sample size in each group＞100

• SE= (upper limit-lower limit)/3.92

• SD=SE*N^ (1/2)

b. the sample size in each group≤100

• Input “tinv (1-0.95 , N_1_-1)” in Microsoft Excel to obtain t

• SE= (upper limit-lower limit)/ t

• SD=SE*N^(1/2)

(2) When calculating SD from MD and P value between intervention or control group

a. reporting the exact p value

• Input “tinv (p , N_1_+N_2_-2)” in Microsoft Excel to obtain t

• SE=MD/t

• SD1=SE/N_1_^(1/2)

b. reporting only significant levels (e.g. P＜0.05 or P＞0.05)

• The conservative calculation is to take the upper bound P. (e.g. P＜0.05 is replaced 0.05)

(3) When calculating M (SD) from the m (interquartile range) for intervention or control group

a. the sample size＞25, M=m;

the sample size≤25, M=(𝑎+2m+𝑏)/4

b. the sample size≤15, SD=(((𝑎−2𝑚+𝑏)^2^/4+(𝑏−𝑎)^2^)/12) ^ (1/2)

the sample size 15<n≤70, SD=(𝑏−𝑎)/4

the sample size＞70 , SD=(𝑏−𝑎)/6

(4) When calculating M (SD) from the m (interquartile range) for intervention or control group

a. M= (𝑞_1_ +𝑚+𝑞_3_)/3

b. calculation of SD

• Z=(0.25𝑁_1_+0.375)/(𝑁1+0.25)

• Input “norm. inv(1-z ,0 ,1)” obtain μ

• SD=(𝑞_3_−𝑞_1_)/(2∗𝜇)

Note:

M= mean; SE=standard error of mean; SD=standard deviation; N=sample size; MD= mean difference; m=median; a= minimum value; b = maximum value; q1= first quartile; q3= third quartile

**Appendix 3.2** The classifications of exercise interventions

To compare the effects of different types of exercise , we classified exercise interventions into the following broad categories: interval training (INT , any intermittent traditional mode of interval training only , including single-component MIIT and HIIT such as walking , running , cycling , rowing , swimming , elliptical exercise , stepping exercise) , aerobic exercise (AE , aiming to improve cardiovascular fitness including walking , running , or cycling) , resistance exercise (RE , with the intent of increasing muscular strength and power using elastic bands , weight-machines) , combined training (CT , A combination of CET and RT). Different types of exercise were further classified and encoded according to the frequency, intensity, duration per session, and the length of intervention (see table 3.1).

Appendix 3.2.1 The classifications for exercise according to the frequency, intensity, duration per session, and the length of intervention.

| Frequency | |
| --- | --- |
| Low | 1-2 times/week |
| Moderate | 3-4 times/week |
| High | 3≥5 times/week |
| Duration per session |  |
| Short | <30 minutes |
| Moderate-1 | 30-44 minutes |
| Moderate-2 | 45-59 minutes |
| long | ≥60 minutes |
| Length of intervention |  |
| Short | <13 weeks (3 months) |
| Moderate | 14-25 weeks (3 months-6 months) |
| long | ≥60 minutes (≥6 months) |
| Intensity | |
| Low | Interval training:  < 75%HR max/ 65% V̇O2max/65% HRR  Aerobic exercise: < 60%HR max/ 60% V̇O_2_max  Resistance training: < 50% of 1 RM |
| Moderate | Interval training:  75%-90% HR max/65–85% V̇O2max/65–85% HRR  Aerobic exercise: 60%-85% HR max/60–80% V̇O_2_max  Resistance training: 60–80% of 1 RM |
| High | Interval training:  >90% HR max/85% V̇O2max/85% V̇O2max  Aerobic exercise: >85% HR max/80% V̇O_2_max ,  Resistance training: 80–100% of 1 RM |

References: (Jarstad & Mamen , 2019; Morris et al. , 2017; Ramirez-Velez et al. , 2017; Zhou et al. , 2019)

## Appendix 4. Characteristics of the included studies.

| Study | Country | Characteristics of subject | | | | Interventions information | | | | | | |  |
| --- | --- | --- | --- | --- | --- | --- | --- | --- | --- | --- | --- | --- | --- |
|  |  | Disease/  condition | | Sample size (F) | age  (mean [SD]) | PWV  Measure | Type | Duration | Supervised | | | Comparator | Outcomes |
| Guimarães  2010^[1]^ | Brazil | Hypertension | | 65(42) | 50(7.2) | Complior | INT: treadmill with intensity alternating between 50% (2 min) and 80% (1 min) of HRR | 40min/session,  2session/week,  16weeks | | YES | No exercise | | ②, ⑤, ⑥ |
| Bouaziz 2019^[2]^ | France | Sedentary aged 70 or over | | 60(44) | 73.6(3.1) | SphygmoCor. | INT: 6*(4min at first ventilatory threshold (VT1) intensity + 1min at 40% of VT1 cycling exercise) | 30min/session,3session/week,9.5weeks | | YES | No exercise | | ②,⑤, ⑥ |
| Aghaei 2019^[3]^ | Iran | Stage 1 Hypertension | | 30 | 48(3.2) | Enverdis | INT: 4 reps of 4min activity at 75% to 90% of Vo2peak interspersed with 4-min passive/active (15%-30% of VO 2peak) recovery. | 32min/session,2session/week,8weeks | | YES | No exercise | | ①, ⑤, ⑥ |
| Zhuang  2023^[4]^ | China | Older persons | | 82(49) | 70(5.45) | Sphygmocor | INT: 3 daily cycling sessions of 40 mins 80% of HRmax | 40min/day, 3days/week, 12 weeks | | NO | No exercise | | ④, ⑤ |
| Rodriguez  2017^[5]^ | Spain | Metabolic syndrome | | 46 | 53.5(8.9) | Sphygmocor | INT: four bouts of 4 min of pedaling at an intensity that elicited 90% of maxHR interspersed with 3-min active re-covery periods at 70% maxHR | 45min/session,3session/week,24weeks | | YES | No exercise | | ②, ⑤, ⑥ |
| Deiseroth 2019^[6]^ | Switzerland | Older persons | | 68 | 59(7.5) | Sphygmocor | INT: 4 × 4 min at 80–90% of HRmax with 3 min active recovery at an intensity of 60–70% HRmax. | 40min/session,3session/week, 12weeks | | YES | No exercise | | ③, ⑤, ⑥ |
| Zhang 2022^[7]^ | China | T2D | | 77(38) | 57(7) | Complior | INT: 3 daily cycling sessions of 40 mins 70-90% of HRR | 40min/day, 3days/week,  52 weeks | | YES | No exercise | | ②, ⑤, ⑥ |
| Kim 2017^[8]^ | USA | Older persons | | 49(33) | 65.2(1.3) | Sphygmocor | INT: 4*4 min at 90% HRpeak alternated by 3*3 min active recovery at 70% HRpeak. | 40min/day,  3days/week,  12 weeks | | YES | No exercise | | ②, ⑤, ⑥ |
| Bellia  2017^[9]^ | Italy | Overweight T2D | | 22(6) | 57.4(7.3) | Sphygmocor | INT: 2-4 rep of 4 minutes’ walk to 75-80% of the HRmax with active 3-minute recovery to 45-50% of the HRmax. | 40min/day,  3days/week,  12 weeks | | YES | Usual care | | ①, ⑤, ⑥ |
| Way 2020^[10]^ | Australia | Overweight T2D | | 34(15) | 55.1(1.4) | Sphygmocor | INT: 1x4 min cycling at 90% peak oxygen consumption [V̇O2peak] | 45min/day,  3days/week,  12 weeks | | YES | Sham placebo | | ②, ⑤, ⑥ |
| Adams 2017^[11]^ | Canada | Cancer Survivors | | 63(0) | 43.5(10.3) | Complior | INT: uphill treadmill walking/running, with 75% to 95% of VO2peak | 40min/day, 3days/week,  12 weeks | | YES | Usual care | | ②, ⑤, ⑥ |
| Taha  2023^[12]^ | Saudi Arabia | Obese Hypertension | | 60(60) | 48.6(4.3) | Mobil-O-Graph | INT: cycling at 85-90% of PHR in ters with 3mins active recovery at 60 - 70% of PHR | 45min/day, 3days/week,  12 weeks | | YES | No exercise | | ④, ⑤, ⑥ |
| Bouaziz 2013^[13]^ | Brazil | Older persons | | 60(44) | 73.6(2.9) | R wave of an ECG | INT: 6×4-min at first ventilatory threshold (VT1) intensity + 1-min at 40% of VT | 30min/day, 2days/wee,  9.5 weeks | | NO | No exercise | | ②, ⑤, ⑥ |
| Millen  2013^[14]^ | South Africa | Obese individuals | | 35(11) | 44.3(6.4) | R wave of an ECG | AE: 60 and 75 % of VO2peak on a stationary bike and/or a treadmill | 50min/day, ≥3days/week ,12 weeks | | YES | No exercise | | ③, ⑤, ⑥ |
| Kearney 2013^[15]^ | Northern Ireland | Overweight adults | | 77(58) | 45(7.1) | SphygmoCor | AE: walking on a treadmill at a constant self-selected speed. Increasing the incline by 2.5% at 3-min intervals until 80% of the age-predicted max HR. | 30min/day, 5days/week,  16 weeks | | YES | Stretching exercises | | ④,⑤, ⑥ |
| Nualnim 2012^[16]^ | USA | Older persons | | 43(32) | 60.3(7.9) | VP-2000 | AE: swimming ,40 to 45 min, at 70% to 75% of max HR | 45min/day ,3-4days/week,  12weeks | | YES | Stretching exercises | | ②, ⑤, ⑥ |
| Zhou 2010^[17]^ | China | Older Persons | | 35(0) | 74.7(3.5) | SphymgmoCor | AE: treadmill training at 50–60 % of maxHR | 45min/day, 3days/week,  12weeks | | NO | No exercise | | ④,⑤, ⑥ |
| Madden  2013^[18]^ | Canada | Older persons  hypertension, hyperlipidemia | | 52(22) | 69(4.3) | Complior | AE: exercise treadmill (60min, at 60–75% of HRR) | 60min/day, 3days/week, 8weeks | | YES | Usual care | | ②, ⑤, ⑥ |
| Horner 2015^[19]^ | USA | Obese adolescents | | 81(40) | 14.8(1.8) | Complior | AE: exercise on a treadmill, elliptical or stationary bike at 60–75% of VO2peak. RT: 10 whole body exercises (2 sets of 8–12 repetitions) | 60min/day, 3days/week,  12 weeks | | YES | No exercise | | ②, ⑤, ⑥ |
| Huang 2018^[20]^ | China | Obesity young persons | | 32(0) | 20.7(1.1) | VP-1000 | AE: cycling, walking/jogging, elliptical), 50min, at 60 – 85%HR | 50min/day, 4days/week,  12weeks | | YES | No exercise | | ④,⑤, ⑥ |
| Shin 2015^[21]^ | South Korea | Older persons | | 56(56) | 65.2(5.6) | SphygmoCor | AE: Tai Chi exercise (included Twelve Movement, such as knee flexion; straight and extended head and trunk; combined rotation of head, and extremities) | 60min/day, 1days/week,  12weeks | | no  supervised | usual care | | ②, ⑤, ⑥ |
| Davis 2019^[22]^ | USA | Overweight  children | 175(106) | | 9.7(0.7) | SphygmoCor | AE: aerobic activities (e.g., tag, jump rope) for 40 min each day, average daily HR > 140 beats/min. | 55min/day, 3days/week,  16weeks | | YES | Usual care | | ②, ⑤, ⑥ |
| Chen  2018^[23]^ | China | Older persons | | 42(34) | 66.5(3.2) | VP-1000 | AE: composed of 5 minutes of warm-up, 45 minutes ofconditioning, and 5 minutes of cooldown at 50%-60% of the peak oxygen uptake (VO2peak) | 60min/day, 6days/week,  24weeks | | YES | No exercise | | ④,⑤, ⑥ |
| Madden 2009^[24]^ | Canada | Older persons | | 40(19) | 71.4(0.7) | transducer  measures | AE: exercise on a treadmill and a cycle ergometer, with 60–75% HRR | 60min/day, 3days/week,  12weeks | | YES | No exercise | | ②, ⑤, ⑥ |
| 0’Connor 2017^[25]^ | UK | KTR | | 46(19) | 51.8(12.5) | Skidmore | AE: individually tailored exercise training | 30min/day, 3days/week,  12weeks | | YES | usual care | | ②, ⑤, ⑥ |
| Jaime 2017^[26]^ | USA | Older persons | | 19(19) | 66(2.5) | SphygmoCor | RT: consisted of leg press, leg extension, leg flexion, and calf raises exercises at 40% of the 1RM | 30min/day,  3 days/week,  12 weeks | | YES | No exercise | | ②, ⑤, ⑥ |
| Croymans  2013^[27]^ | USA | Obese young men | | 36(0) | 21.5(0.8) | SphygmoCor | RT: 3 phase 2-3 sets, with 8–15 repetitions of each exercise, at 100% approximated 12–15 RM or 6-8RM | 60min/day,  3 days/week,  12 weeks | | YES | No exercise | | ②, ⑤, ⑥ |
| DeVallance  2016^[28]^ | USA | Metabolic syndrome | | 29(20) | 51(14.5) | SphygmoCor | RT: six exercises (leg press, chest press, lat pull down, leg curl, shoulder press, and leg extension) on body master weight. | 45min/day,  3 days/week,  8 weeks | | YES | No exercise | | ②, ⑤, ⑥ |
| Greenwood  2015^[29]^ | UK | KTR | | 60(26) | 54.3(10.5) | Complior | RT: high-intensity resistance training at 80% one-rep max. | 40min/day, 2days/week,  12weeks | | YES | usual care | | ②, ⑤, ⑥ |
| Beck2013^[30]^ | USA | Hypertension | | 43(13) | 21.1(0.7) | SphygmoCor | RT: 3 min of walking at 65% of maxHR and 2 min running at incline at 85% of maxHR for a total of nine 5-minute intervals. | 60min/day, 3days/week,  8 weeks | | NO | No exercise | | ②, ⑤, ⑥ |
| Fernandez 2018^[31]^ | USA | Obese women | | 11(11) | 23.5(4.2) | SphygmoCor | RT: 50min ,3 sets of 10 reps at 70-85% 1 RM. | 50min/day, 3days/week,  3 weeks | | YES | Usual care | | ①, ⑤, ⑥ |
| Jaime 2019^[32]^ | USA | postmenopausal women | | 20(20) | 65.6(3.2) | VP-2000 | RT: leg press, leg extension, leg flexion, and calf raise exercises initially prescribed at 40% of the 1RM, with the max goal of 15 reps. | 35min/day, 3days/week,  12 weeks | | YES | Usual care | | ②, ⑤, ⑥ |
| Ramírez 2020^[33]^ | Spain | Overweight adults | | 57(34) | 40.78(7.1) | SphygmoCor | RT: 12–15 reps per set of 6 exercises, A 60-second recovery at 50–70%1RM | 40min/day, 3days/week,  12 weeks | | NO | Usual care | | ① |
| Hetheringtn 2019^[34]^ | Portugal | T2D | | 63(28) | 52(6.6) | Complior | CT: INT (1 min of cycling at 90% of HRR,  followed by a 1 min rest period at 40–60% of HRR) +RT (10–12 reps of upper- and lower exercises) | 30min/day, 3days/week,  48 weeks | | YES | Usual care | | ①, ⑤, ⑥ |
| Wong 2017^[35]^ | USA | Obese  Adolescent Girls | | 30(30) | 15.3(1.2) | SphygmoCor | CT: Combined exercise consisted of 20 minutes of various resistant band exercises | 60min/day, 3days/week,  12 weeks | | YES | No exercise | | ④ |
| Magalhães 2019^[36]^ | Portugal | T2D | | 80(38) | 58.6(7.5) | Complior | CT: INT (continuous cycling at 40 to 60% of the  HRR) +RT (1 set of 10–12 repetitions of upper and  lower limbs exercises.) | 34min/day, 3days/week,  48 weeks | | YES | Usual care | | ②, ⑤, ⑥ |
| Lee 2015^[37]^ | South Korea | Chronic  Poststroke Hemiparesis | | 26(0) | 64(6.5) | SphygmoCor | CT: a 5-min warm-up, a 10-min stretching exercise, a 20-minresistance exercise, a 20-min aerobic exercise, and a 5-min cool down. | 50min/day, 3days/week,  16 weeks | | YES | No exercise | | ④, ⑤, ⑥ |
| Shiotsu 2018^[38]^ | Japan | older men | | 40(0) | 70.5 (3.5) | VaSera | CT: AE (cycling at 60% of HR) + RT (3 sets of 8–12 reps for 5 different exercises, 70–80% of 1RM) | 45min/day, 2days/week,  10 weeks | | YES | No exercise | | ② |
| Stewart 2005^[39]^ | USA | Older Persons | | 104(53) | 63.6(5.7) | SphygmoCor | CT: RT (2 sets of 10-15 rep at 50% 1RM) +AE (running, 45min at 60% to 90% of maxHR) | 90min/day, 3days/week,  24 weeks | | NO | Usual care | | ①, ⑤, ⑥ |
| Park 2020^[40]^ | Korea | Obese Older Men | | 20(0) | 68.8(0.9) | Complior | CT: RT (elastic-band ,3 sets of 10-15 reps at 60-70% 1RM ,30-40min) +AE (running and bicycle ,60min at 60%–70% of HRmax | 90min/day, 3 days/week,  12 weeks | | YES | No exercise | | ④, ⑤, ⑥ |
| Son 2016^[41]^ | South Korea | Postmenopausal women with Hypertension | | 20(20) | 75.3(3.5) | SphygmoCor | CT: RT (20 min of various resistant band exercises) +AE (30 minutes of walking, 40% to 50% HR in 1-4 week, 60% to 70% HR) | 70min/day,  3 days/week,  12 weeks | | NO | Usual care | | ④, ⑤, ⑥ |
| Dobrosielski 2012^[42]^ | USA | T2D | | 140(81) | 56.5(6) | SphygmoCor | CT: RT (2 sets of 7 exercises at 10 to 15 reps per exercise at 50 % of 1-rep max) +AE (45min, at 60 % to 90 % of maxHR) | 70min/day, 3 days/week,  26 weeks | | YES | No exercise | | ②,⑤, ⑥ |
| Loimaala 2008^[43]^ | Finland | Type 2 Diabetes | | 48(0) | 53.8(5.6) | Complior | CT: AE (jogging or walking, at 65% to 75% VO2max) +RT (12 different resistance training, 3 to 4 sets with 10 to 12 reps at 60%-80%1RM) | 30min/day, 4 days/week,  96 weeks | | YES | Usual care | | ②, ⑤ |

Note.①: pulse wave velocity; ②: carotid-femoral pulse wave velocity; ④: brachial arteries pulse wave velocity; ⑤: systolic blood pressure; ⑥: diastolic blood pressure.

Abbreviation: T2D: Type 2 Diabetes; KTR: Kidney Transplant Recipients.

## Appendix 5. Risk of bias assessment.

**Appendix 5.1** Risk of bias assessment table

| Study | Random sequence generation | | Allocation concealment | blinding of outcome assessors | incomplete outcome | | selective outcome reporting | | other risks  of bias | Risk category |
| --- | --- | --- | --- | --- | --- | --- | --- | --- | --- | --- |
| Guimarães 2010 | | low | unclear | low | | high | unclear | low | | Moderate |
| Bouaziz 2019 | | low | unclear | low | | low | unclear | low | | Low |
| Aghaei 2019 | | unclear | unclear | low | | unclear | low | low | | Low |
| Zhuang 2023 | | unclear | unclear | unclear | | high | unclear | low | | Moderate |
| Rodriguez 2017 | | unclear | unclear | low | | low | unclear | low | | Low |
| Deiseroth 2019 | | low | unclear | unclear | | low | unclear | low | | Low |
| Zhang 2022 | | high | unclear | unclear | | high | unclear | low | | High |
| Kim 2017 | | low | low | low | | high | unclear | low | | Moderate |
| Bellia 2017 | | unclear | unclear | unclear | | low | low | high | | Moderate |
| Way 2020 | | low | low | low | | low | unclear | low | | Low |
| Adams 2017 | | low | unclear | unclear | | low | unclear | low | | Low |
| Taha 2023 | | low | low | low | | low | unclear | low | | Low |
| Hetheringtn 2019 | | low | low | low | | unclear | unclear | low | | Low |
| Wong 2017 | | unclear | unclear | unclear | | low | unclear | high | | High |
| Magalhães 2019 | | low | low | unclear | | low | unclear | low | | Low |
| Lee 2015 | | low | low | low | | low | low | high | | Moderate |
| Shiotsu 2018 | | unclear | unclear | low | | low | unclear | low | | Low |
| Stewart 2005 | | unclear | unclear | low | | low | unclear | low | | Low |
| Park 2020 | | low | low | low | | low | unclear | high | | Moderate |
| Bouaziz 2013 | | unclear | unclear | low | | low | low | high | | Moderate |
| Son 2016 | | unclear | unclear | low | | low | unclear | high | | High |
| Dobrosielski 2012 | | unclear | unclear | low | | low | unclear | low | | Low |
| Greenwood 2015 | | low | low | unclear | | low | unclear | high | | Moderate |
| Loimaala 2008 | | unclear | unclear | low | | low | unclear | low | | Low |
| Jaime 2017 | | unclear | unclear | low | | low | low | low | | Low |
| Croymans 2013 | | low | low | low | | low | unclear | low | | Low |
| DeVallance 2016 | | low | low | low | | low | unclear | low | | Low |
| Zhou 2010 | | low | low | low | | low | unclear | low | | Low |
| Horner 2015 | | unclear | unclear | low | | low | unclear | low | | Low |
| Beck 2013 | | unclear | unclear | unclear | | low | unclear | low | | Moderate |
| Fernandez 2018 | | unclear | unclear | unclear | | low | unclear | high | | High |
| Jaime 2019 | | unclear | unclear | low | | low | unclear | low | | Low |
| Ramírez 2020 | | low | low | unclear | | high | low | low | | Moderate |
| Millen 2013 | | unclear | unclear | low | | low | low | low | | Low |
| Kearney 2013 | | unclear | low | low | | high | unclear | low | | Moderate |
| Huang 2018 | | unclear | unclear | unclear | | low | unclear | low | | Moderate |
| Nualnim 2012 | | unclear | unclear | low | | unclear | low | low | | Low |
| Chen 2018 | | unclear | unclear | unclear | | low | unclear | high | | High |
| Madden 2013 | | low | low | low | | low | unclear | unclear | | Low |
| Madden 2009 | | unclear | unclear | low | | unclear | low | low | | Low |
| Shin 2015 | | unclear | unclear | low | | high | unclear | low | | Moderate |
| Davis 2019 | | low | low | low | | low | unclear | low | | Low |
| 0’Connor 2017 | | unclear | unclear | low | | unclear | unclear | low | | Moderate |


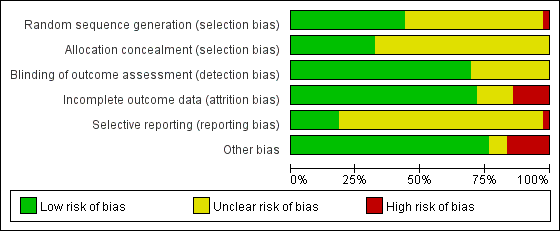


**Appendix 5.2** The overall risk of bias for all included studies

**
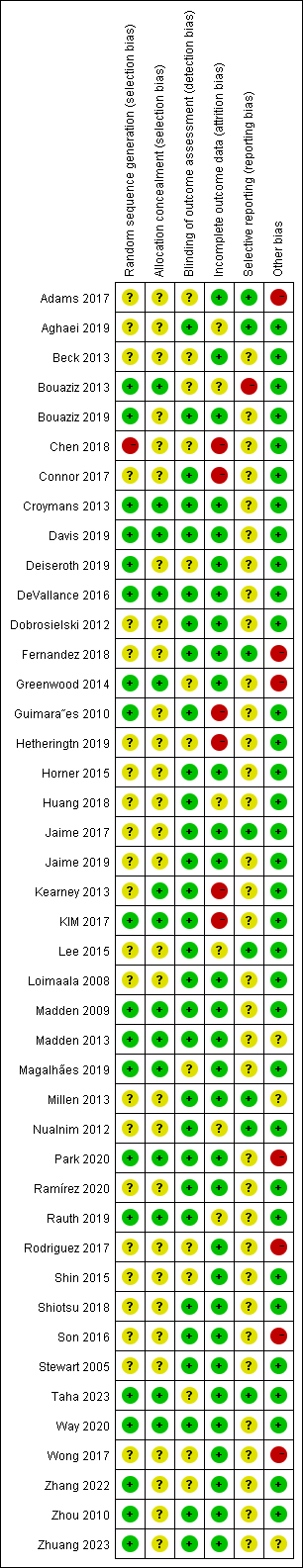
**

**Appendix 5.3** The risk of bias for each study

##

## Appendix 6. Funnel plot of Pulse Wave Velocity, Systolic Blood Pressure, and Diastolic Blood Pressure in pairwise meta-analysis.


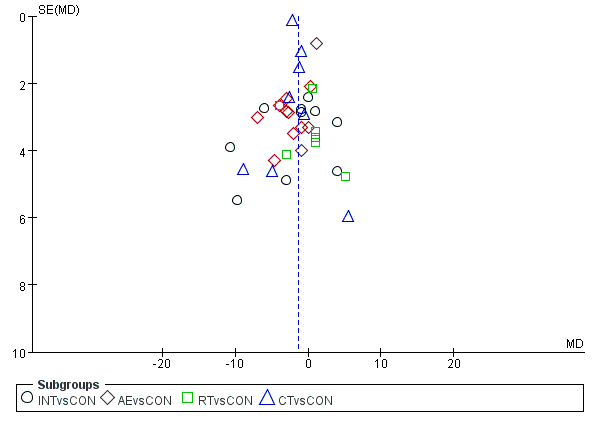


**Appendix 6.1** Funnel plot of Pulse Wave Velocity in pairwise meta-analysis.


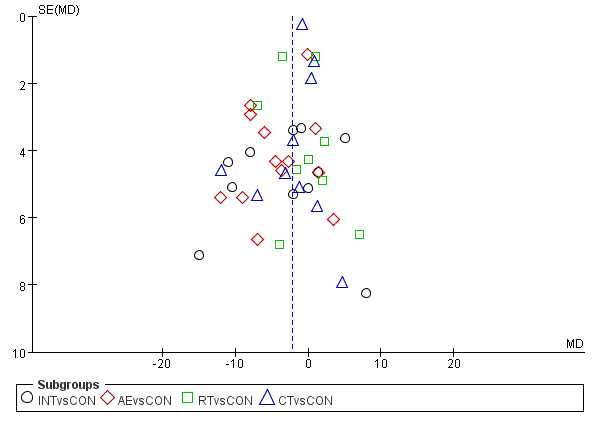


**Appendix 6.2** Funnel plot of Systolic Blood Pressure in pairwise meta-analysis.


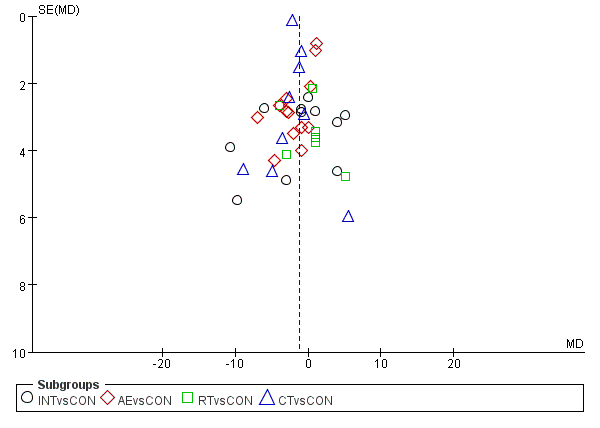


**Appendix 6.3** Funnel plot of Diastolic Blood Pressure in pairwise meta-analysis.

## Appendix 7. Forest plots for Pulse Wave Velocity, Systolic Blood Pressure, and Diastolic Blood Pressure.


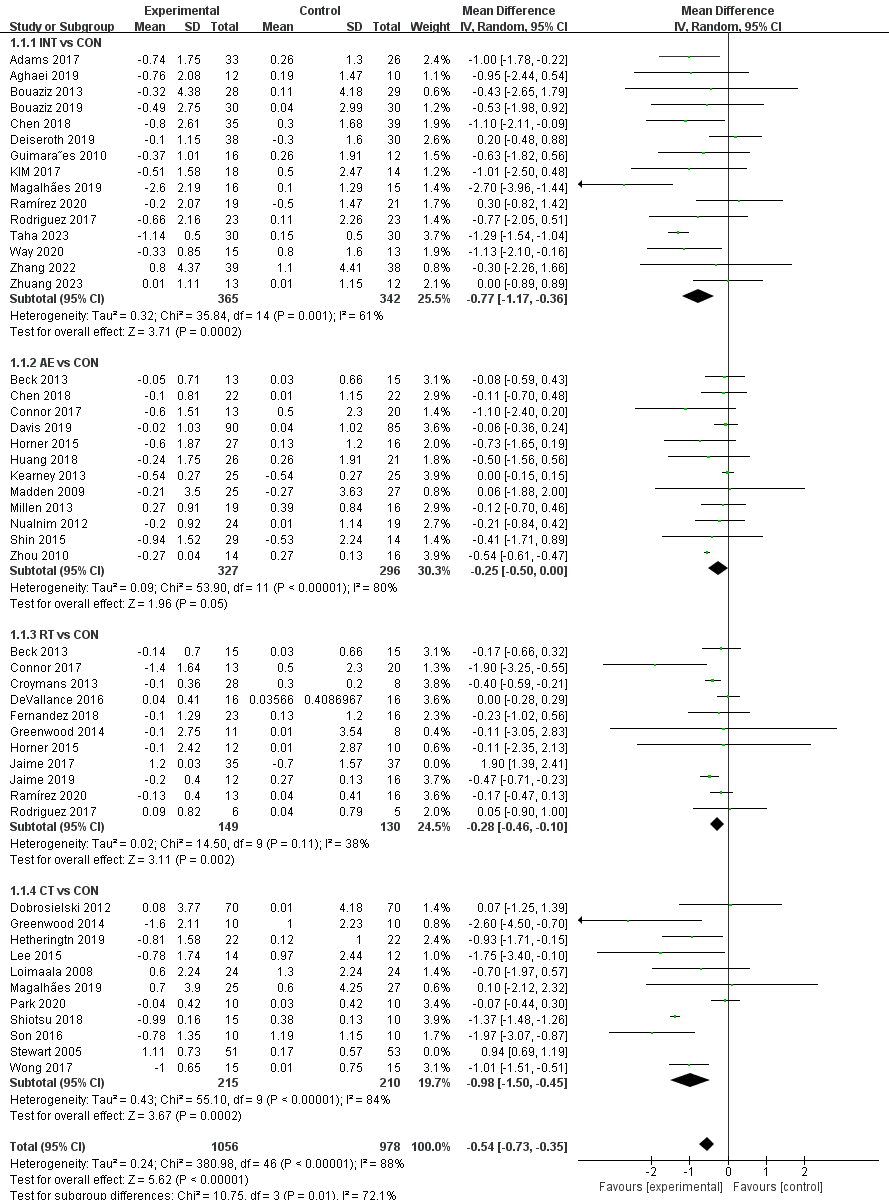


**Appendix 7.1** forest plot of Pulse Wave Velocity.

**Appendix 7.2** sensitivity analysis for Pulse Wave Velocity.

| Intervention | Excluded literature | WMD (95% CI) | I^2^ | P overall change |
| --- | --- | --- | --- | --- |
| INT vs CON | Adams 2017 | -0.75 [-1.19, -0.30] | 64 | 0.0006 |
|  | Aghaei 2019 | -0.76 [-1.18, -0.33] | 64 | 0.0006 |
|  | Bouaziz 2013 | -0.78 [-1.20, -0.36] | 63 | 0.0007 |
|  | Bouaziz 2019 | -0.78 [-1.21, -0.36] | 63 | 0.0007 |
|  | Chen 2018 | -0.74 [-1.18, -0.30] | 64 | 0.0006 |
|  | **Deiseroth 2019** | **-0.89 [-1.26, -0.53]** | **43** | **0.04** |
|  | Guimara˜es 2010 | -0.78 [-1.21, -0.35] | 63 | 0.0007 |
|  | KIM 2017 | -0.76 [-1.18, -0.33] | 64 | 0.0006 |
|  | Magalhães 2019 | -0.66 [-1.04, -0.27] | 55 | 0.0007 |
|  | Ramírez 2020 | -0.85 [-1.25, -0.44] | 57 | 0.004 |
|  | Rodriguez 2017 | -0.77 [-1.20, -0.34] | 64 | 0.0007 |
|  | **Taha 2023** | **-0.69 [-1.11, -0.27]** | **46** | **0.03** |
|  | Way 2020 | -0.74 [-1.18, -0.30] | 64 | 0.0006 |
|  | Zhang 2022 | -0.78 [-1.20, -0.37] | 63 | 0.0007 |
|  | Zhuang 2023 | -0.84 [-1.25, -0.43] | 58 | 0.004 |
| AE vs CON | Beck 2013 | -0.27 [-0.54, -0.00] | 81 | <0.0001 |
|  | Chen 2018 | -0.26 [-0.53, 0.00] | 81 | <0.0001 |
|  | Connor 2017 | -0.22 [-0.48, 0.03] | 81 | <0.0001 |
|  | Davis 2019 | -0.28 [-0.55, -0.01] | 79 | <0.0001 |
|  | Horner 2015 | -0.22 [-0.48, 0.04] | 81 | <0.0001 |
|  | Huang 2018 | -0.24 [-0.50, 0.02] | 81 | <0.0001 |
|  | **Kearney 2013** | **-0.30 [-0.52, -0.09]** | **43** | **0.06** |
|  | Madden 2009 | -0.26 [-0.51, -0.00] | 81 | <0.0001 |
|  | Millen 2013 | -0.26 [-0.53, 0.00] | 81 | <0.0001 |
|  | Nualnim 2012 | -0.25 [-0.52, 0.01] | 81 | <0.0001 |
|  | Shin 2015 | -0.25 [-0.50, 0.01] | 81 | <0.0001 |
|  | Zhou 2010 | -0.06 [-0.18, 0.06] | 0 | 0.78 |
| CT vs CON | Dobrosielski 2012 | -0.77 [-1.60, 0.07] | 97 | <0.0001 |
|  | Greenwood 2014 | -0.66 [-1.48, 0.16] | 97 | <0.0001 |
|  | Hetheringtn 2019 | -0.67 [-1.52, 0.18] | 97 | <0.0001 |
|  | Lee 2015 | -0.61 [-1.44, 0.22] | 97 | <0.0001 |
|  | Loimaala 2008 | -0.69 [-1.53, 0.14] | 97 | <0.0001 |
|  | Magalhães 2019 | -0.75 [-1.56, 0.07] | 97 | <0.0001 |
|  | **Park 2020** | -0.77 [-1.67, 0.13] | 97 | <0.0001 |
|  | Shiotsu 2018 | -0.59 [-1.31, 0.12] | 90 | <0.0001 |
|  | Son 2016 | -0.57 [-1.40, 0.27] | 97 | <0.0001 |
|  | **Stewart 2005** | -0.90 [-1.42, -0.39] | 83 | <0.0001 |
|  | Wong 2017 | -0.66 [-1.54, 0.22] | 97 | <0.0001 |

PWV, pulse wave velocity; WMD, weighted mean difference; Bold font indicates sensitive sources.

**Appendix 7.3** Subgroup analyses assessing potential moderating factors for PWV in studies included in the meta-analysis.

| Group | Studies | | | | PWV(m/s) | | | | |
| --- | --- | --- | --- | --- | --- | --- | --- | --- | --- |
|  | Number | | | Reference | WMD (95%) | I^2^ | P overall change | P for sub dif | P for m |
| Intensity | | | | | | | | | |
| high | 4 | | | 42,43,38,39 | 0.32[0.08, 0.56] | 96 | 0.000 | 0.000 | 0.695 |
| moderate | 7 | | 29,34,37,36,40,41,35 | | -0.67[-0.95, -0.38] | 61 | 0.018 |  |  |
| Duration(mins) | | | | | | | | | |
| >2000 | 9 | 42,34,37,43,36,40,41,39,35 | | | 0.00[-0.19, 0.18] | 89 | 0.000 | 0.000 | 0.037 |
| <2000 | 2 | | | 29,38 | -2.05[-2.96, -1.15] | 97 | 0.000 |  |  |
| Content of intervention | | | | | | | | | |
| RT+AE | 6 | | | 42,29,37,40,41,39 | 0.18[-0.04, 0.41] | 92 | 0.000 | 0.000 | 0.691 |
| AE+RT | 3 | | | 43,38,35 | -0.92[-1.38, -0.46] | 95 | 0.000 |  |  |
| INT+RT | 2 | | | 34,36 | -0.30[-0.70, 0.11] | 67 | 0.081 |  |  |
| Sex | | | | | | | | | |
| Man(only) | 4 | | | 37,43,40,38 | -0.61[-1.01, -0.20] | 92 | 0.000 | 0.000 | 0.227 |
| Woman(only) | 2 | | | 41,35 | -1.49[-2.12, -0.86] | 0 | 0.842 |  |  |
| Both sexes | 5 | | | 42,39,34,36,39 | 0.22[0.00, 0.43] | 92 | 0.000 |  |  |
| Age(years) | | | | | | | | | |
| >60 | 5 | | | 37,40,38,41,39 | 0.38[0.05, 0.70] | 96 | 0.000 | 0.000 | 0.691 |
| <60 | 6 | | | 42,29,34,43,36,35 | -0.29[-0.51, -0.07] | 72 | 0.004 |  |  |
| PWV assessment site | | | | | | | | | |
| cfPVW | 5 | | | 36,38,42,43,29 | -0.25[-0.48, -0.01] | 90 | 0.000 | 0.000 | 0.377 |
| baPWV | 4 | | | 35,37,40,41 | -1.04[-1.05, -0.52] | 66 | 0.055 |  |  |
| PWV | 2 | | | 34,39 | 0.72[0.37, 1.08] | 97 | 0.000 |  |  |

PWV, pulse wave velocity; WMD, weighted mean difference; P for sub dif, p for subgroup difference; P form, p value for the meta-regression analyses between subgroups.


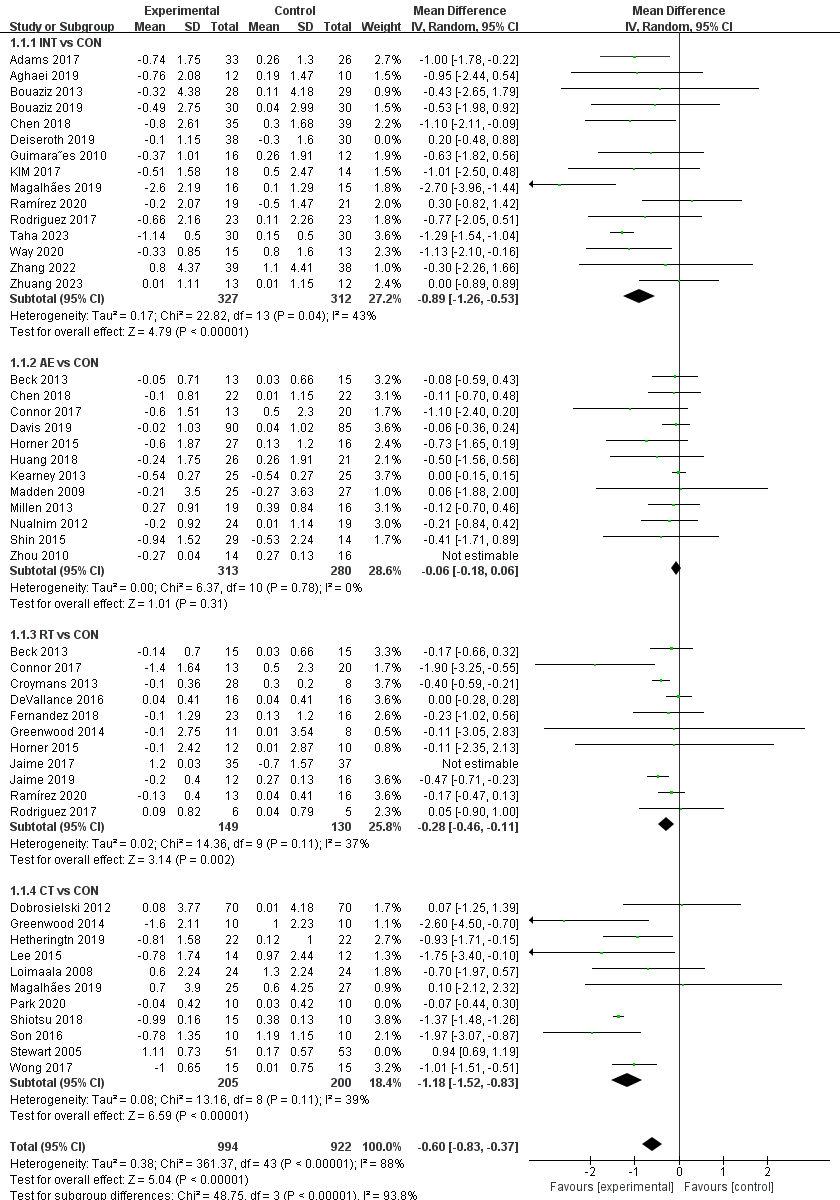


**Appendix 7.4** forest plot of Pulse Wave Velocity after Removed

Deiseroth 2019(INT), Kearney 2013(AE), Park 2020 and Stewart 2005(CT).


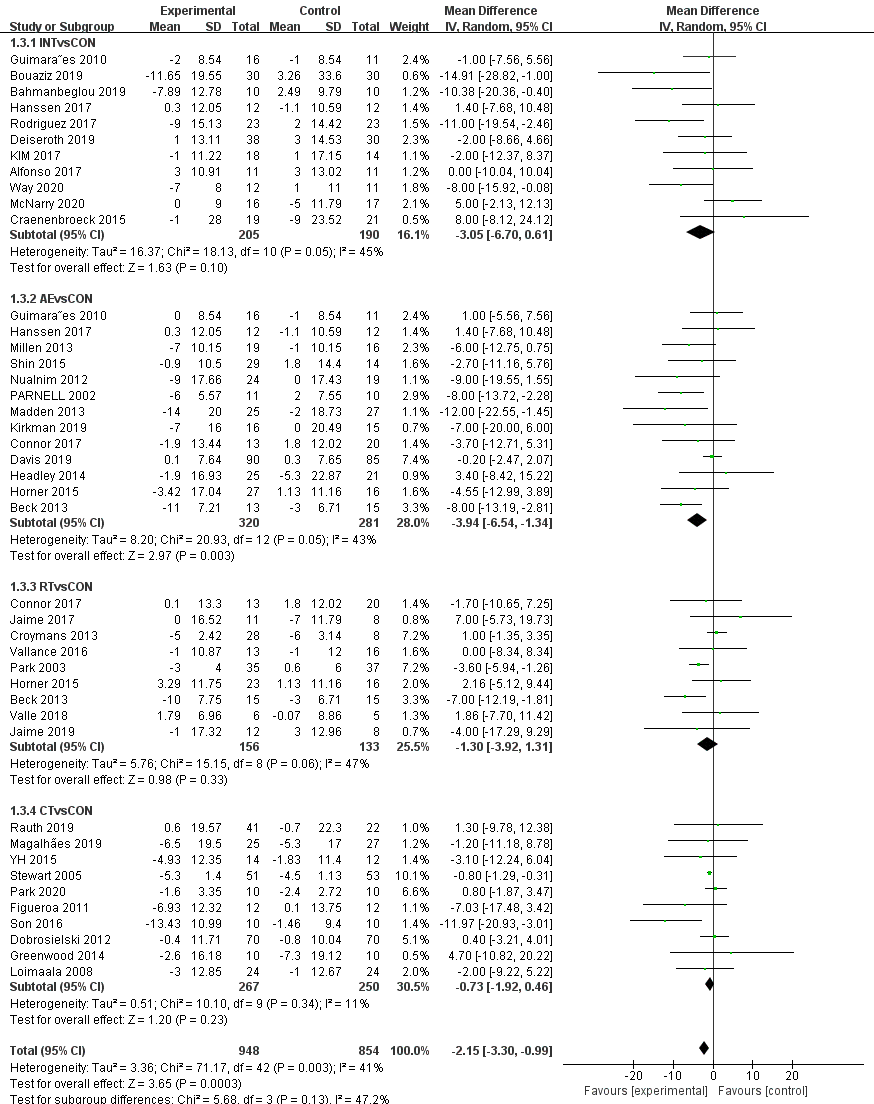


**Appendix 7.5** forest plot of Systolic Blood Pressure.


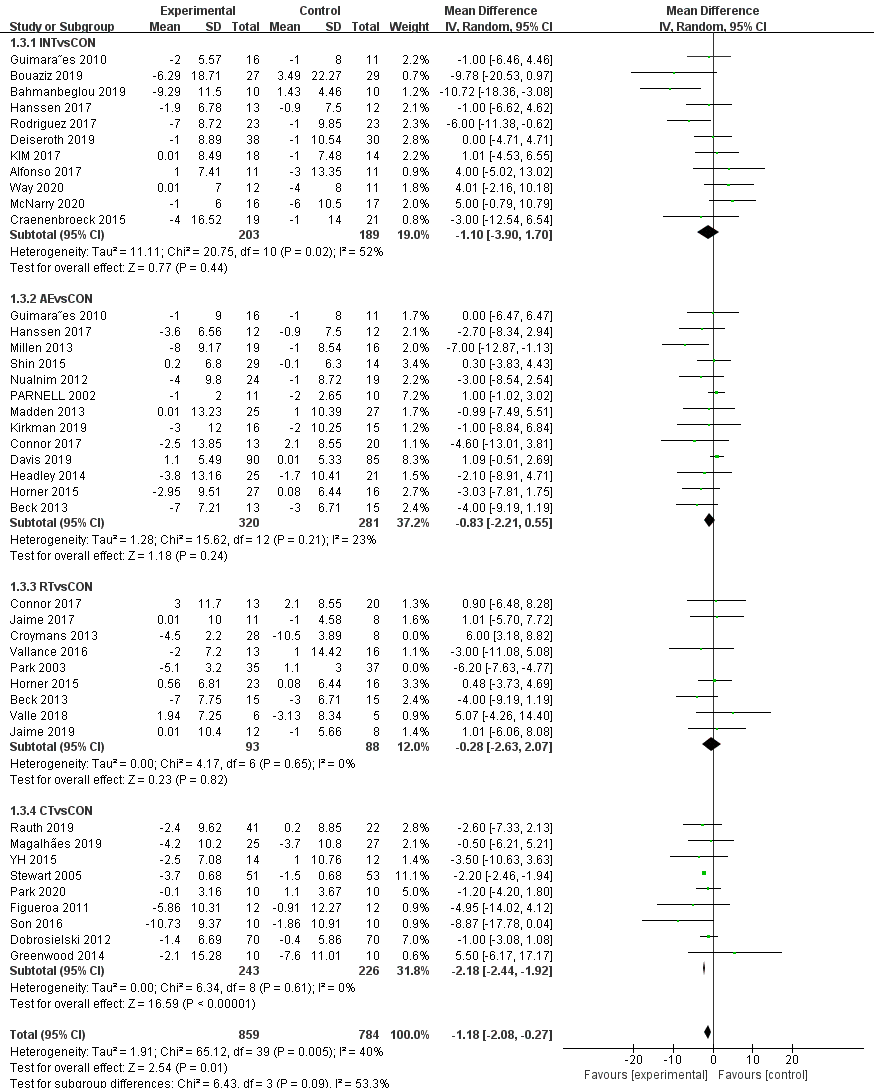


**Appendix 7.6** forest plot of Diastolic Blood Pressure.

## Appendix 8. Contributions of direct and indirect comparisons to NMA and the number of studies of each direct comparison of Pulse Wave Velocity, Systolic Blood Pressure , and Diastolic Blood Pressure.

*Note.* A means control group; B means interval training; C means aerobic exercise; D means resistance training; E means combined training.


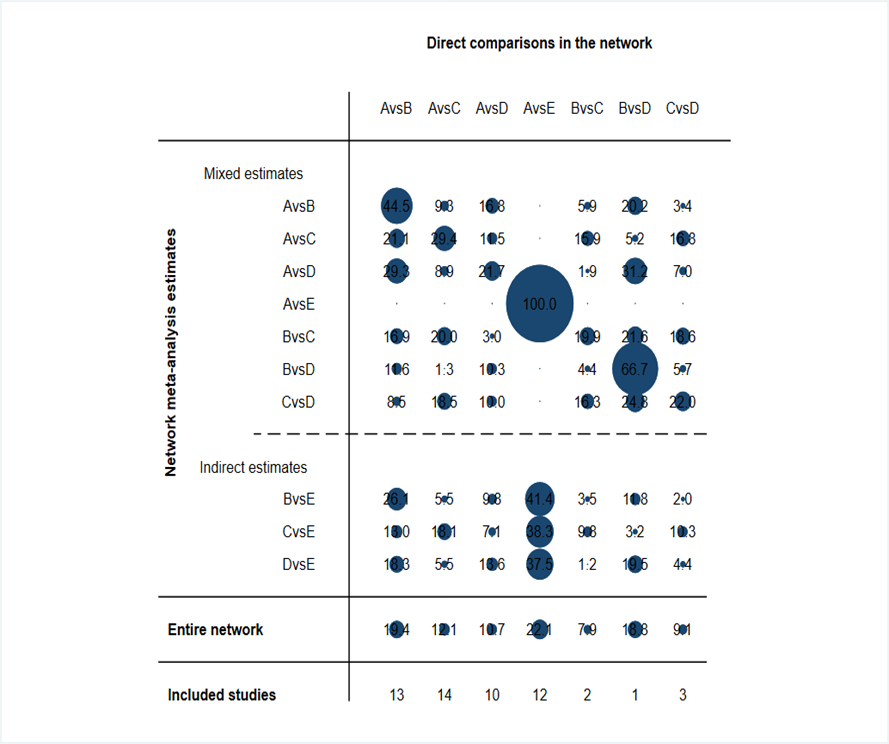


**Appendix 8.1** Contribution plot for Pulse Wave Velocity.


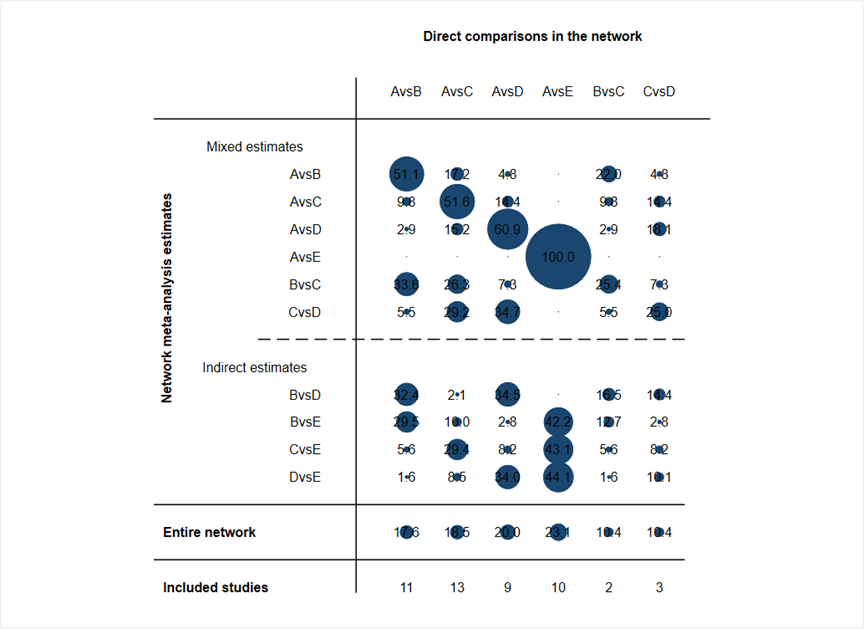


**Appendix 8.2** Contribution plot for Systolic Blood Pressure.


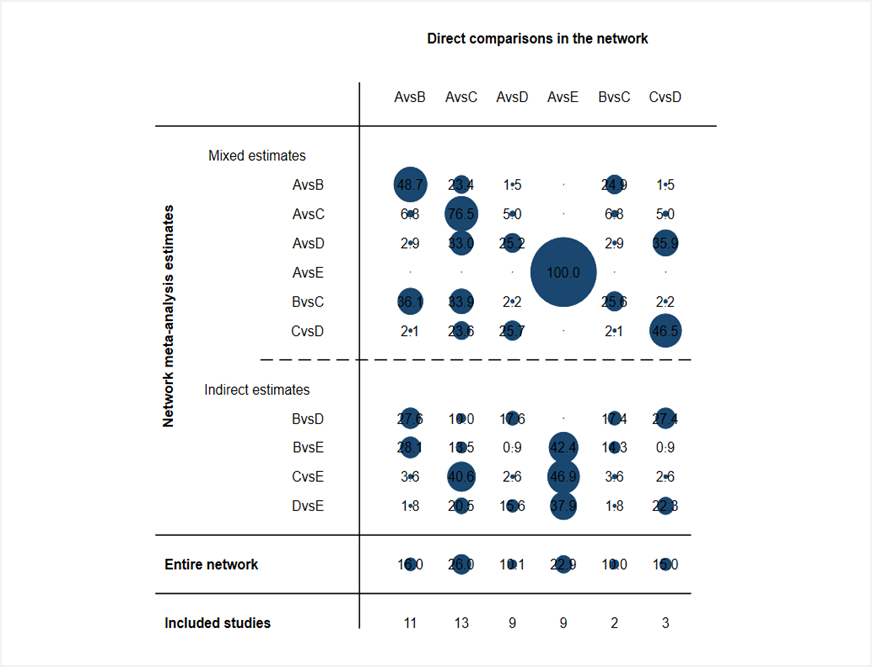


**Appendix 8.3** Contribution plot for Diastolic Blood Pressure.

## Appendix 9. Inconsistency of Pulse Wave Velocity, Systolic Blood Pressure, and Diastolic Blood Pressure tested by loop-specific heterogeneity estimates, inconsistency model and node splitting analysis.

*Note.* CON, control group; INT, interval training; AE, means aerobic exercise; RT, resistance training; CT, means combined training.

**loop-specific heterogeneity estimates.**


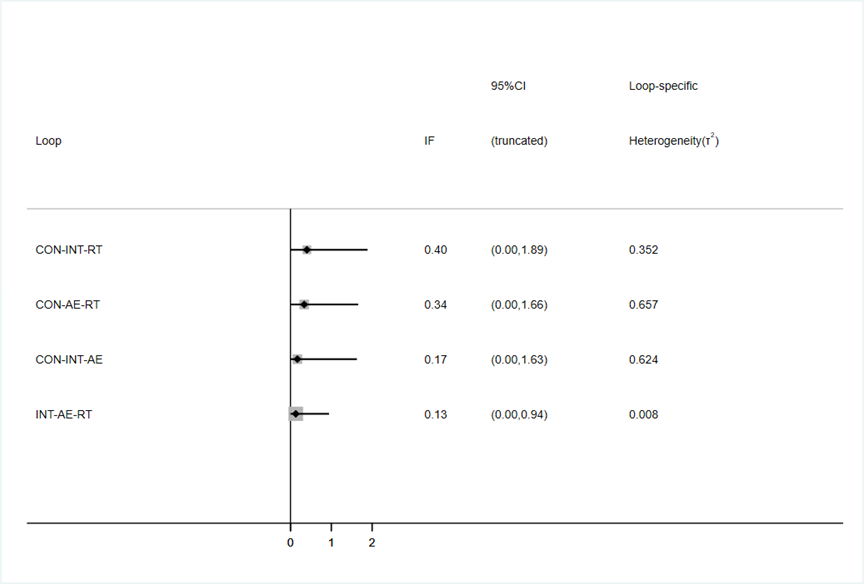


**Appendix 9.1** loop-specific heterogeneity estimates for Pulse Wave Velocity.


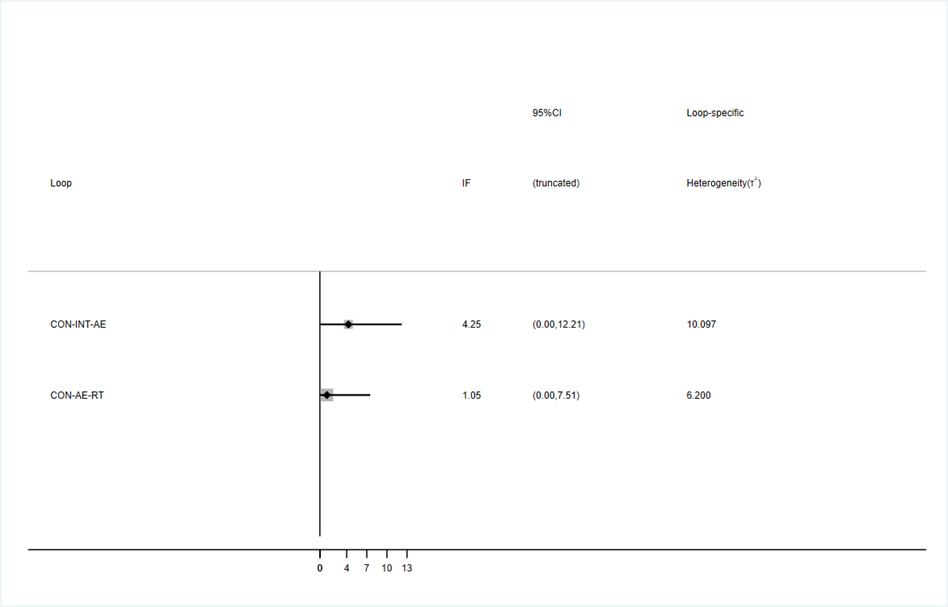


**Appendix 9.2** loop-specific heterogeneity estimates for Systolic Blood Pressure.

**
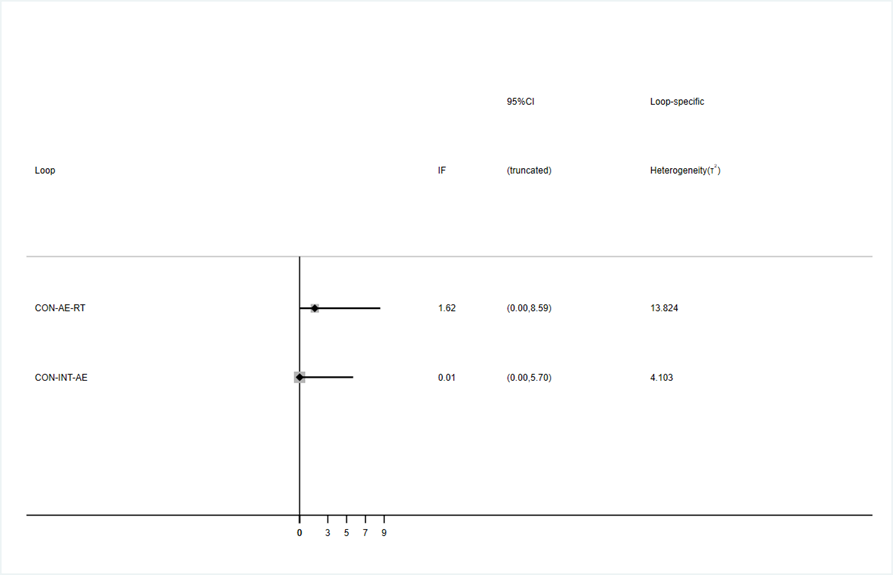
**

**Appendix 9.3** loop-specific heterogeneity estimates for Diastolic Blood Pressure.

**Inconsistency model.**

**Appendix 9.4** Inconsistency model for Pulse Wave Velocity, Systolic Blood Pressure, and Diastolic Blood Pressure.

|  | Pulse Wave Velocity | Systolic Blood Pressure | Diastolic Blood Pressure |
| --- | --- | --- | --- |
| chi2 | 1.86 | 3.32 | 1.02 |
| Prob > chi2 | 0.93 | 0.51 | 0.91 |

No evidence for the existence of significant global inconsistency

**Node splitting analysis.**

**Appendix 9.5** Node splitting analysis for Pulse Wave Velocity, Systolic Blood Pressure, and Diastolic Blood Pressure.

| Side | Direct  Coef. | Std. Err. | Indirect  Coef. | Std. Err. | Difference  Coef. | Std.Err. | P>z |
| --- | --- | --- | --- | --- | --- | --- | --- |
| Pulse Wave Velocity | | | | | | | |
| A B | -0.570 | 0.243 | -0.098 | 0.863 | -0.472 | 0.901 | 0.616 |
| A C | -0.397 | 0.219 | 0.113 | 0.759 | -0.510 | 0.793 | 0.513 |
| A D | -0.088 | 0.257 | -0.396 | 0.772 | 0.307 | 0.814 | 0.774 |
| B C | 0.014 | 0.573 | 0.236 | 0.344 | -0.222 | 0.669 | 0.765 |
| B D | 0.073 | 0.688 | 0.508 | 0.362 | -0.435 | 0.777 | 0.613 |
| C D | -0.076 | 0.464 | 0.446 | 0.378 | -0.522 | 0.599 | 0.417 |
| Systolic Blood Pressure | | | | | | | |
| A B | -3.191 | 1.530 | -12.434 | 6.033 | 9.243 | 6.268 | 0.140 |
| A C | -3.856 | 1.252 | -2.017 | 4.120 | -1.838 | 4.281 | 0.668 |
| A D | -1.297 | 1.315 | 2.969 | 5.255 | -4.266 | 5.410 | 0.430 |
| B C | 2.371 | 3.020 | -1.061 | 2.191 | 3.432 | 3.744 | 0.359 |
| C D | 3.004 | 2.620 | 2.451 | 2.155 | 0.553 | 3.387 | 0.870 |
| Diastolic Blood Pressure | | | | | | | |
| A B | -1.048 | 1.288 | -1.215 | 5.292 | 0.166 | 5.493 | 0.976 |
| A C | -1.528 | 1.051 | -1.340 | 3.695 | -0.189 | 3.832 | 0.961 |
| A D | -0.364 | 1.254 | 4.277 | 4.650 | -4.642 | 4.800 | 0.334 |
| B C | -0.382 | 2.660 | -0.489 | 1.817 | 0.107 | 3.229 | 0.974 |
| C D | 2.514 | 2.359 | 0.756 | 1.915 | 1.758 | 3.043 | 0.564 |

## Appendix 10. Forest plots of eligible comparisons of Pulse Wave Velocity , Systolic Blood Pressure , and Diastolic Blood Pressure.

*Note.* CON, control group; INT, interval training; AE, means aerobic exercise; RT, resistance training; CT, means combined training.


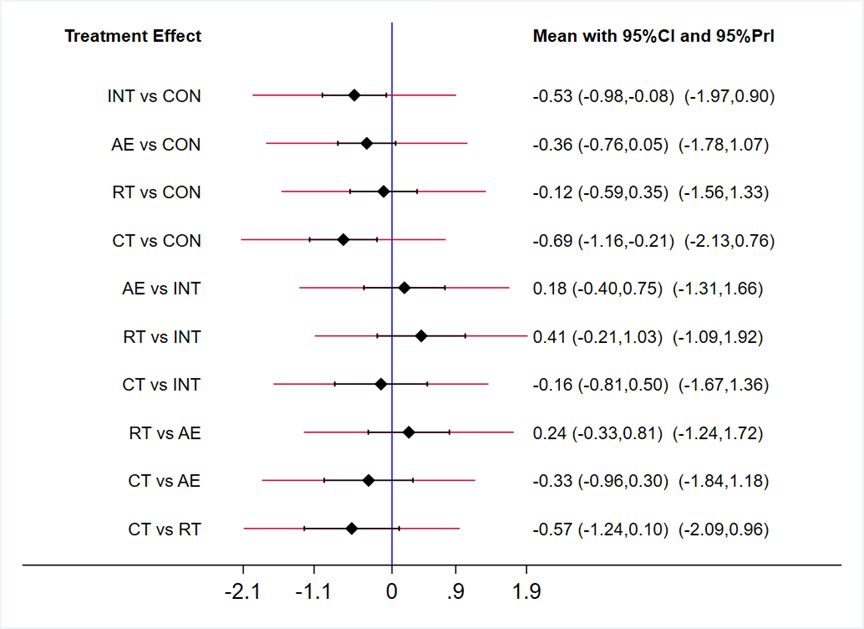


**Appendix 10.1** Forest plots of eligible comparisons of Pulse Wave Velocity.


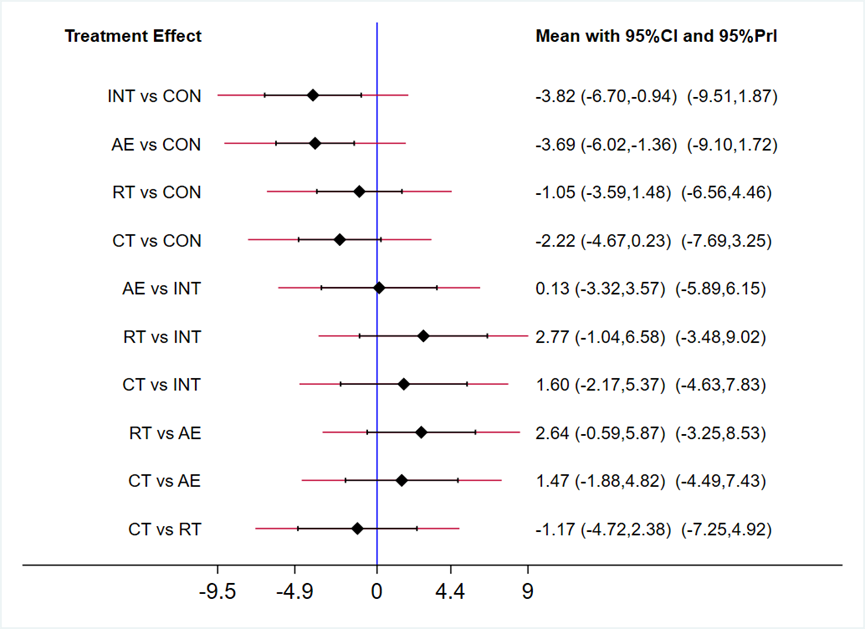


**Appendix 10.2** Forest plots of eligible comparisons of Systolic Blood Pressure.


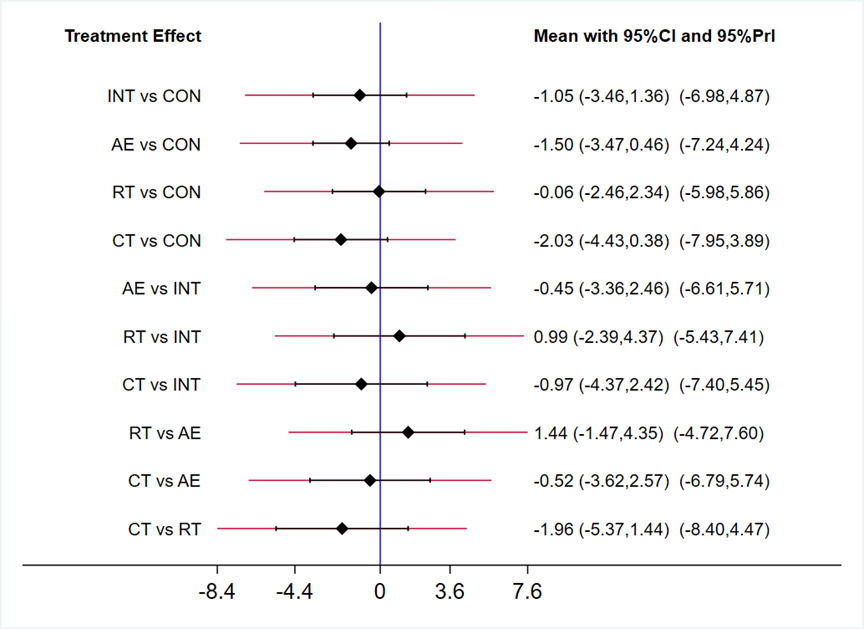


**Appendix 10.3** Forest plots of eligible comparisons of Diastolic Blood Pressure.

## Appendix 11. The funnel plot graphics of Pulse Wave Velocity, Systolic Blood Pressure, and Diastolic Blood Pressure in NMA.


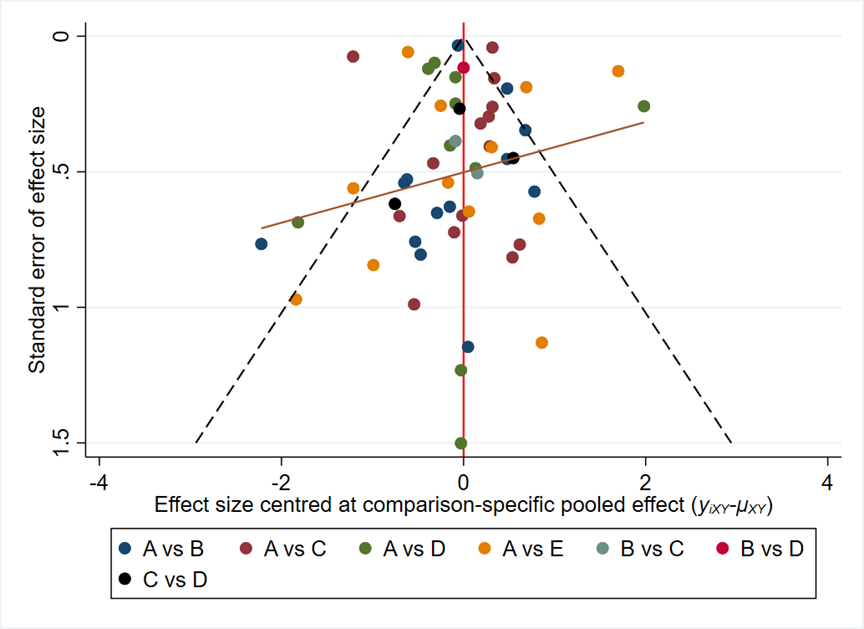


**Appendix 11.1** The funnel plot graphics of Pulse Wave Velocity.


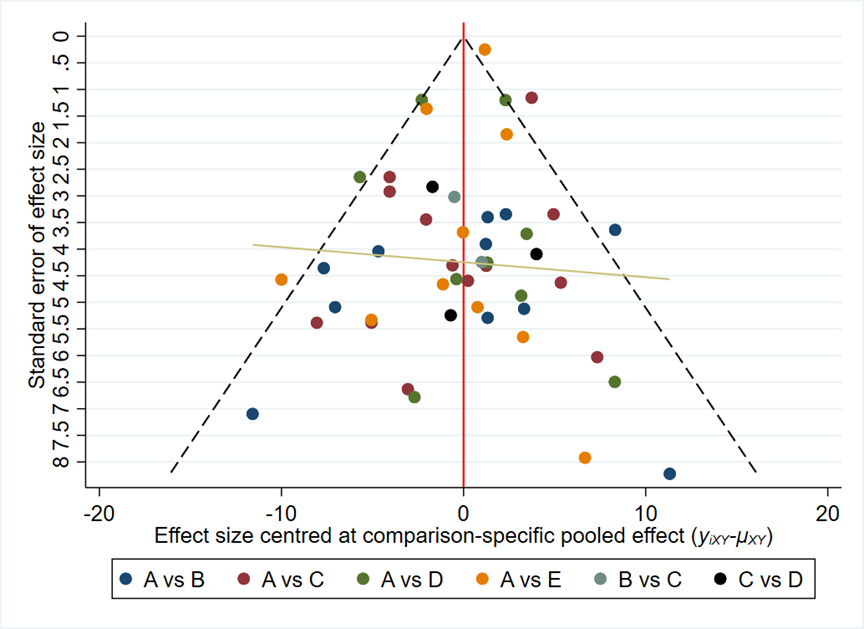


**Appendix 11.2** The funnel plot graphics of Systolic Blood Pressure.


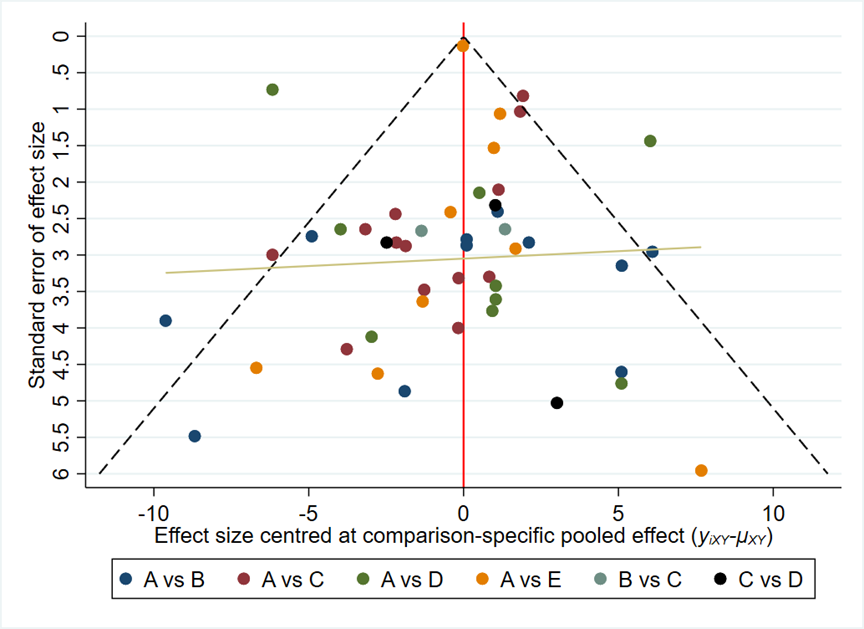


**Appendix 11.3** The funnel plot graphics of Diastolic Blood Pressure.

*Note.* A means control group; B means interval training; C means aerobic exercise; D means resistance training; E means combined training.

## Appendix 12. Area under the curve for cumulative ranking probability of each intervention on Pulse Wave Velocity, Systolic Blood Pressure, and Diastolic Blood Pressure.

*Note.* CON, control group; INT, interval training; AE, means aerobic exercise; RT, resistance training; CT, means combined training.


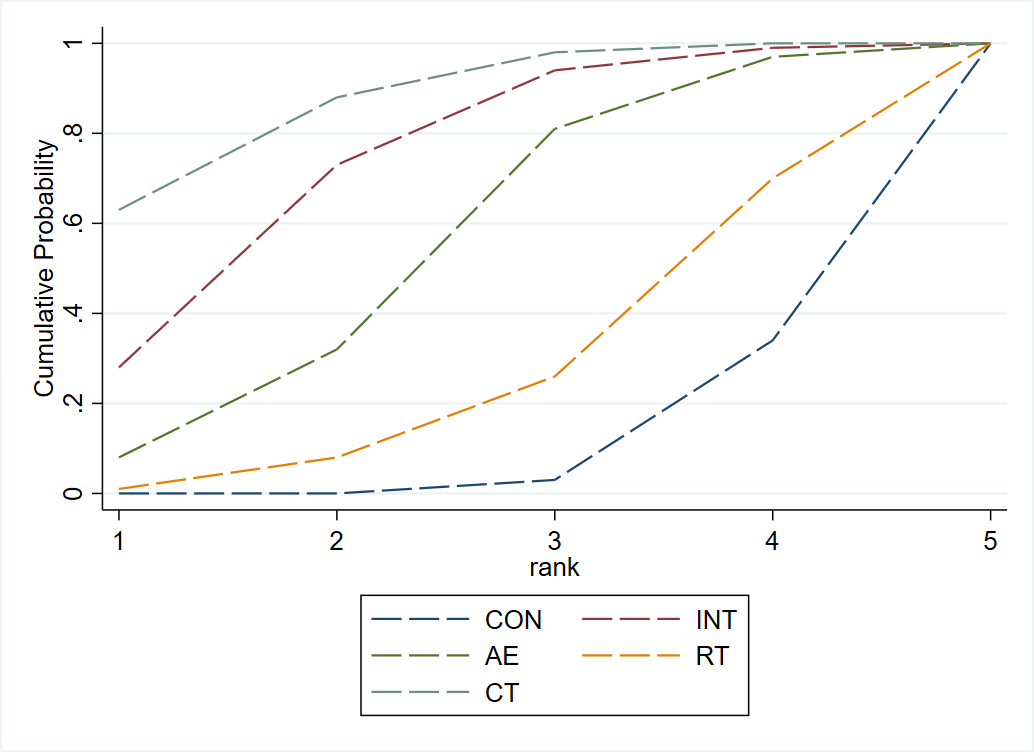


**Appendix 12.1** The cumulative ranking probability plot of each intervention on Pulse Wave Velocity.


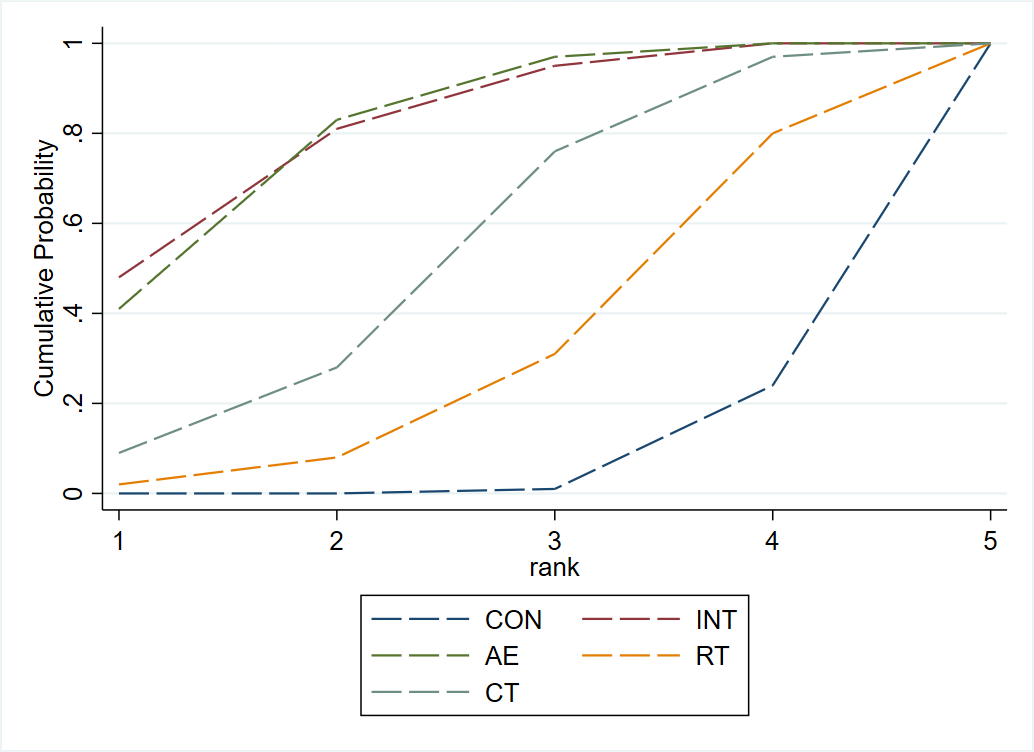


**Appendix 12.2** The cumulative ranking probability plot of each intervention on Systolic Blood Pressure.


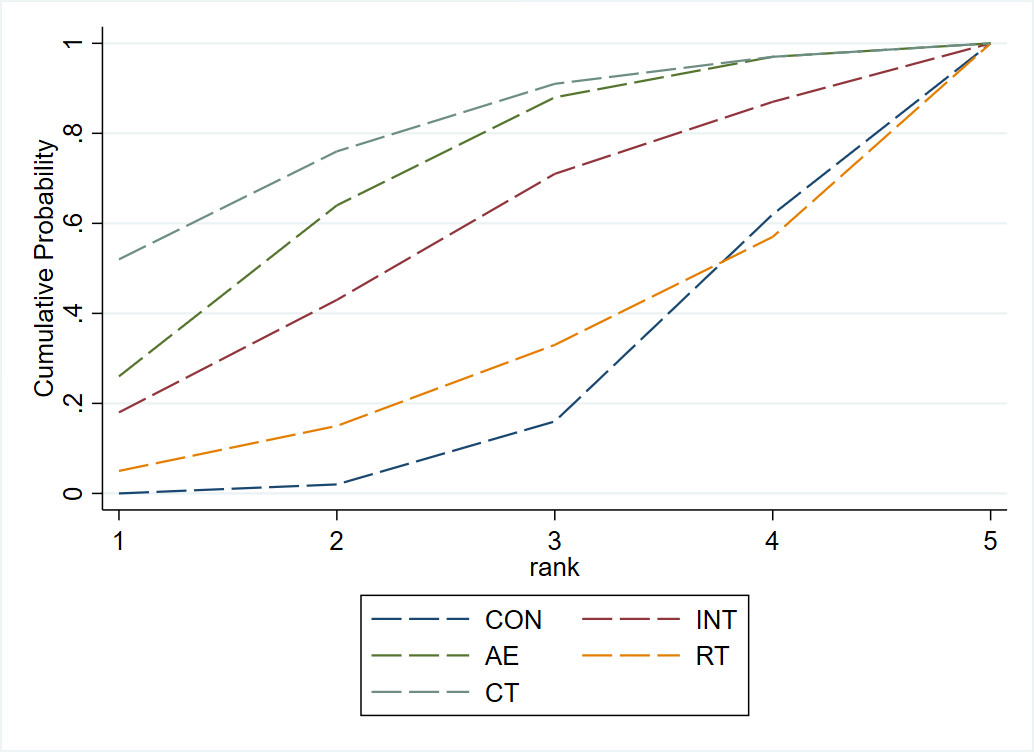


**Appendix 12.3** The cumulative ranking probability plot of each intervention on Diastolic Blood Pressure.

**Appendix** **12.4** Ranking of exercise interventions in order of effectiveness.

| Pulse Wave Velocity (49studies, N=2034) | | | | |
| --- | --- | --- | --- | --- |
| Treatment | SUCRA | PrBest (%) | | Mean Rank |
| CT | 87.2 | 63.9 | | 1.5 |
| INT | 73.1 | 27.5 | | 2.1 |
| AE | 54.2 | 7.2 | | 2.8 |
| RT | 26.3 | 1.4 | | 3.9 |
| CON | 9.3 | 0 | | 4.6 |
| Systolic Blood Pressure (46studies, N=1802) | | |  | |
| Treatment | SUCRA | PrBest (%) | | Mean Rank |
| INT | 81.3 | 48.7 | | 1.7 |
| AE | 80.4 | 41.1 | | 1.8 |
| CT | 52.5 | 8.8 | | 2.9 |
| RT | 29.7 | 1.4 | | 3.8 |
| CON | 6 | 0 | | 4.8 |
| Diastolic Blood Pressure (42studies, N=1643) | | | | |
| Treatment | SUCRA | PrBest (%) | | Mean Rank |
| CT | 78.8 | 51.3 | | 1.8 |
| AE | 69.2 | 26.6 | | 2.2 |
| INT | 54.9 | 17.8 | | 2.8 |
| RT | 27.4 | 4.2 | | 3.9 |
| CON | 19.6 | 0.1 | | 4.2 |

## Appendix 13. Network of eligible comparisons for Pulse Wave Velocity, Systolic Blood Pressure, and Diastolic Blood Pressure.

*Note.* CON, control group; INT, interval training; AE, means aerobic exercise; RT, resistance training; CT, means combined training.


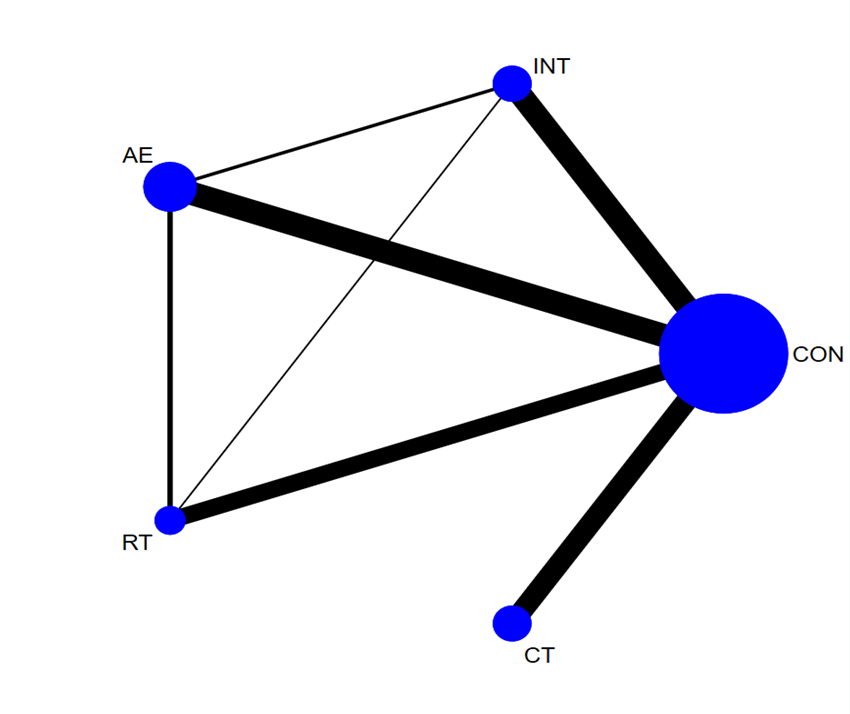


**Appendix** **13.1** Network of eligible comparisons for Pulse Wave Velocity.


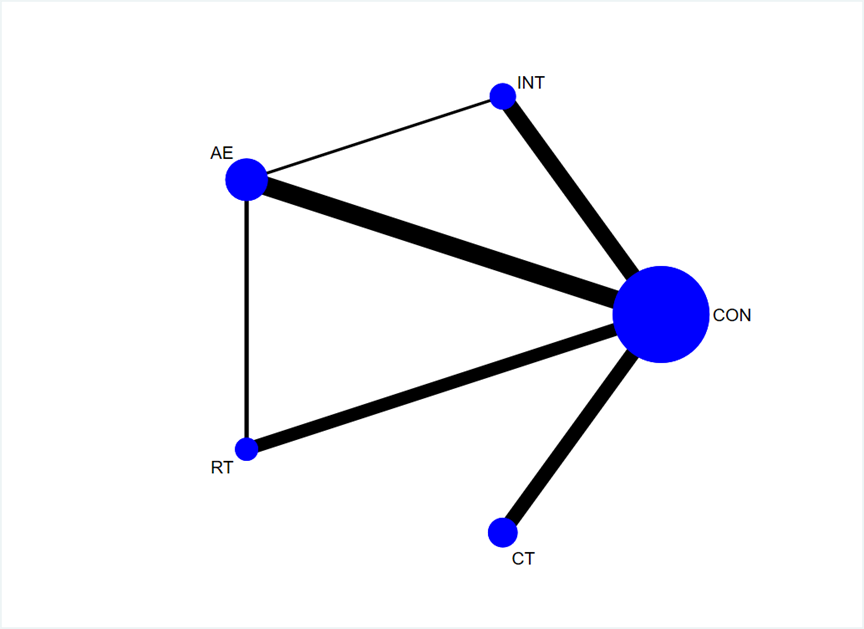


**Appendix 13.2** Network of eligible comparisons for Systolic Blood Pressure.


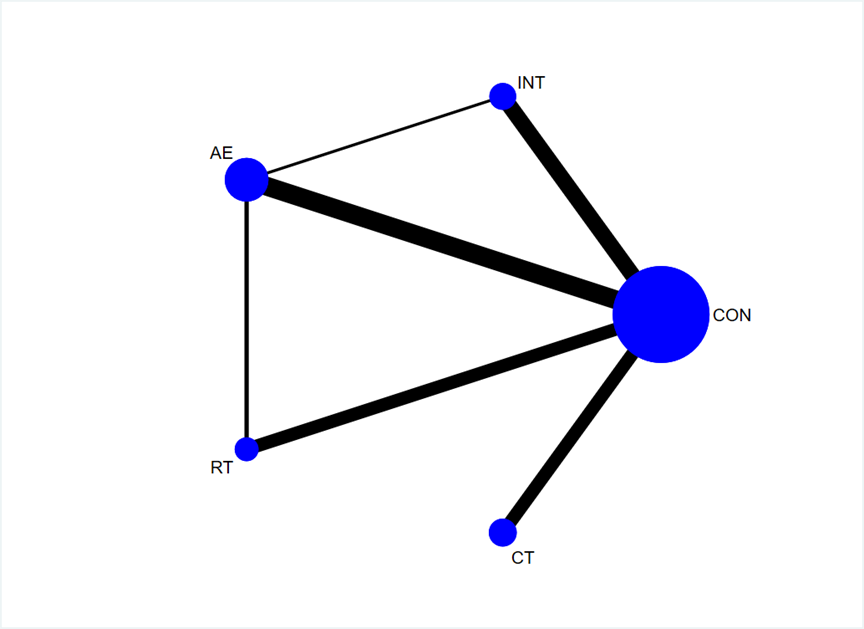


**Appendix** **13.3** Network of eligible comparisons for Diastolic Blood Pressure.

## Appendix 14. GRADE for Pulse Wave Velocity, Systolic Blood Pressure, and Diastolic Blood Pressure.

**Appendix 14.1** Summary of study limitations of the included studies.

The colors of the line then indicate the summative RoB assessment of each comparison based on the above information – low RoB comparison [green] and moderate RoB comparison [yellow]. There was no comparison which was judged to be at high RoB.


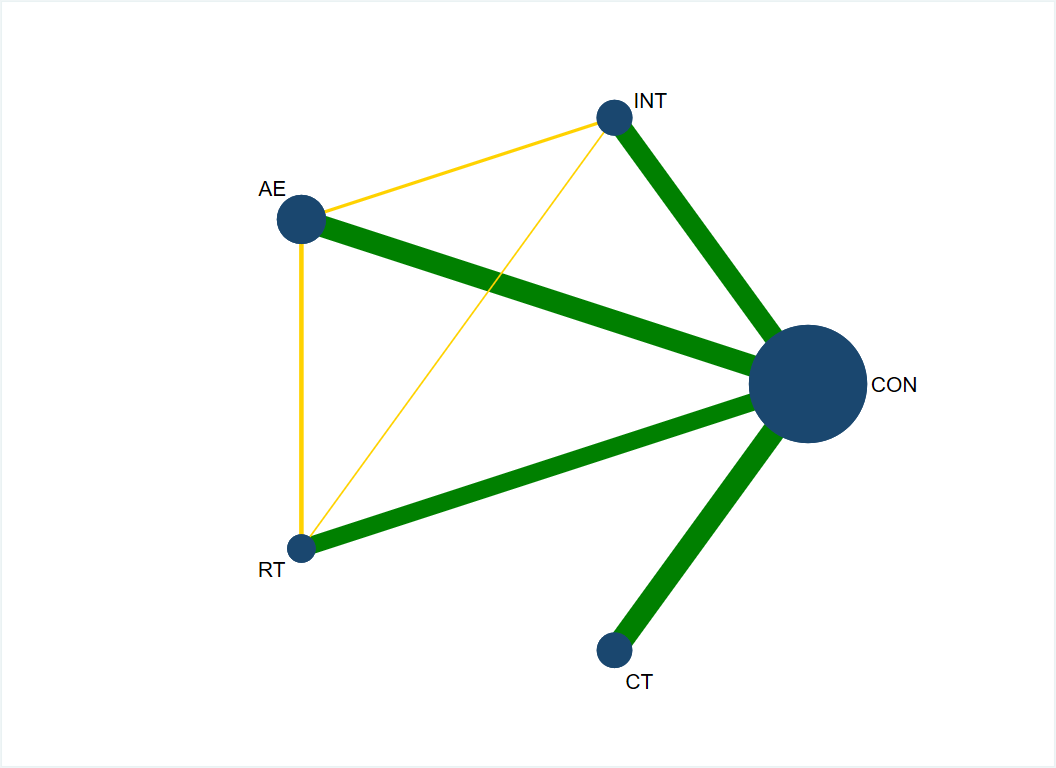


**Appendix** **14.1.1** Network of literature quality comparisons for Pulse Wave Velocity


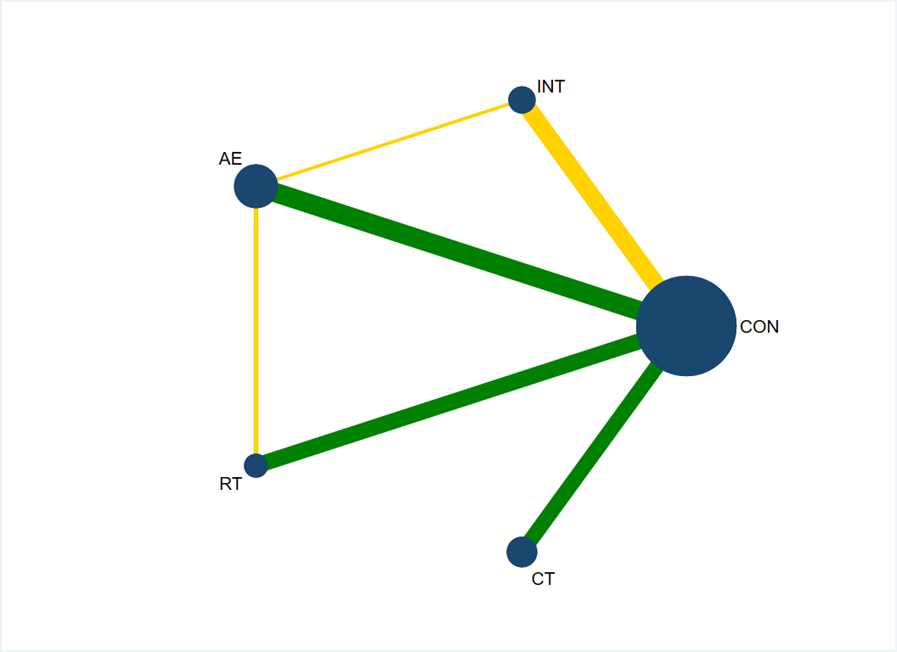
 **Appendix** **14.1.2** Network of literature quality comparisons for Systolic Blood Pressure


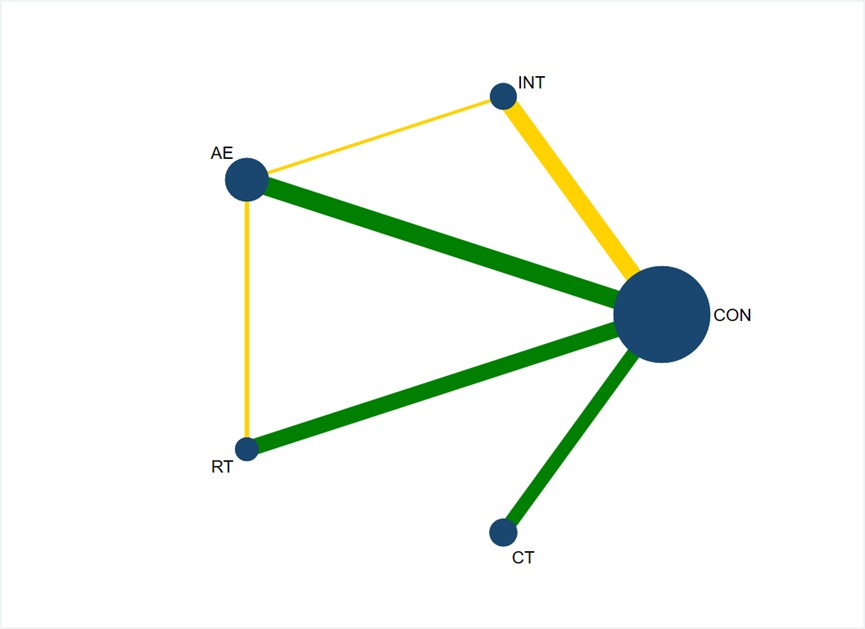


**Appendix** **14.1.3** Network of literature quality comparisons for Diastolic Blood Pressure

**Appendix 14.2** Contribution of ROB comparisons to each network estimate.

Based on the above assessment of RoB for each comparison and the contribution matrix detailing contribution of each direct comparison to all network estimates, the following bar graphs show the percentage of low or moderate RoB contributions for each network estimate.

The judgements about study limitations in each direct comparison is shown at the beginning of the graph.

Each bar corresponds to a NMA relative treatment effect and shows how much information comes from comparisons at low risk of bias [green] or moderate risk of bias [yellow].

*Note.* CON, control group; INT, interval training; AE, means aerobic exercise; RT , resistance training; CT , means combined training.


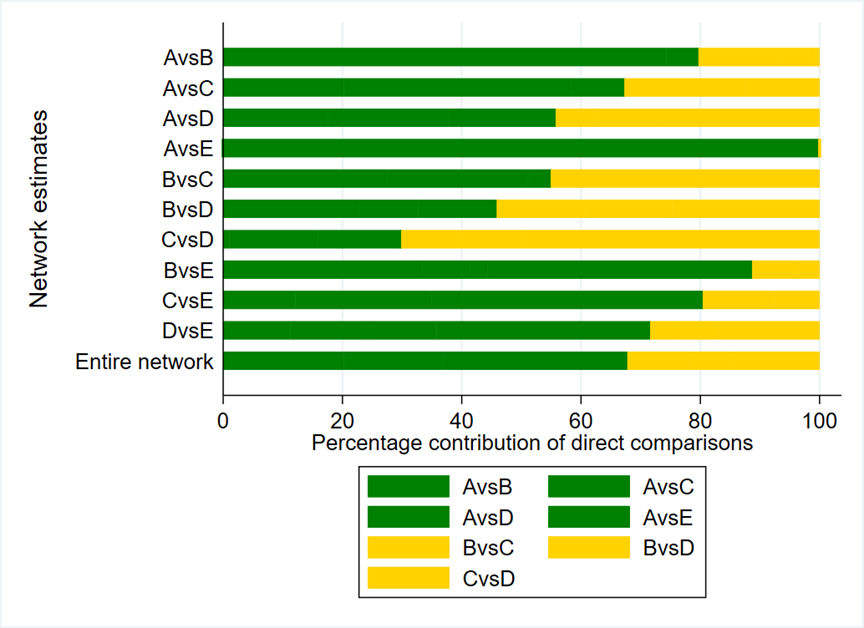


**Appendix** **14.2.1** Contribution of ROB comparisons to each network estimate for Pulse Wave Velocity.


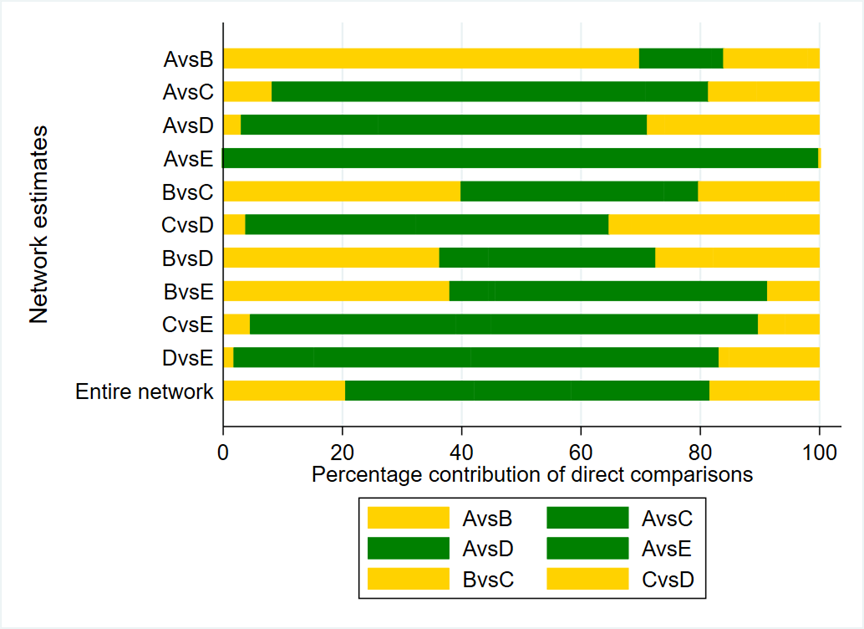


**Appendix 14.2.2** Contribution of ROB comparisons to each network estimate for Systolic Blood Pressure


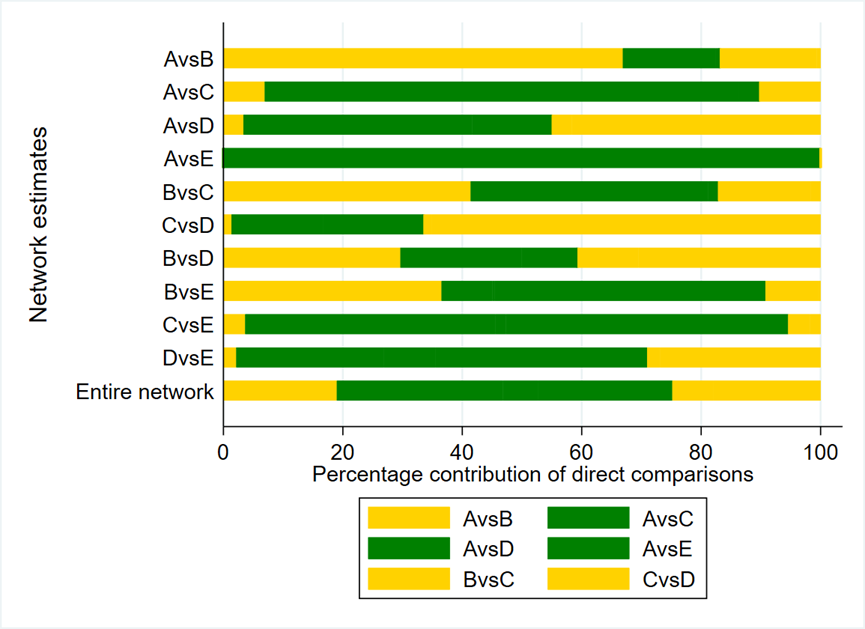


**Appendix 14.2.2** Contribution of ROB comparisons to each network estimate for

Diastolic Blood Pressure

**Appendix 14.3** Table of reasons for downgrading.

Based on all the above information, we GRADE each network estimate according to the following criteria.

1. **Study limitations**

Step 1, we summatively rated the overall RoB of each study as follow(low/moderate/high):

Studies were classified as having low risk of bias if none of the domains above was rated as high risk of bias and three or less were rated as unclear risk; moderate if one was as rated as high risk of bias or none was rated as high risk of bias but four or more were rated as unclear risk , and all other cases were assumed to pertain to high risk of bias.(Cipriani 2018)

Step 2, Different weights of 0/-1/-2 are assigned to low/moderate/high, respectively , and the grade of each mixed comparison (NMA) is calculated based on the evidence contribution plot , such as NMA grade CD = AB* weight1 + AC * weight2+ AD * weight3+ BC* weight4+ BD* weight5 + CD * weight6

Step 3, Downgrade according to the calculation results of step 2, less than -1 downgraded by one level, less than -2 downgraded by two levels. (Salanti2014)

1. **Indirectness**

First, we judged whether the results of each subgroup in the subgroup analysis were consistent, and the inconsistency should be downgraded by one level, and the consistency should not be downgraded (No subgroups No demote required); Secondly, the results in the contribution graph only comes from indirect comparisons and needs to be downgraded by one level; Third, sorting does not consider downgrade.

1. **Inconsistency**

Heterogeneity was judged by looking at the prediction interval plot , and downgraded by one level when there was a significant difference between the fixed-effect model and the random-effects model , and no downgrade when there was no significant difference；Inconsistencies are judged by the p-value of Loop specific and Node splitting , which need to be downgraded one level when the p-value < 0.05 , and not by a level when the p-value > 0.05；The consistency of the sorting results is judged by the p-value of Global inconsistency, which needs to be downgraded one level when the p-value < 0.05, and not by a level when the p-value > 0.05.

1. **Imprecision**

First, imprecision is assessed by sample size, which is downgraded by one level when the sample size < 400, and not downgraded when the sample size > 400.Second, assess the imprecision of ranking results based on the gap between the ranking values. If the gap between the rank values for each treatment is small, indicating poor stability, it is downgraded by one level (Salanti et al ,2014)

**(5) Publication bias**

Publication bias is judged by observing the comparison-adjusted funnel plot, which need to be downgraded by one level when the number of files on both sides of the vertical line is unevenly distributed.

**Appendix 14.3.1** Summary of our confidence in effect estimates and ranking of treatments.

| **Comparison** | **Nature**  **of the evidence** | **Confidence** | **Downgrading due to** |
| --- | --- | --- | --- |
| Pulse Wave Velocity | | | |
| AB: | Mixed | Low | Inconsistency^6^; Publication bias^5^ |
| AC: | Mixed | High |  |
| AD: | Mixed | High |  |
| AE: | Mixed | Moderate | Inconsistency^6^ |
| BC: | Mixed | Moderate | Study limitations^1^ |
| BD: | Mixed | Moderate | Study limitations^1^ |
| CD: | Mixed | Low | Study limitations^1^; Publication bias^5^ |
| BE: | Indirect | Moderate | Indirectness^2^ |
| CE: | Indirect | Moderate | Indirectness^2^ |
| DE: | Indirect | Moderate | Indirectness^2^ |
| *Ranking of treatments* |  | High |  |
| Systolic Blood Pressure | | | |
| AB: | Mixed | Low | Study limitations^1^; Publication bias^5^ |
| AC: | Mixed | High |  |
| AD: | Mixed | High |  |
| AE: | Mixed | Moderate | Inconsistency^6^ |
| BC: | Mixed | Low | Study limitations^1^; Imprecision^4^ |
| CD: | Mixed | High |  |
| BD: | Indirect | Low | Study limitations^1^; Indirectness^2^ |
| BE: | Indirect | Low | Indirectness^2^; Inconsistency^6^ |
| CE: | Indirect | Moderate | Indirectness^2^ |
| DE: | Indirect | Moderate | Indirectness^2^ |
| *Ranking of treatments* |  | High |  |
| Diastolic Blood Pressure | | | |
| AB: | Mixed | Moderate | Study limitations^1^ |
| AC: | Mixed | Moderate | Publication bias^5^ |
| AD: | Mixed | Moderate | Publication bias^5^ |
| AE: | Mixed | High |  |
| BC: | Mixed | Moderate | Study limitations^1^ |
| CD: | Mixed | Moderate | Study limitations^1^ |
| BD: | Indirect | Low | Study limitations^1^; Indirectness^2^ |
| BE: | Indirect | Moderate | Indirectness^2^ |
| CE: | Indirect | Moderate | Indirectness^2^ |
| DE: | Indirect | Moderate | Indirectness^2^ |
| *Ranking of treatments* |  | High |  |

^1^Dominated by evidence at high or moderate risk of bias.

^2^No convincing evidence for the plausibility of the transitivity assumption.

^3^Predictive intervals for treatment effect include effects that would have different interpretations (there is additionally no convincing evidence for the plausibility of the transitivity assumption).

^4^Confidence intervals include values favouring either treatment.

^5^60% of the information is from studies at moderate risk of bias.

^6^Moderate level of heterogeneity, and some evidence of inconsistency in the network.

*Note.* A means control group; B means interval training; C means aerobic exercise; D means resistance training; E means combined training.

##

## Appendix 15. Subgroup analysis for Pulse Wave Velocity in moderate intensity group.

**
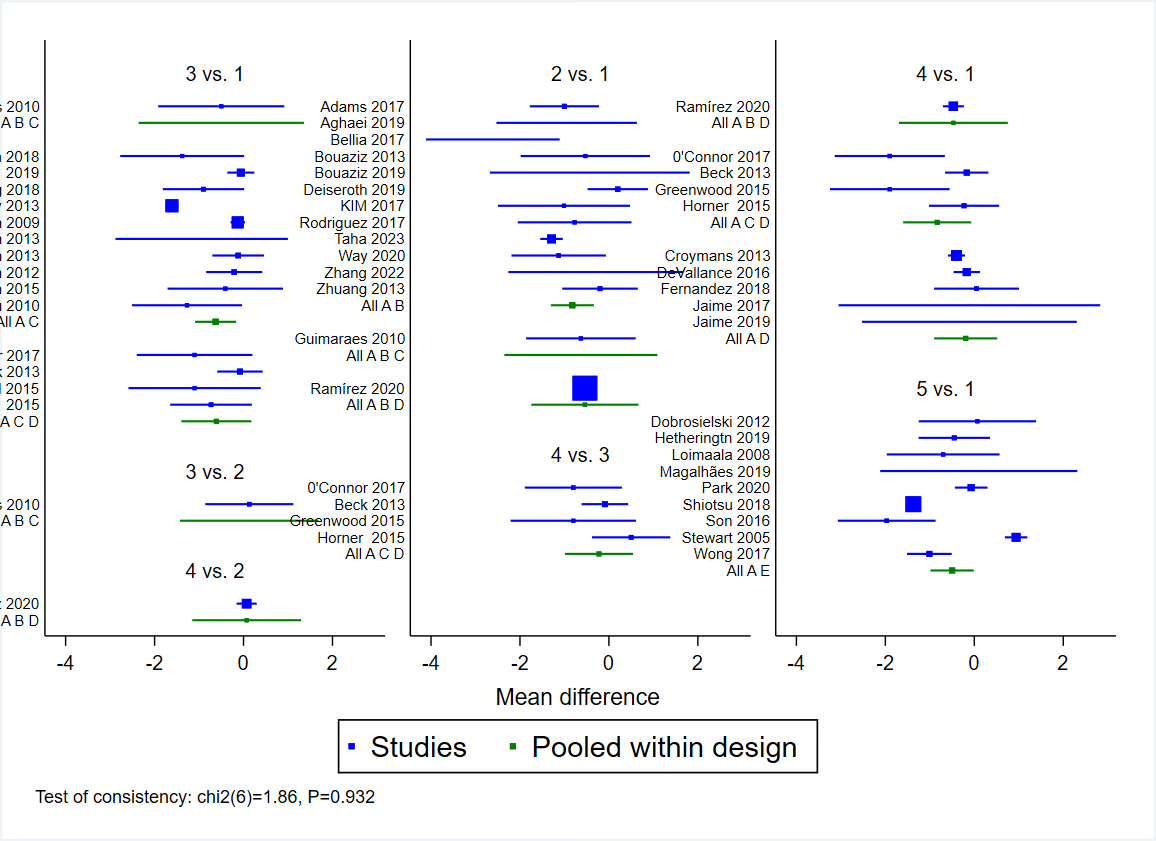
**

**Appendix** **15.1** Forest plot of pairwise meta-analysis for pulse wave velocity

Note. 1 means interval training; 2 means aerobic exercise; 3 means resistance exercise; 4 means combined training; 5 means control group.


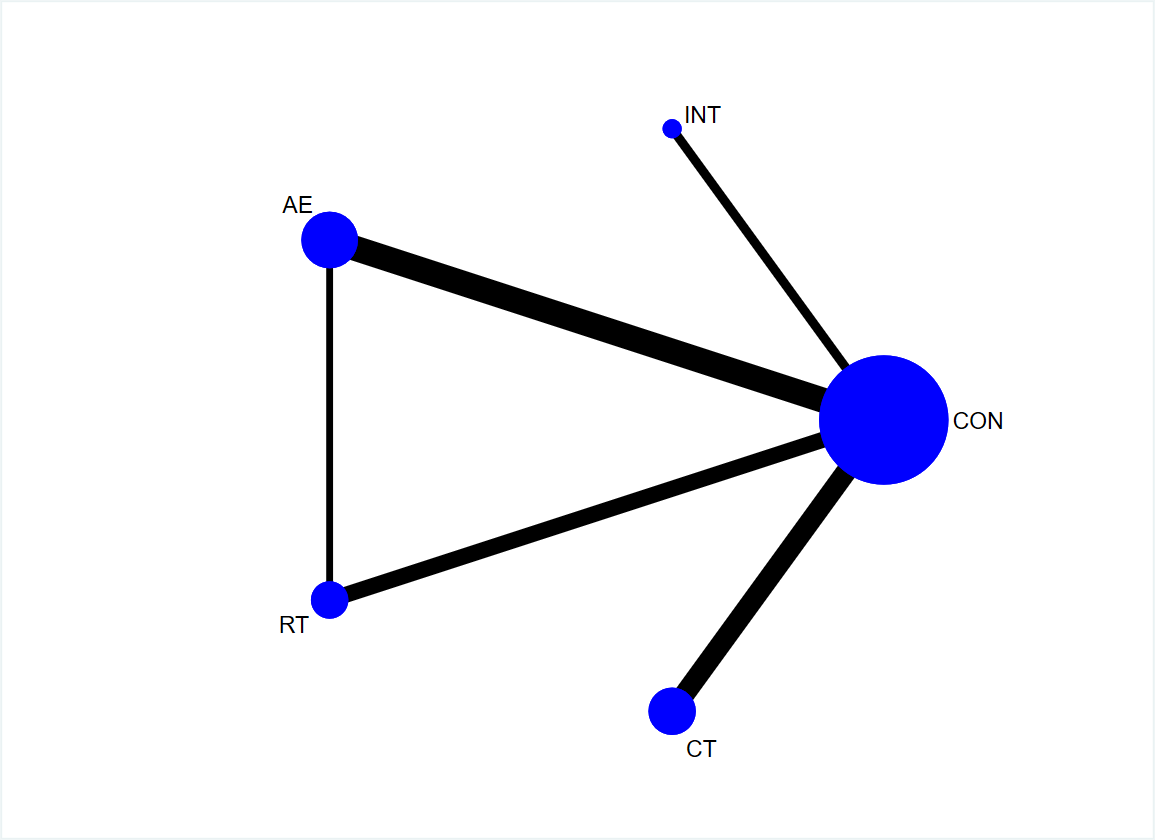


**Appendix** **15.2** Network of eligible comparisons for pulse wave velocity

***Global inconsistency test***

**χ^2^ = 2.85**

***p* = 0.2411**

No evidence for the existence of significant global inconsistency

***Local inconsistency test***

**
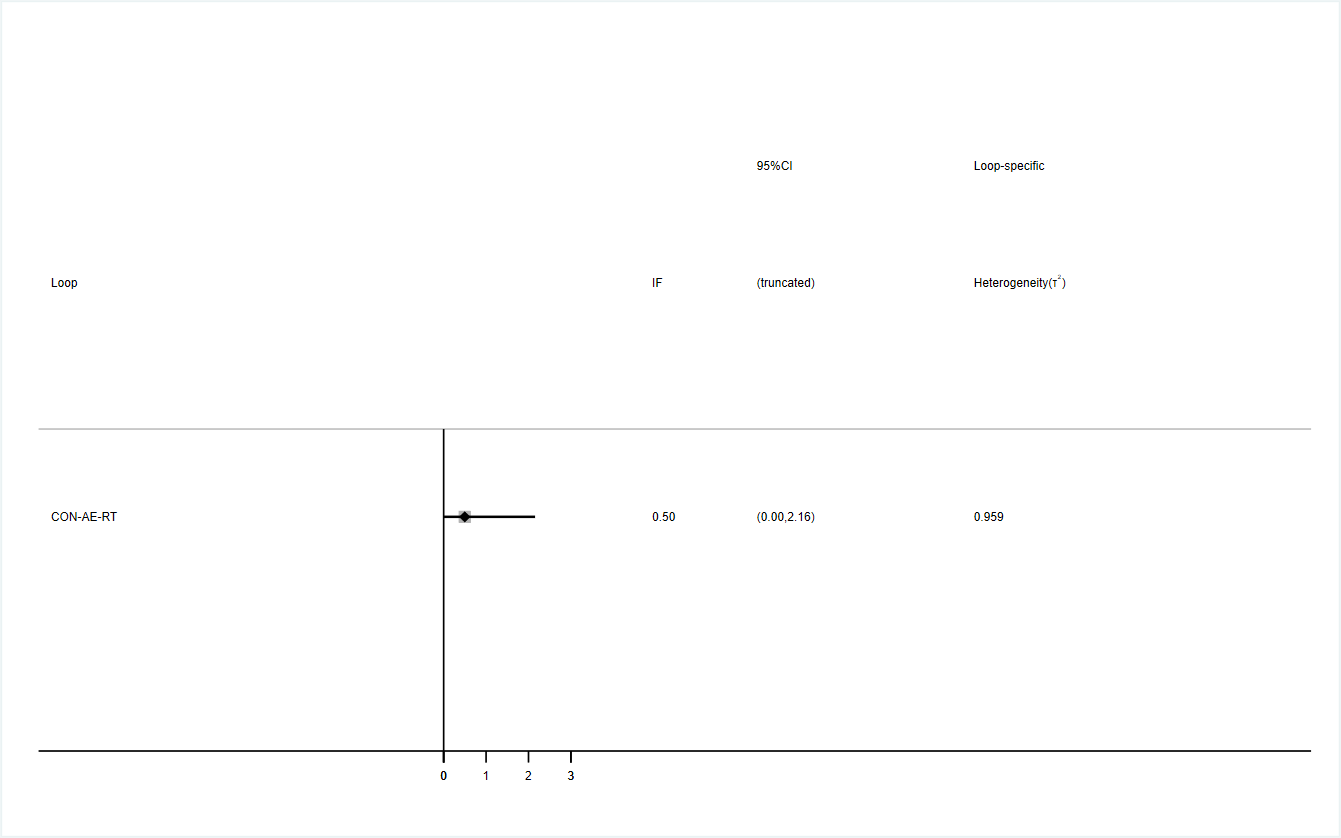
**

**Appendix 15.3** Loop-specific heterogeneity for pulse wave velocity.

Note. CON means non-exercise control: AE means aerobic exercise; RT means resistance training; INT means interval training.

**Appendix** **15.4** Local inconsistency test

| Side | Direct  Coef. | Std. Err. | Indirect  Coef. | Std. Err. | Difference  Coef. | Std.Err. | P>z |
| --- | --- | --- | --- | --- | --- | --- | --- |
| A C | -0.454 | 0.277 | 1.782 | 1.280 | -2.236 | 1.310 | 0.088 |
| A D | 0.021 | 0.361 | -0.377 | 1.166 | 0.398 | 1.223 | 0.745 |
| C D | -0.086 | 0.527 | 0.838 | 0.567 | -0.925 | 0.774 | 0.232 |


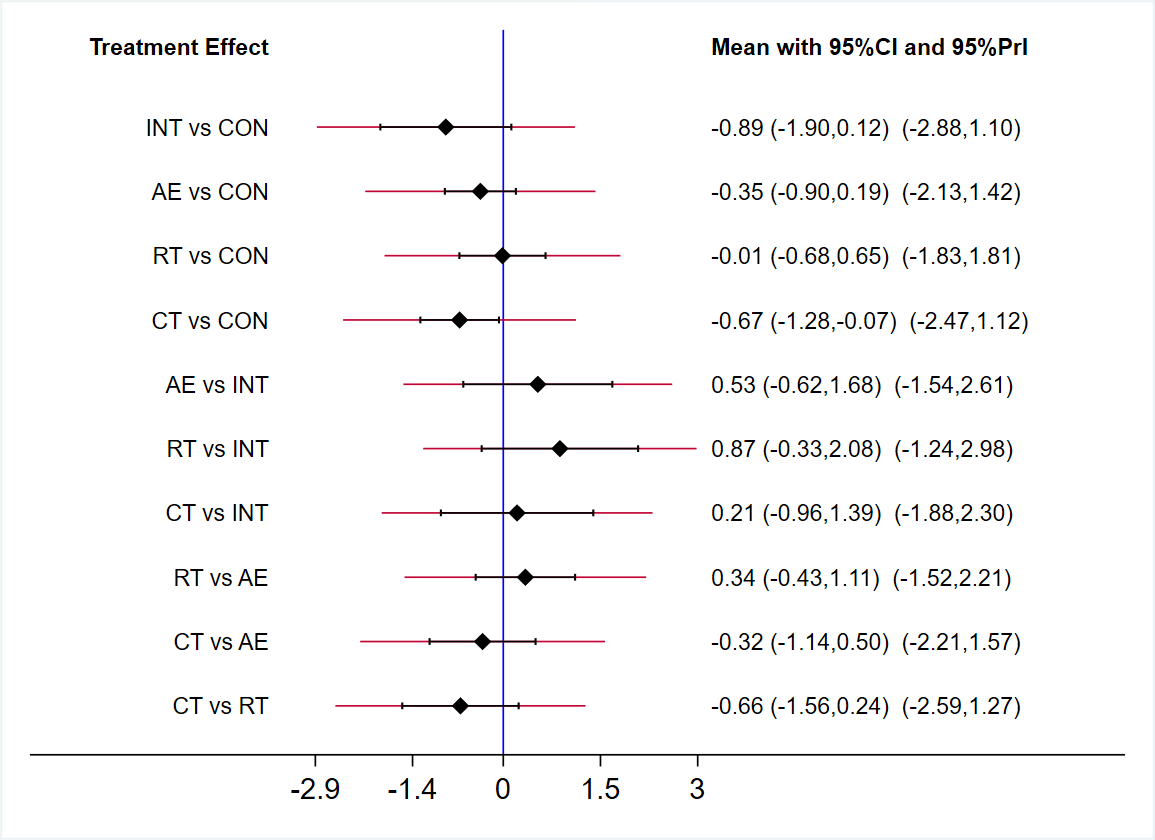


**Appendix** **15.5** Interval plot of network meta-analysis for pulse wave velocity

**
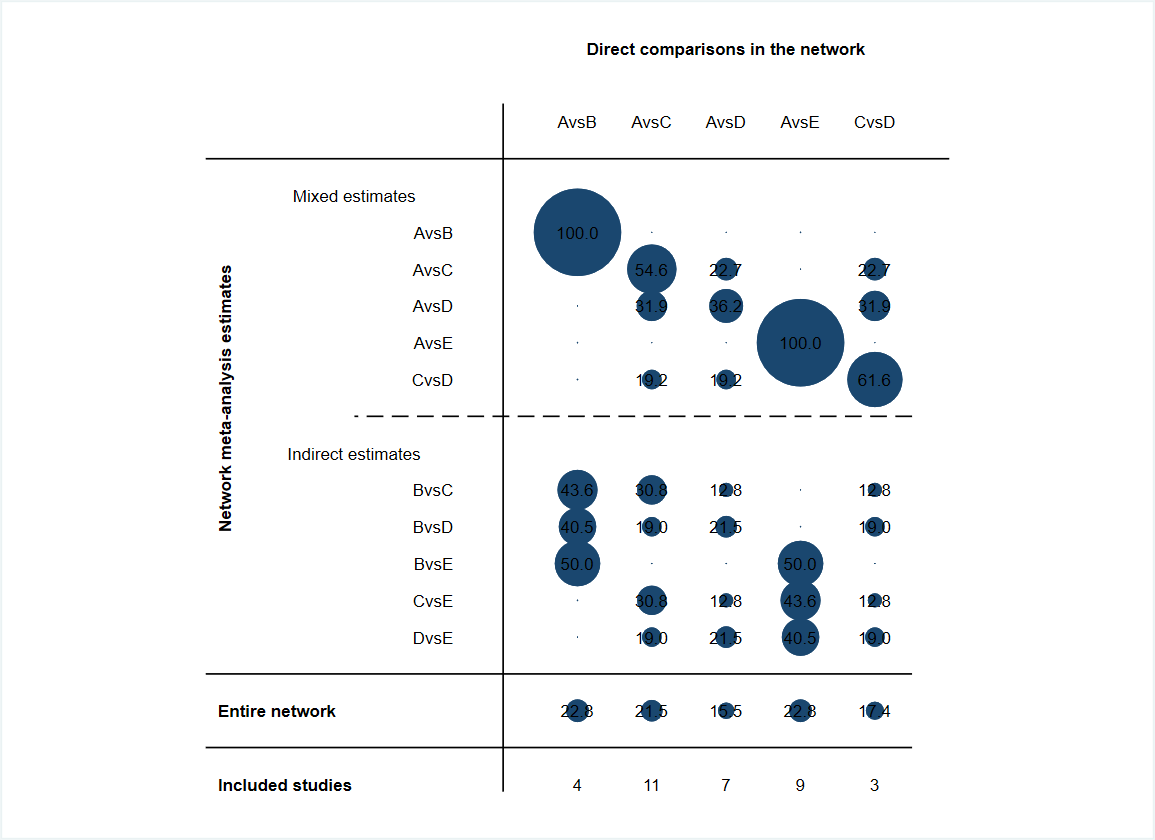
**

**Appendix** **15.6** Contribution plot for pulse wave velocity.

*Note.* A means control group; B means interval training; C means aerobic exercise; D means resistance training; E means combined training.


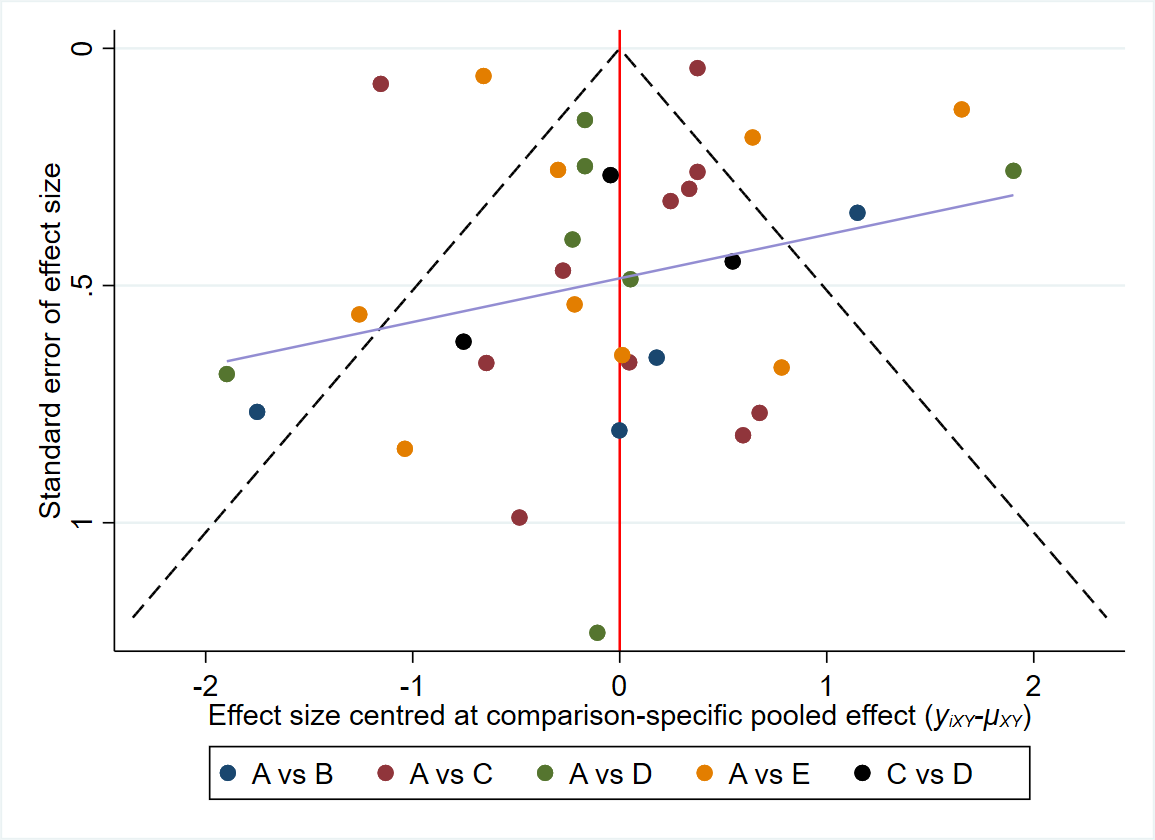


**Appendix 15.7** Comparison-adjusted funnel plot for pulse wave velocity

*Note.* A means control group; B means interval training; C means aerobic exercise; D means resistance training; E means combined training.


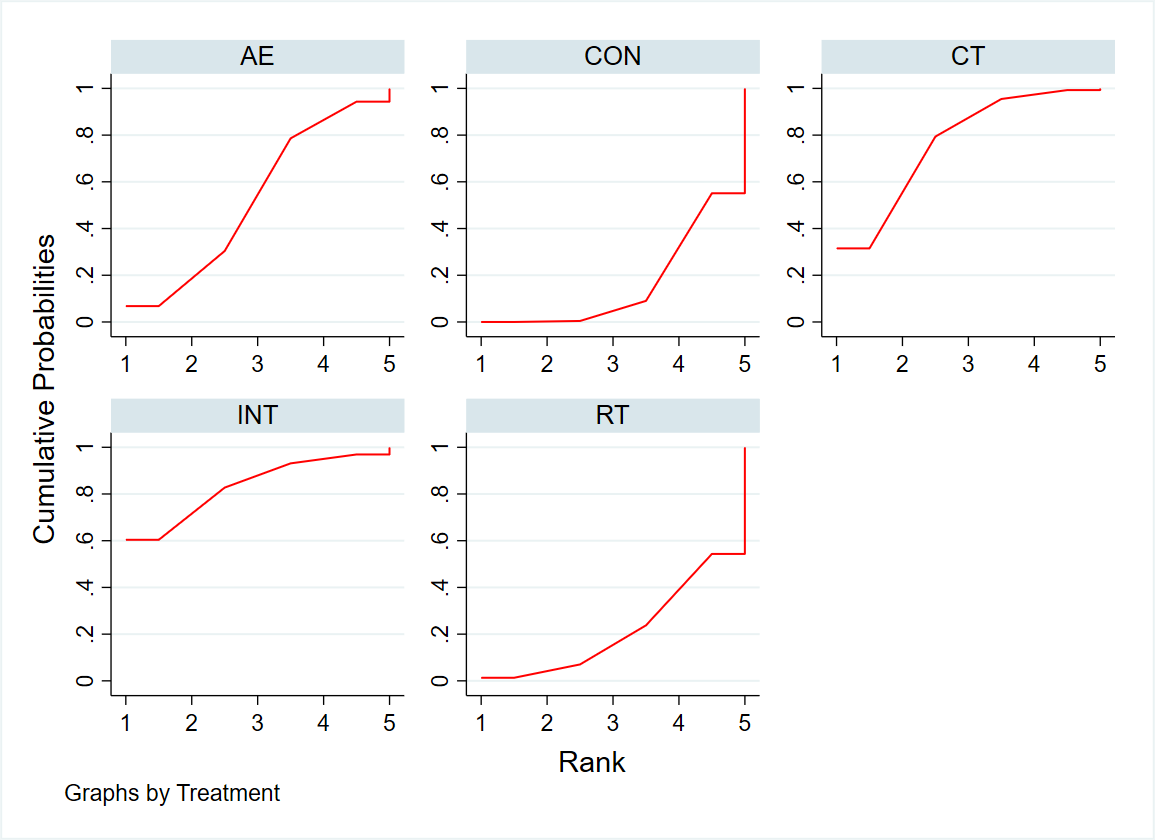


**Appendix 15.8** Ranking of the effects of different types of exercise on pulse wave velocity

**Appendix 15.9** The ranking of effects of different exercise on pulse wave velocity

| Treatment | SUCRA | Mean Rank | Probability of Best (%) |
| --- | --- | --- | --- |
| CON | 16.1 | 0 | 4.4 |
| INT | 83.3 | 60.4 | 1.7 |
| AE | 52.5 | 6.8 | 2.9 |
| RT | 21.6 | 1.3 | 4.1 |
| CT | 76.4 | 31.5 | 1.9 |

Note. CON means non-exercise control; INT means interval training; AE means aerobic exercise; RT means resistance training; CT, means combined training

**Appendix** **15.10** Network meta-analysis matrix of PWV.

| CT | 0.66 (-0.24 ,1.56) | 0.32 (-0.50 ,1.14) | -0.21 (-1.39 ,0.96) | **0.67 (0.07 ,1.28)** |
| --- | --- | --- | --- | --- |
| -0.66 (-1.56 ,0.24) | RT | -0.34 (-1.11 ,0.43) | -0.87 (-2.08 ,0.33) | 0.01 (-0.65 ,0.68) |
| -0.32 (-1.14 ,0.50) | 0.34 (-0.43 ,1.11) | AE | -0.53 (-1.68 ,0.62) | 0.35 (-0.19 ,0.90) |
| 0.21 (-0.96 ,1.39) | 0.87 (-0.33 ,2.08) | 0.53 (-0.62 ,1.68) | INT | 0.89 (-0.12 ,1.90) |
| **-0.67 (-1.28, -0.07)** | -0.01 (-0.68 ,0.65) | -0.35 (-0.90 ,0.19) | -0.89 (-1.90 ,0.12) | CON |

## Appendix 16. Subgroup analysis for Pulse Wave Velocity in high intensity group.

**
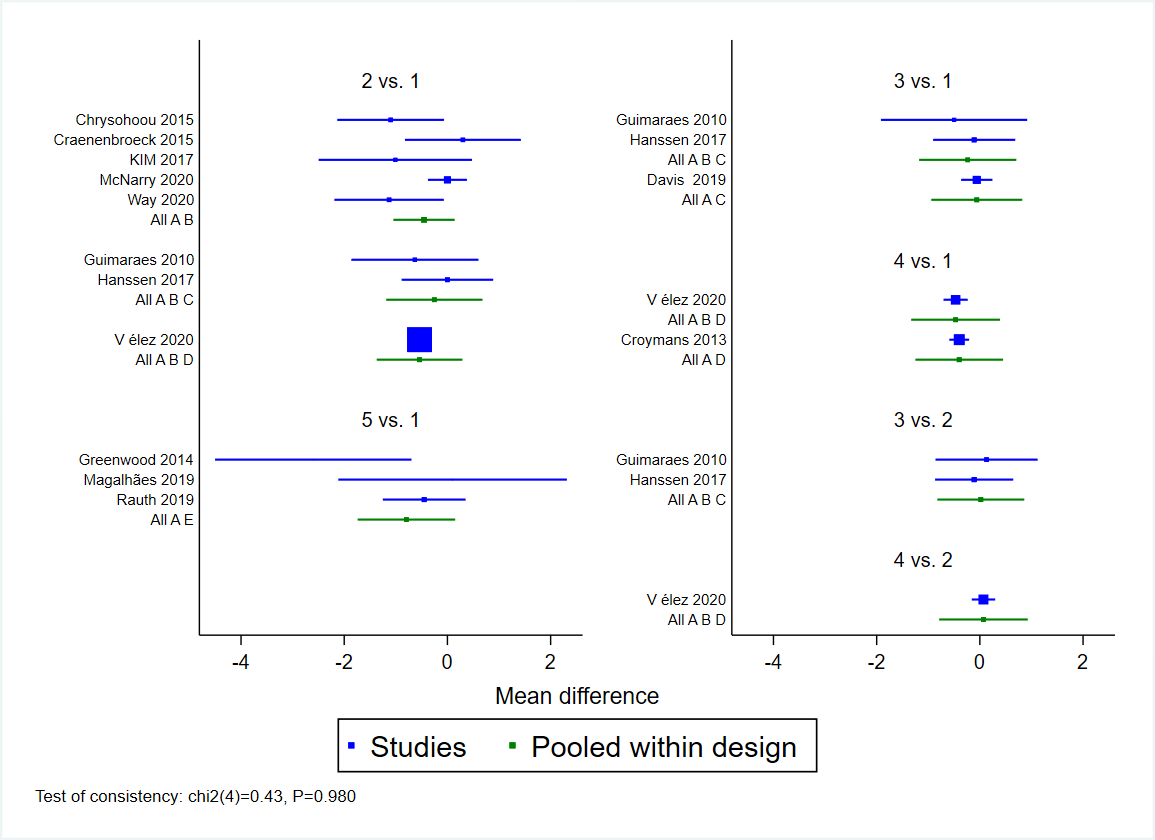
**

**Appendix 16.1** Forest plot of pairwise meta-analysis for pulse wave velocity

Note. 1 means interval training; 2 means aerobic exercise; 3 means resistance exercise; 4 means combined training; 5 means control group.


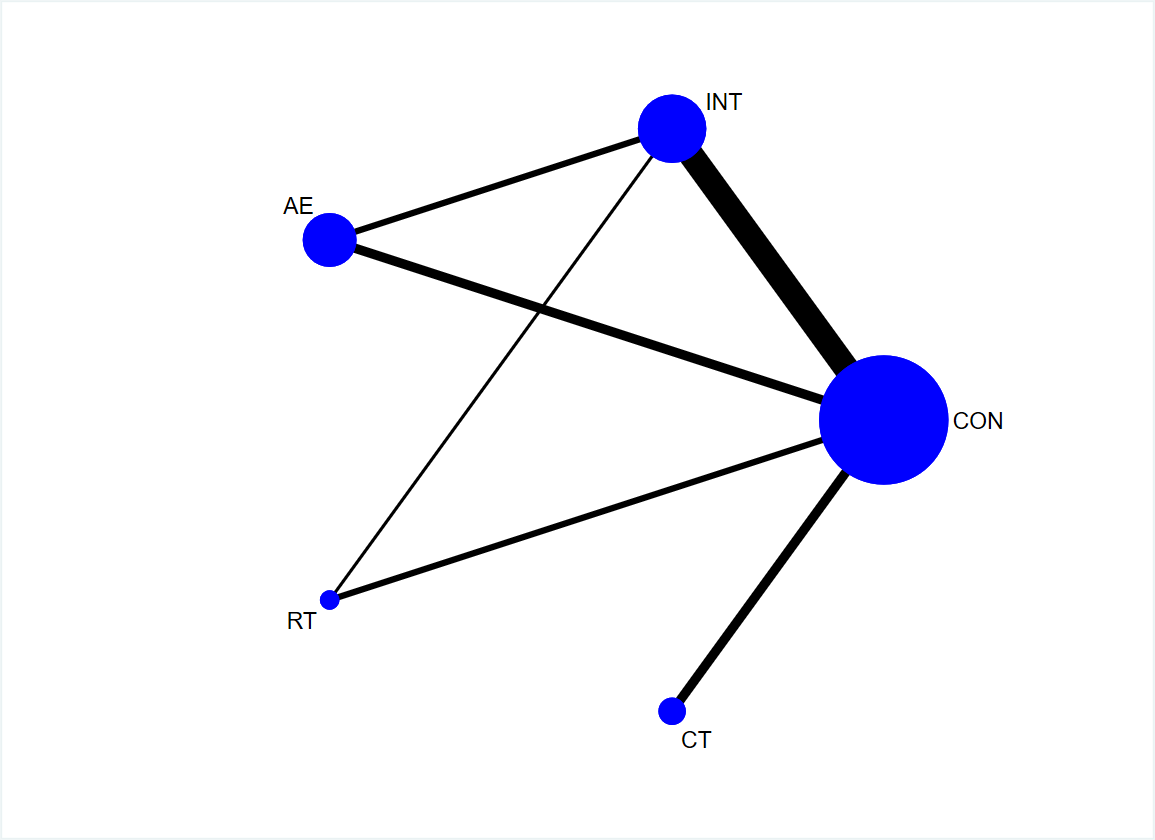


**Appendix** **16.2** Network of eligible comparisons for pulse wave velocity

***Global inconsistency test***

**χ^2^ = 0.43**

***p* = 0.98**

No evidence for the existence of significant global inconsistency

***Local inconsistency test***

**
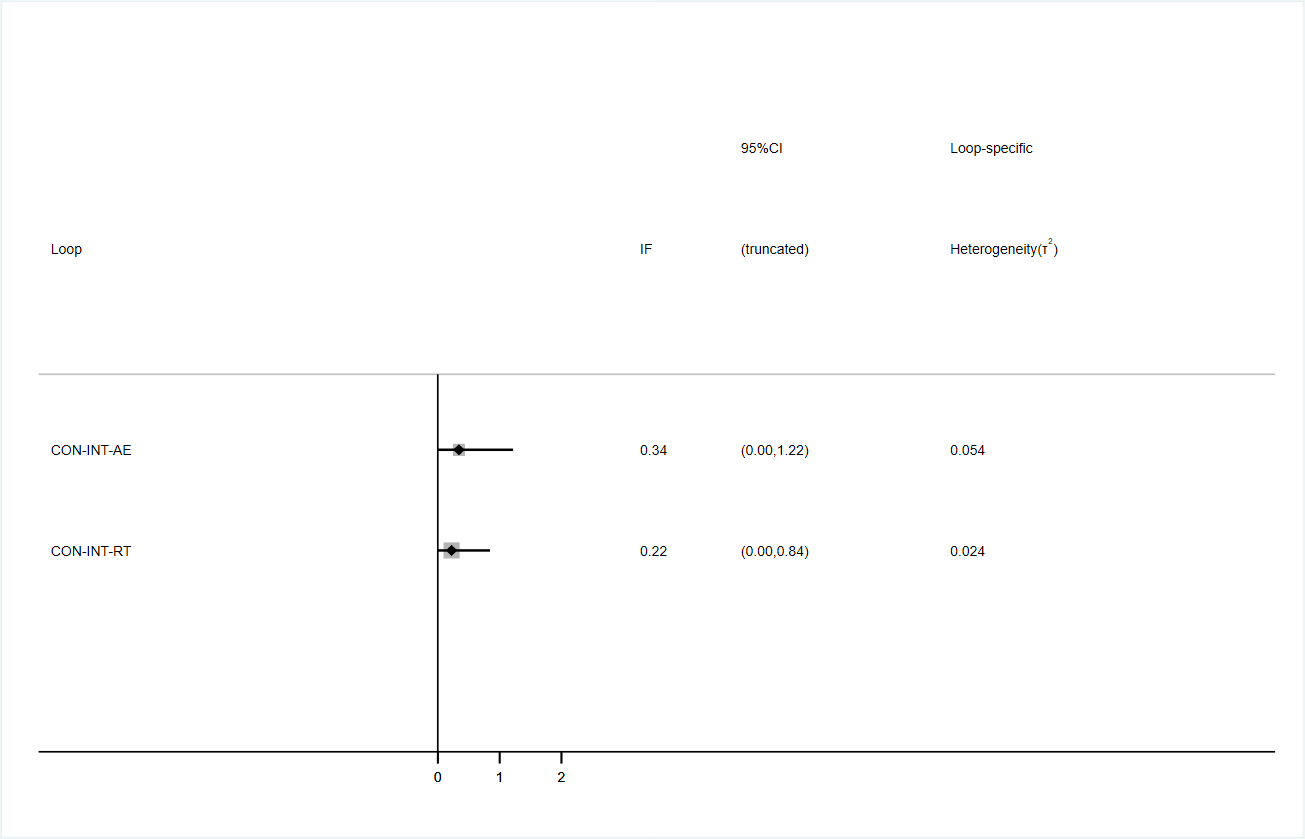
**

**Appendix 16.3** Loop-specific heterogeneity for pulse wave velocity.

Note. CON means non-exercise control; AE means aerobic exercise; RT means resistance training; INT means interval training.

**Appendix 16.4** Local inconsistency test

| Side | Direct  Coef. | Std. Err. | Indirect  Coef. | Std. Err. | Difference  Coef. | Std.Err. | P>z |
| --- | --- | --- | --- | --- | --- | --- | --- |
| A B | -0.408 | 0.144 | -0.196 | 0.519 | -0.212 | 0.543 | 0.696 |
| A C | -0.102 | 0.199 | -0.654 | 0.652 | 0.552 | 0.699 | 0.430 |
| A D | -0.428 | 0.076 | 0.237 | 0.297 | -0.665 | 0.290 | 0.022 |
| B C | 0.017 | 0.330 | 0.378 | 0.265 | -0.361 | 0.424 | 0.394 |
| B D | 0.094 | 0.215 | -0.153 | 0.268 | 0.247 | 0.345 | 0.475 |


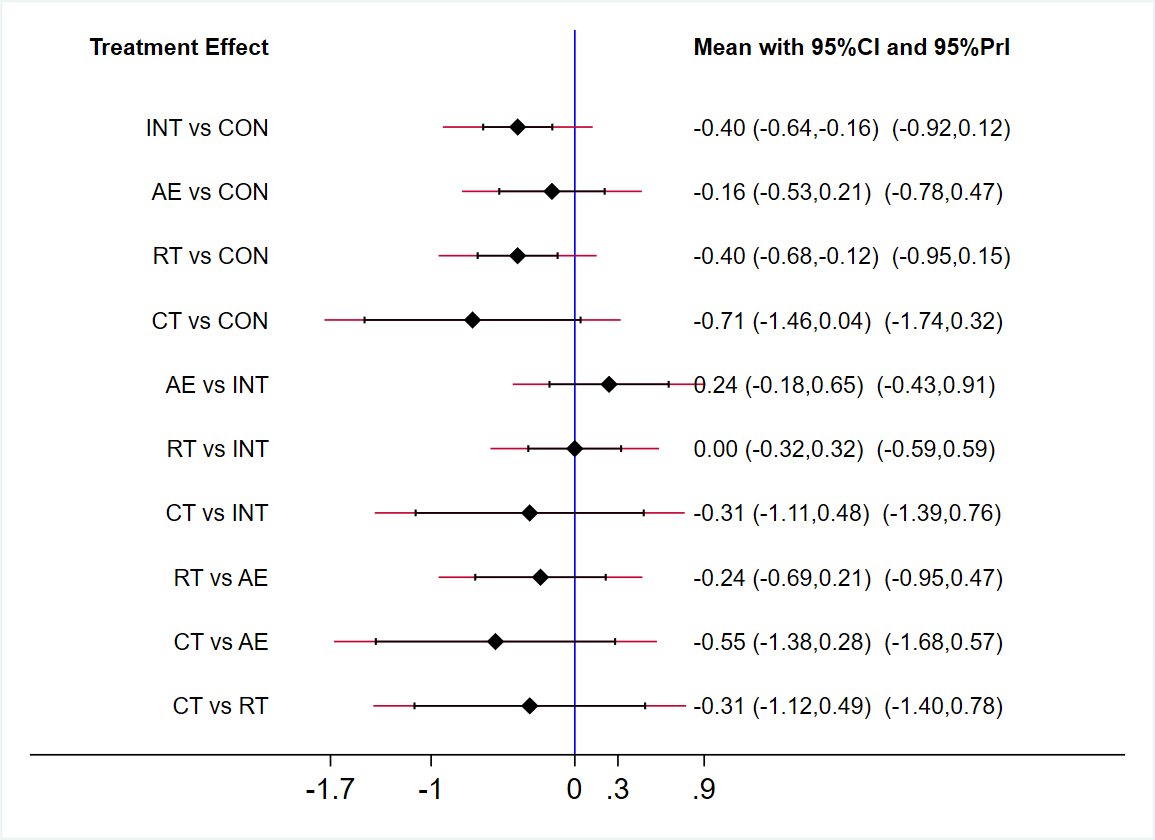


**Appendix** **16.5** Interval plot of network meta-analysis for pulse wave velocity

**
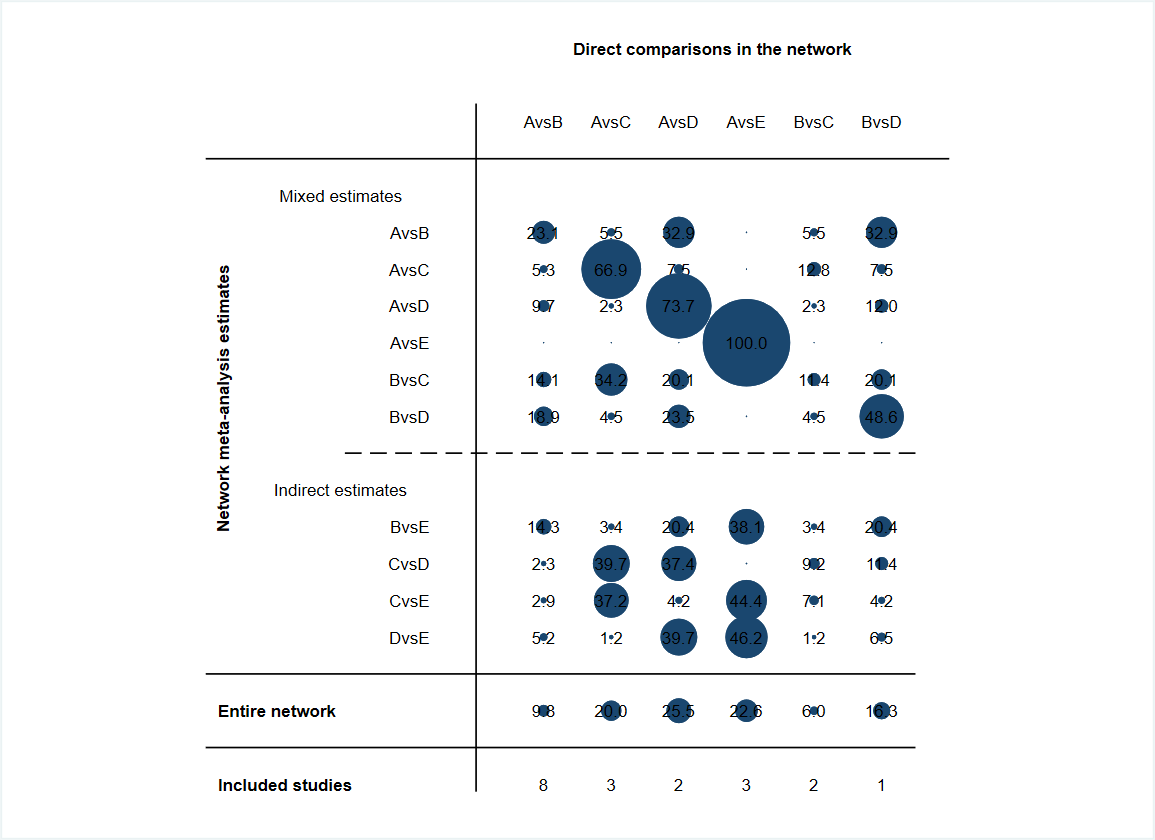
**

**Appendix** **16.6** Contribution plot for pulse wave velocity.

*Note.* A means control group; B means interval training; C means aerobic exercise; D means resistance training; E means combined training.


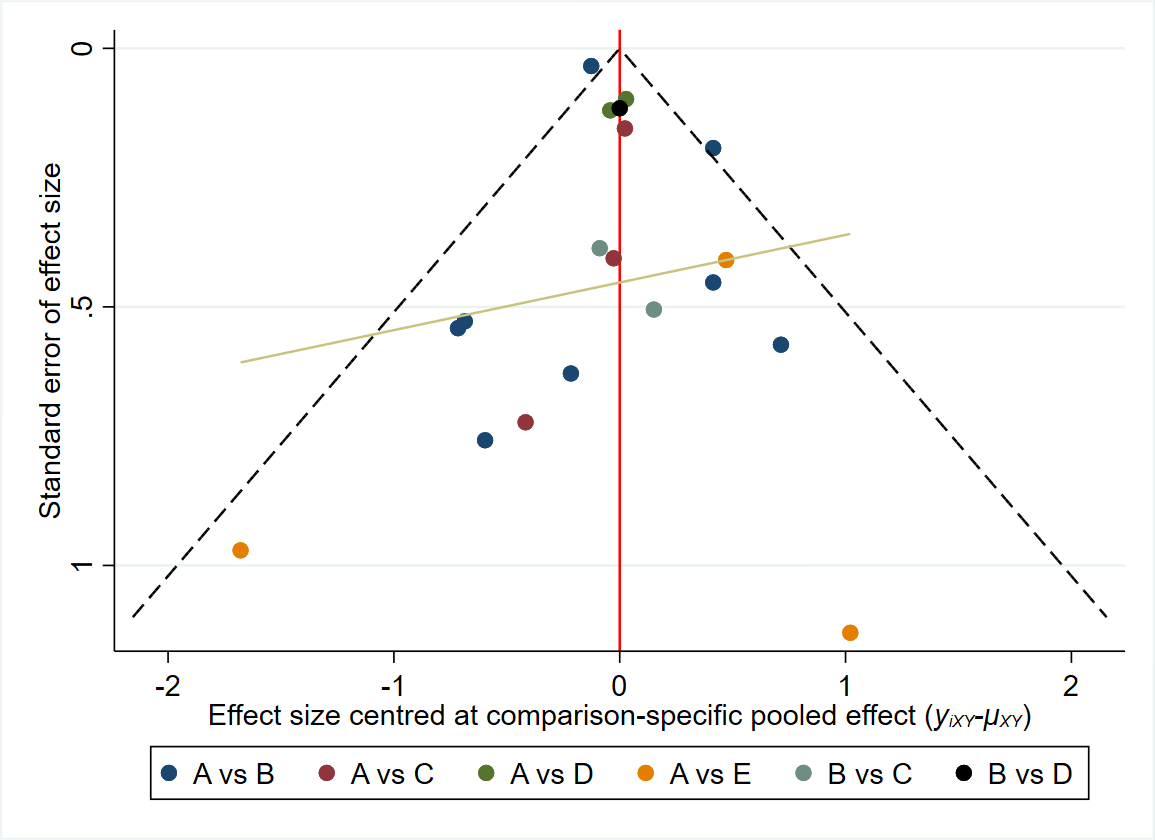


**Appendix 16.7** Comparison-adjusted funnel plot for pulse wave velocity

*Note.* A means control group; B means interval training; C means aerobic exercise; D means resistance training; E means combined training.


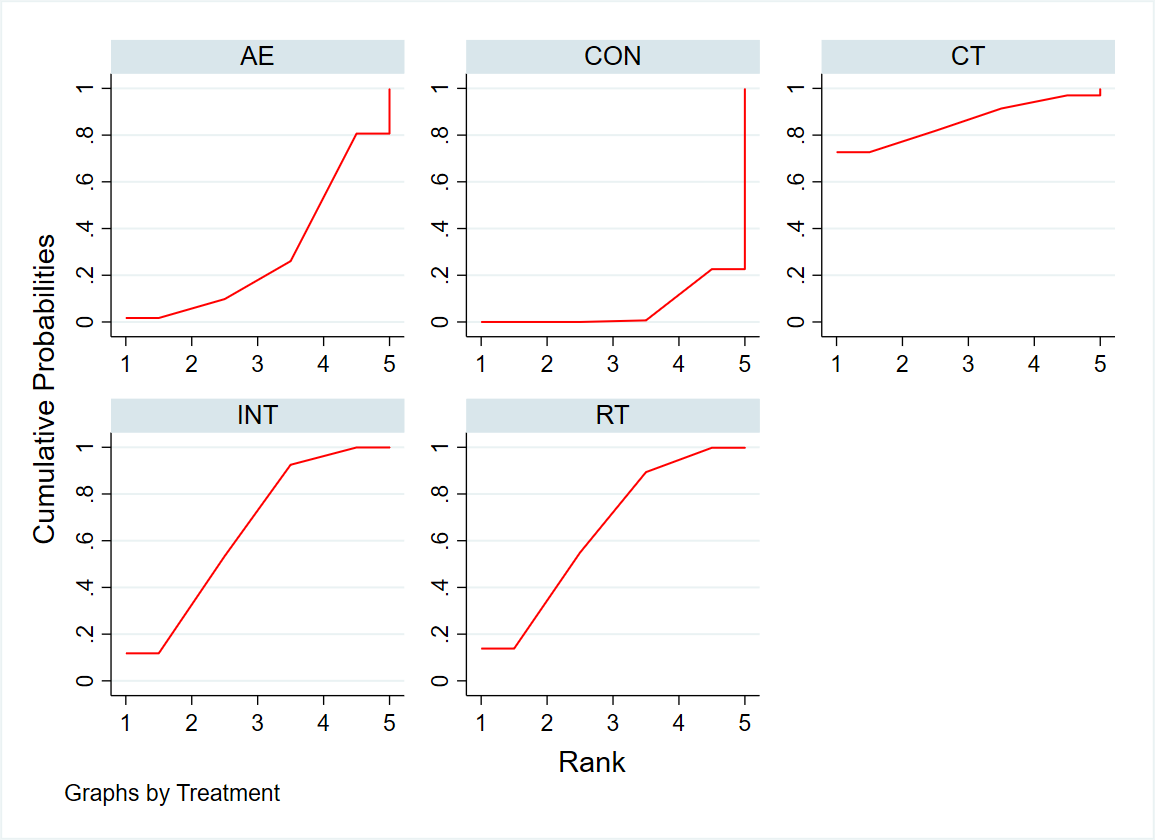


**Appendix 16.8** Ranking of the effects of different types of exercise on pulse wave velocity

**Appendix** **16.9** The ranking of effects of different exercise on pulse wave velocity

| Treatment | SUCRA | Mean Rank | Probability of Best (%) |
| --- | --- | --- | --- |
| Control group | 5.8 | 0 | 4.8 |
| Interval training | 64.4 | 11.8 | 2.4 |
| Aerobic exercise | 29.5 | 1.7 | 3.8 |
| Resistance training | 64.5 | 13.8 | 2.4 |
| Combined training | 85.7 | 72.7 | 1.6 |

Note. CON means non-exercise control; INT means interval training; AE means aerobic exercise; RT means resistance training; CT, means combined training

**Appendix 16.10** Network meta-analysis matrix of PWV

| CT | 0.31 (-0.49 ,1.12) | 0.55 (-0.28 ,1.38) | 0.31 (-0.48 ,1.11) | 0.71 (-0.04 ,1.46) |
| --- | --- | --- | --- | --- |
| -0.31 (-1.12 ,0.49) | RT | 0.24 (-0.21 ,0.69) | 0.00 (-0.32 ,0.32) | **0.40 (0.12 ,0.68)** |
| -0.55 (-1.38 ,0.28) | -0.24 (-0.69 ,0.21) | AE | -0.24 (-0.65 ,0.18) | 0.16 (-0.21 ,0.53) |
| -0.31 (-1.11 ,0.48) | -0.00 (-0.32 ,0.32) | 0.24 (-0.18 ,0.65) | INT | **0.40 (0.16 ,0.64)** |
| -0.71 (-1.46 ,0.04) | **-0.40 (-0.68, -0.12)** | -0.16 (-0.53 ,0.21) | **-0.40 (-0.64, -0.16)** | CON |

## Appendix 17. List of included studies.

1. Guimarães GV, Ciolac EG, Carvalho VO, D'Avila VM, Bortolotto LA, Bocchi EA. Effects of continuous vs. interval exercise training on blood pressure and arterial stiffness in treated hypertension. Hypertens Res. 2010; 33(6): 627-632. <https://doi.org/10.1038/hr.2010.42>.
2. Bouaziz W, Lang P-O, Schmitt E, Leprêtre P-M, Lefebvre F, Momas C*,*  Effects of a short-term interval aerobic training program with recovery bouts on vascular function in sedentary aged 70 or over: A randomized controlled trial. Arch Gerontol Geriatr. 2019; 82: 217-225. <https://doi.org/10.1016/j.archger.2019.02.017>.
3. Aghaei Bahmanbeglou N, Ebrahim K, Maleki M, Nikpajouh A, Ahmadizad S. Short-Duration High-Intensity Interval Exercise Training Is More Effective Than Long Duration for Blood Pressure and Arterial Stiffness But Not for Inflammatory Markers and Lipid Profiles in Patients With Stage 1 Hypertension. J Cardiopulm Rehabil Prev. 2019; 39(1). <https://doi.org/10.1097/HCR.0000000000000377>.
4. Zhuang X , Xu H, Zheng J, Effect of high intensity interval training on vascular wall elasticity in the elderly. 2023; 45(05): 429-431. https:// doi.org/10.3760/cma.j.issn.0254-1424.2023.05.009.
5. Mora‐Rodriguez R, Ramirez‐Jimenez M, Fernandez‐Elias VE, Guio de Prada MV, Morales‐Palomo F, Pallares JG*,*  Effects of aerobic interval training on arterial stiffness and microvascular function in patients with metabolic syndrome. J Clin Hypertens. 2018; 20(1): 11-18. <https://doi.org/10.1111/jch.13130>.
6. Deiseroth A, Streese L, Köchli S, Wüst RS, Infanger D, Schmidt-Trucksäss A*,*  Exercise and arterial stiffness in the elderly: a combined cross-sectional and randomized controlled trial (EXAMIN AGE). Front Physiol. 2019; 10: 474298. <https://doi.org/10.3389/fphys.2019.01119>.
7. Zhang HM, Xie XF, Xiao Y, Effect of high-intensity interval training and medium-intensity continuous training on vascular health in patients with type 2 diabetes mellitus. Journal of medical Graduate students. 2022; 35(01): 63-68. https:// doi.org/ 10.16571/j.cnki.1008-8199.2022.01.012.
8. Kim HK, Hwang CL, Yoo JK, Hwang MH, Handberg EM, Petersen JW*,* All-Extremity Exercise Training Improves Arterial Stiffness in Older Adults. Med Sci Sports Exerc. 2017; 49(7): 1404-1411. <https://doi.org/10.1249/mss.0000000000001229>.
9. Bellia A, Iellamo F, De Carli E, Andreadi A, Padua E, Lombardo M*,* Exercise individualized by TRIMPi method reduces arterial stiffness in early onset type 2 diabetic patients: A randomized controlled trial with aerobic interval training. Int J Cardiol. 2017; 248: 314-319. <https://doi.org/10.1016/j.ijcard.2017.06.065>.
10. Way KL, Sabag A, Sultana RN, Baker MK, Keating SE, Lanting S*,* The effect of low-volume high-intensity interval training on cardiovascular health outcomes in type 2 diabetes: A randomised controlled trial. Int J Cardiol. 2020; 320: 148-154. <https://doi.org/10.1016/j.ijcard.2020.06.019>.
11. Adams SC, DeLorey DS, Davenport MH, Stickland MK, Fairey AS, North S, Effects of high-intensity aerobic interval training on cardiovascular disease risk in testicular cancer survivors: A phase 2 randomized controlled trial. Cancer. 2017; 123(20): 4057-4065. https://doi.org/10.1002/cncr.30859.
12. Taha MM, Aneis YM, Hasanin ME, Felaya EE, Aldhahi MI, Abdeen HAA. Effect of high intensity interval training on arterial stiffness in obese hypertensive women: a randomized controlled trial. Eur Rev Med Pharmacol Sci. 2023; 27(9): 4069-4079. <https://doi.org/10.26355/eurrev_202305_32314.>
13. Bouaziz W, Lang PO, Schmitt E, Leprêtre PM, Lefebvre F, Momas C, Effects of a short-term interval aerobic training program with recovery bouts on vascular function in sedentary aged 70 or over: A randomized controlled trial. Arch Gerontol Geriatr. 2019; 82: 217-225. https://doi.org/10.1016/j.archger.2019.02.017.
14. Millen AME, Norton GR, Avidon I, Woodiwiss AJ. Effects of short-term exercise-training on aortic systolic pressure augmentation in overweight and obese individuals. Eur J Appl Physiol. 2013; 113(7): 1793-1803. <https://doi.org/10.1007/s00421-013-2610-2>.
15. Kearney TM, Murphy MH, Davison GW, O'Kane MJ, Gallagher AM. Accumulated brisk walking reduces arterial stiffness in overweight adults: Evidence from a randomized control trial. J Am Soc Hypertens. 2014; 8(2): 117-126. <https://doi.org/10.1016/j.jash.2013.10.001>.
16. Nualnim N, Parkhurst K, Dhindsa M, Tarumi T, Vavrek J, Tanaka H. Effects of Swimming Training on Blood Pressure and Vascular Function in Adults >50 Years of Age. Am J Cardiol. 2012; 109(7): 1005-1010. <https://doi.org/10.1016/j.amjcard.2011.11.029>.
17. Zhou LY, Wu ZZ, Hong LF. Effect of 12-week aerobic exercise training on brachial-ankle, pulse wave velocity in elderly. 2010; 32(05): 356-358. https:// doi.org/ :10.3760/ema. j. issn. 0254-1424.2010.05.009
18. Madden KM, Lockhart C, Cuff D, Potter TF, Meneilly GS. Aerobic training-induced improvements in arterial stiffness are not sustained in older adults with multiple cardiovascular risk factors. J Hum Hypertens. 2013; 27(5): 335-339. <https://doi.org/10.1038/jhh.2012.38>.
19. Horner K, Barinas-Mitchell E, DeGroff C, Kuk JL, Drant S, Lee S. Effect of aerobic versus resistance exercise on pulse wave velocity, intima media thickness and left ventricular mass in obese adolescents. Pediatr Exerc Sci. 2015; 27(4): 494-502. <https://doi.org/10.1123/pes.2015-0067>.
20. Huang CY, Pan ML, Zhang L, Effect of maximum fat oxidation intensity exercise on arterial stiffness in overweight/obese young men. 2018; 37(01): 3-9. https:// doi.org/ 10.16038/j.1000-6710.2018.01.001.
21. Shin J-H, Lee Y, Kim SG, Choi BY, Lee H-S, Bang S-Y. The beneficial effects of Tai Chi exercise on endothelial function and arterial stiffness in elderly women with rheumatoid arthritis. Arthritis Res Ther. 2015; 17(1): 380. https://doi.org/10.1186/s13075-015-0893-x.
22. Davis CL, Litwin SE, Pollock NK, Waller JL, Zhu H, Dong Y*,* Exercise effects on arterial stiffness and heart health in children with excess weight: The SMART RCT. Int J Obes. 2020; 44(5): 1152-1163. <https://doi.org/10.1038/s41366-019-0482-1>.
23. Chen TT, Li lC, Yin XL, Study on the influence of Taijiquan on PWV, ABL and ADP in middle-aged and elderly people. 2018; 35(18): 134-135+150. https:// doi.org/10.3969/j.issn.1674-151x.2018.18.067
24. Madden KM, Lockhart C, Cuff D, Potter TF, Meneilly GS. Short-term aerobic exercise reduces arterial stiffness in older adults with type 2 diabetes, hypertension, and hypercholesterolemia. Diabetes Care. 2009; 32(8): 1531-1535. https://doi.org/10.2337/dc09-0149.
25. O'Connor EM, Koufaki P, Mercer TH, Lindup H, Nugent E, Goldsmith D, Long-term pulse wave velocity outcomes with aerobic and resistance training in kidney transplant recipients - A pilot randomised controlled trial. PLoS One. 2017; 12(2): e0171063. https://doi.org/10.1371/journal.pone.0171063.
26. Jaime SJ. The Effects of Twelve Weeks of Whole-Body Vibration Training and Low-Intensity Resistance Exercise Training on Arterial Function, Muscle Strength, and Physical Performance in Dynapenic Postmenopausal Women. 2017.
27. Croymans DM, Krell SL, Oh CS, Katiraie M, Lam CY, Harris RA*,* Effects of resistance training on central blood pressure in obese young men. Randomized Controlled Trial. 2014; 28(3): 157-164. <https://doi.org/10.1038/jhh.2013.81>.
28. DeVallance E, Fournier S, Lemaster K, Moore C, Asano S, Bonner D*,* The effects of resistance exercise training on arterial stiffness in metabolic syndrome. Eur J Appl Physiol. 2016; 116(5): 899-910. <https://doi.org/10.1007/s00421-016-3348-4>.
29. Greenwood SA, Koufaki P, Mercer TH, Rush R, O'Connor E, Tuffnell R, Aerobic or Resistance Training and Pulse Wave Velocity in Kidney Transplant Recipients: A 12-Week Pilot Randomized Controlled Trial (the Exercise in Renal Transplant [ExeRT] Trial). Am J Kidney Dis. 2015; 66(4): 689-698. https://doi.org/10.1053/j.ajkd.2015.06.016.
30. Beck DT, Martin JS, Casey DP, Braith RW. Exercise Training Reduces Peripheral Arterial Stiffness and Myocardial Oxygen Demand in Young Prehypertensive Subjects. American Journal of Hypertension. 2013; 26(9): 1093-1102. <https://doi.org/10.1093/ajh/hpt080>.
31. Fernandez-del-Valle M, Gonzales JU, Kloiber S, Mitra S, Klingensmith J, Larumbe-Zabala E. Effects of resistance training on MRI-derived epicardial fat volume and arterial stiffness in women with obesity: a randomized pilot study. Eur J Appl Physiol. 2018; 118(6): 1231-1240. <https://doi.org/10.1007/s00421-018-3852-9>.
32. Jaime SJ, Maharaj A, Alvarez-Alvarado S, Figueroa A. Impact of low-intensity resistance and whole-body vibration training on aortic hemodynamics and vascular function in postmenopausal women. Hypertens Res. 2019; 42(12): 1979-1988. <https://doi.org/10.1038/s41440-019-0328-1>.
33. Ramírez-Vélez R, Castro-Astudillo K, Correa-Bautista JE, González-Ruíz K, Izquierdo M, García-Hermoso A*,* The Effect of 12 Weeks of Different Exercise Training Modalities or Nutritional Guidance on Cardiometabolic Risk Factors, Vascular Parameters, and Physical Fitness in Overweight Adults: Cardiometabolic High-Intensity Interval Training-Resistance Training Randomized Controlled Study. J Strength Cond Res. 2020; 34(8). <https://doi.org/10.1519/JSC.00000000000003533>.
34. Hetherington-Rauth M, Magalhães JP, Júdice PB, Melo X, Sardinha LB. Vascular improvements in individuals with type 2 diabetes following a 1 year randomised controlled exercise intervention, irrespective of changes in cardiorespiratory fitness. Diabetologia. 2020; 63(4): 722-732. <https://doi.org/10.1007/s00125-020-05089-5>.
35. Wong A, Sanchez-Gonzalez MA, Son W-M, Kwak Y-S, Park S-Y. The effects of a 12-week combined exercise training program on arterial stiffness, vasoactive substances, inflammatory markers, metabolic profile, and body composition in obese adolescent girls. Pediatr Exerc Sci. 2018; 30(4): 480-486. <https://doi.org/10.1123/pes.2017-0198>.
36. Magalhães JP, Melo X, Correia IR, Ribeiro RT, Raposo J, Dores H*,* Effects of combined training with different intensities on vascular health in patients with type 2 diabetes: a 1-year randomized controlled trial. Cardiovasc Diabetol. 2019; 18(1): 34. <https://doi.org/10.1186/s12933-019-0840-2>.
37. Lee YH, Park SH, Yoon ES, Lee C-D, Wee SO, Fernhall B*,* Effects of Combined Aerobic and Resistance Exercise on Central Arterial Stiffness and Gait Velocity in Patients with Chronic Poststroke Hemiparesis. Am J Phys Med Rehabil. 2015; 94(9). <https://doi.org/10.1097/PHM.000000000000000233>.
38. Shiotsu Y, Watanabe Y, Tujii S, Yanagita M. Effect of exercise order of combined aerobic and resistance training on arterial stiffness in older men. Exp Gerontol. 2018; 111: 27-34. <https://doi.org/10.1016/j.exger.2018.06.020>.
39. Stewart KJ, Bacher AC, Turner KL, Fleg JL, Hees PS, Shapiro EP*,* Effect of Exercise on Blood Pressure in Older Persons: A Randomized Controlled Trial. Arch Intern Med. 2005; 165(7): 756-762. <https://doi.org/10.1001/archinte.165.7.756>.
40. Park W, Jung W-S, Hong K, Kim Y-Y, Kim S-W, Park H-Y. Effects of Moderate Combined Resistance- and Aerobic-Exercise for 12 Weeks on Body Composition, Cardiometabolic Risk Factors, Blood Pressure, Arterial Stiffness, and Physical Functions, among Obese Older Men: A Pilot Study. Int J Environ Res Public Health. 2020; 17(19): 7233. https://doi.org/10.3390/ijerph17197233.
41. Son W-M, Sung K-D, Cho J-M, Park S-Y. Combined exercise reduces arterial stiffness, blood pressure, and blood markers for cardiovascular risk in postmenopausal women with hypertension. Menopause. 2017; 24(3): 262-268. <https://doi.org/10.1097/gme.0000000000000765>.
42. Dobrosielski DA, Gibbs BB, Ouyang P, Bonekamp S, Clark JM, Wang N-Y*,* Effect of Exercise on Blood Pressure in Type 2 Diabetes: A Randomized Controlled Trial. J Gen Intern Med. 2012; 27(11): 1453-1459. <https://doi.org/10.1007/s11606-012-2103-8>.
43. Loimaala A, Groundstroem K, Rinne M, Nenonen A, Huhtala H, Parkkari J*,* Effect of Long-Term Endurance and Strength Training on Metabolic Control and Arterial Elasticity in Patients With Type 2 Diabetes Mellitus. Am J Cardiol. 2009; 103(7): 972-977. <https://doi.org/10.1016/j.amjcard.2008.12.026>.
